# Supplementary material for: SLC13A2 promotes hepatocyte metabolic remodeling and liver regeneration by enhancing de novo cholesterol biosynthesis
Source: EMBO J. 2025 Jan 17;44(5):1442–63. doi: 10.1038/s44318-025-00362-y (PMC11876347; doi:10.1038/s44318-025-00362-y)
Supplement: Supplementary file 1 — Appendix [file 44318_2025_362_MOESM1_ESM.pdf]

APPENDIX FIGURE S1 2

APPENDIX FIGURE S2 3

APPENDIX FIGURE S3 4

APPENDIX FIGURE S4 5

APPENDIX FIGURE S5 6

APPENDIX FIGURE S6 7

APPENDIX TABLE S1. 8

APPENDIX TABLE S2 10

APPENDIX TABLE S3 11

Appendix Figure S1

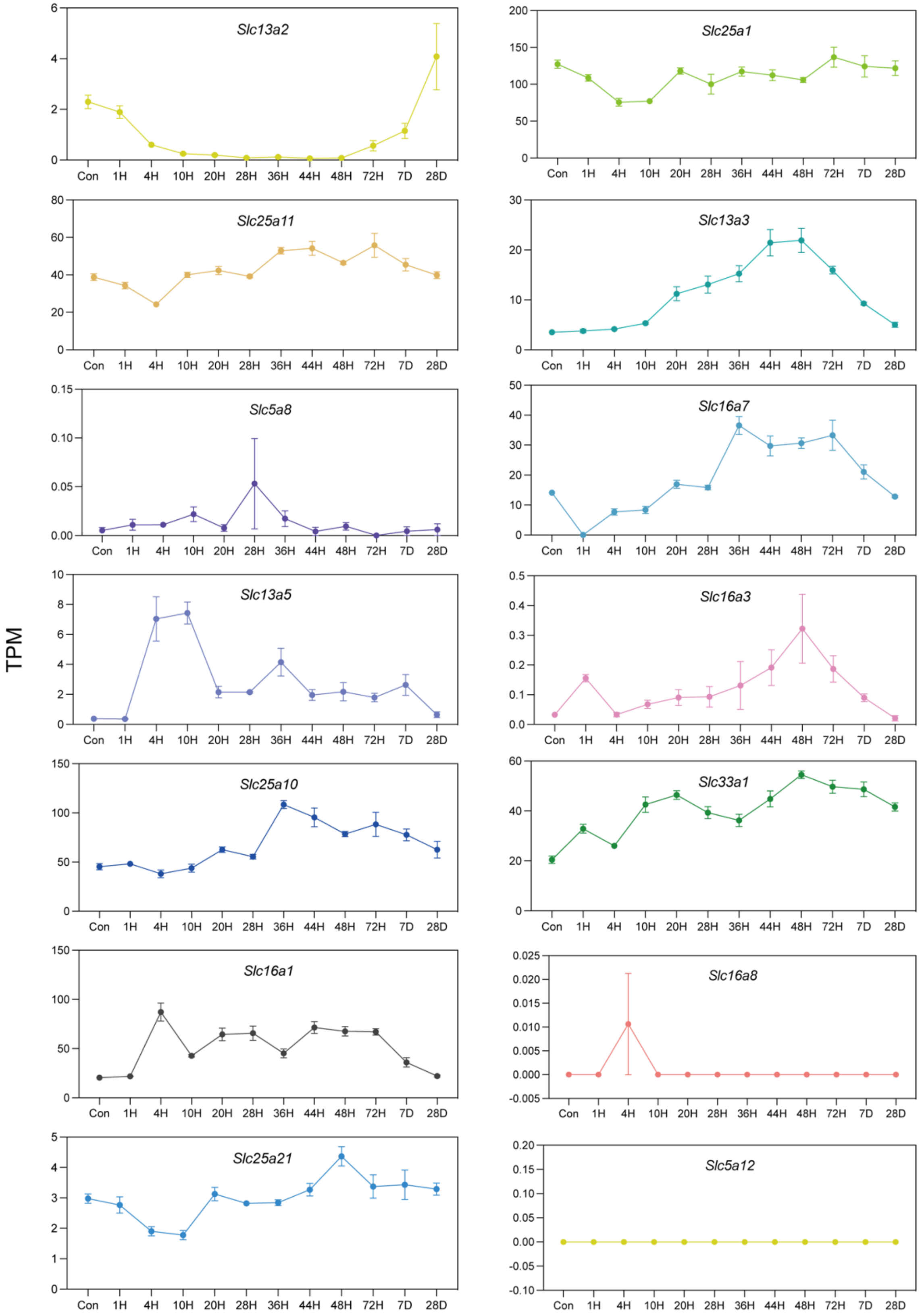

Appendix Figure S2

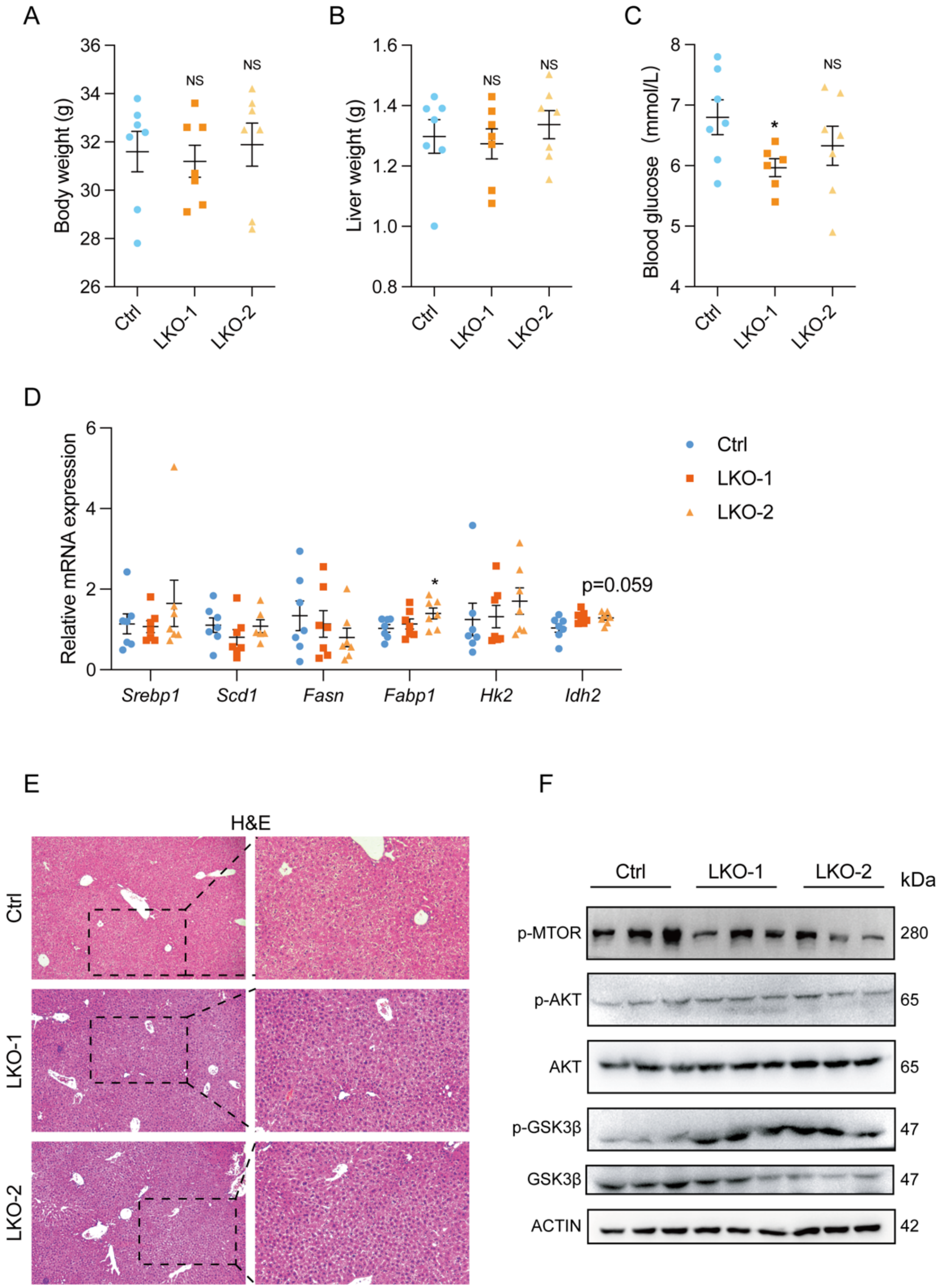

Appendix Figure S3

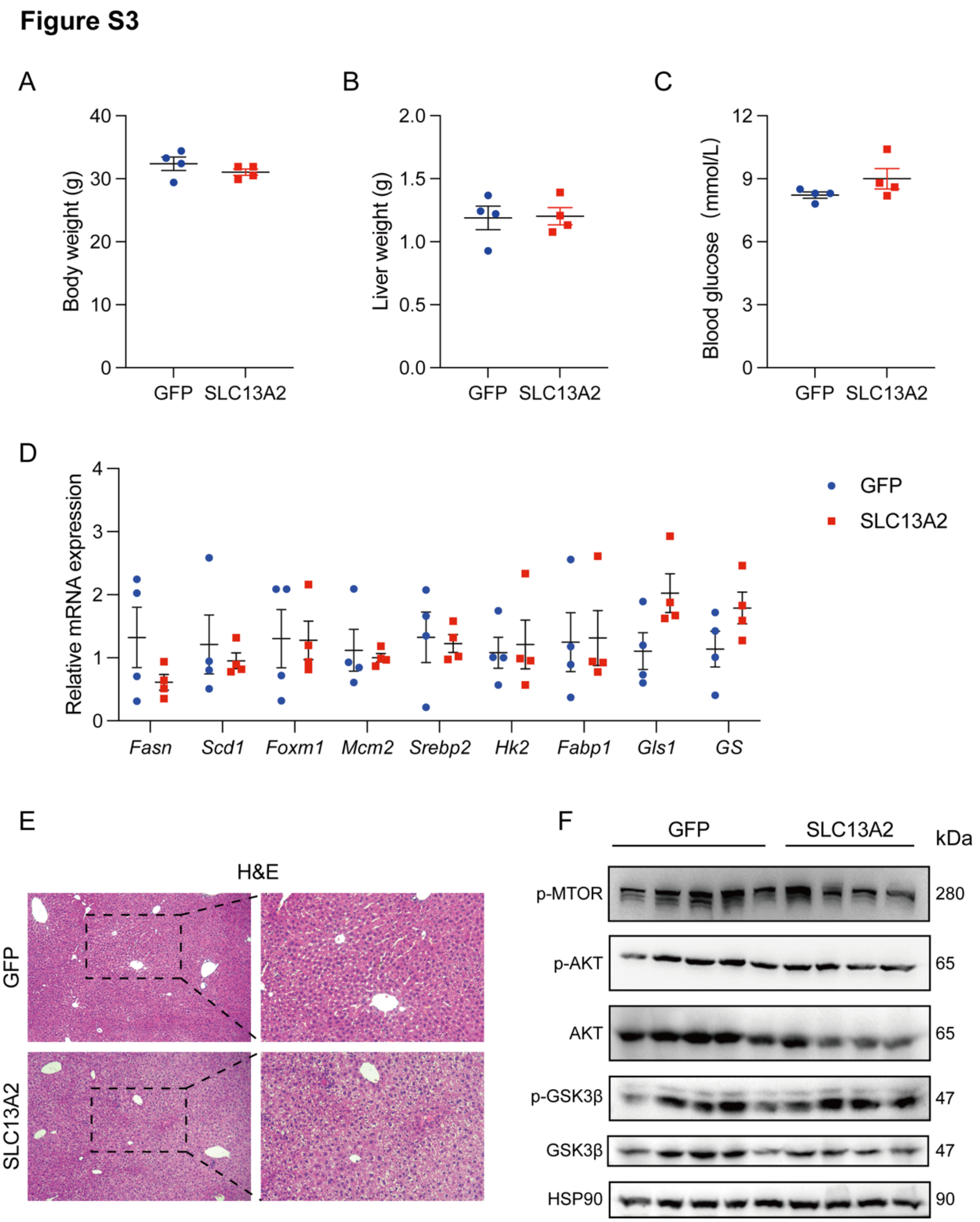

Appendix Figure S4

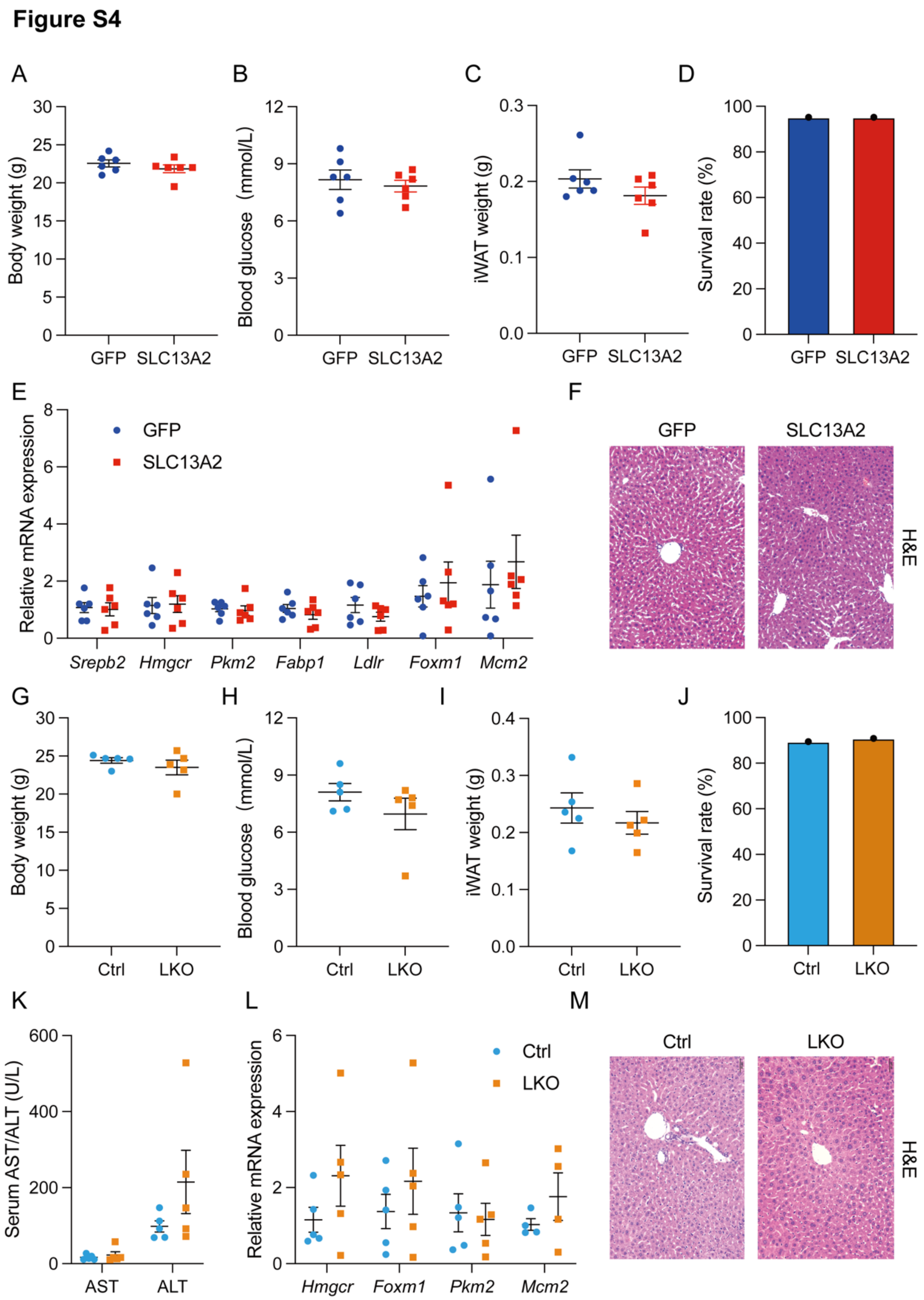

Appendix Figure S5

Figure S5

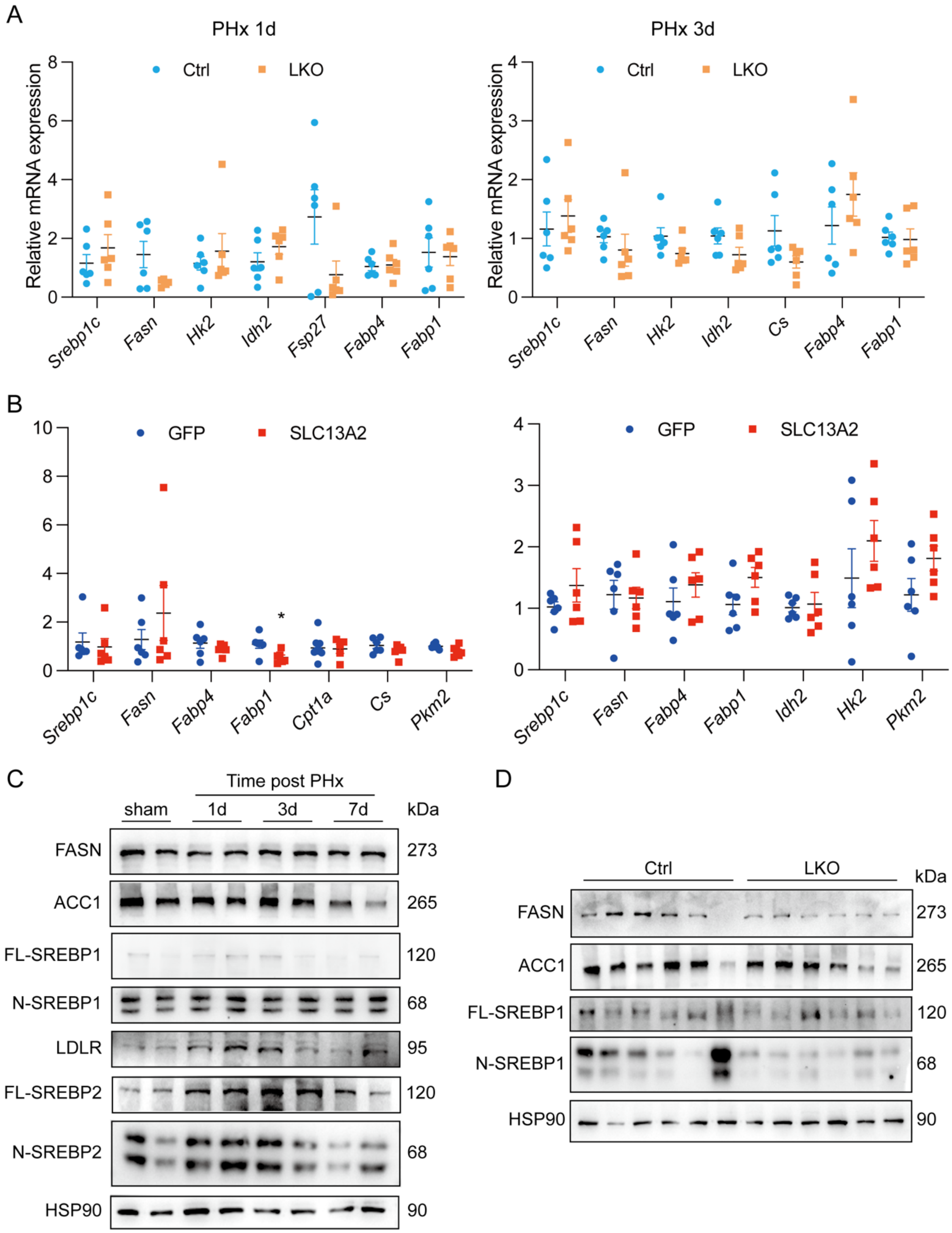

Appendix Figure S6

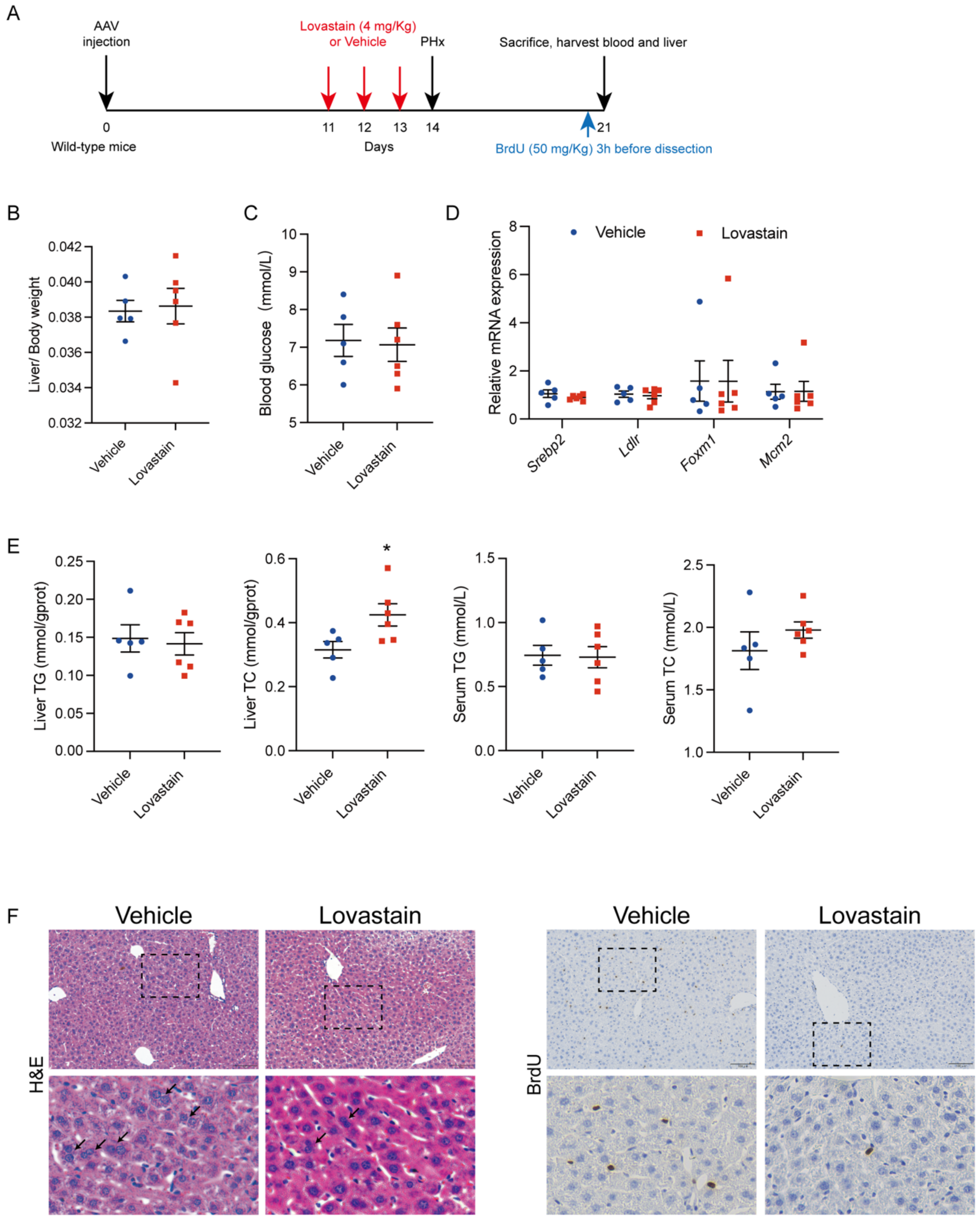

Appendix Table S1.

gRNA sequence targeting mouse SLC13A2 coding exons		
sgSLC13A2	ACCGCTGCTACTCAGCCAGCATTG	AACCAATGCTGGCTGAGTAGCAGC
Sequences of primers for construction		
Plasmid	Primers	Sequences
pAdeno-MCMV-SLC13A2-3Flag	pAdeno-SLC13A2-Flag-Forward	TCGAGCTCAAGCTTCGAATTCGTCCTCACCATGGCCACCTGCT
	pAdeno-SLC13A2-Flag-Reverse	GGCACTAGTACCGGTGAATTCGCTCACCATGACCGGTGGATCC
AAV-TBG-SLC13A2	AAV-TBG-SLC13A2-Forword	ATAACCGGTGTCCTCACCATGGCCACCTGCT
	AAV-TBG-SLC13A2- Reverse	ATAGGATCCCTAGTGGCCTGGTACAGTGGAA
Sequences of primers for qPCR analysis		
Genes	Primers	Sequences
Arpppo(P0)	Forward	GAAACTGCTGCCTCACATCCG
	Reverse	GCTGGCACAGTGACCTCACACG
mSlc13a2	Forward	CAGGCACTATGGGCCTATCG
	Reverse	AGGATGATGGAGTAAGCACAGT
mSlc5a8	Forward	GCCCCTTGAAACCTATGGCT
	Reverse	CAGTGGAGTCCTTTCCGCAT
mSlc5a12	Forward	CTGGGTGGCCATTGGATCTT
	Reverse	CAGCCCACAGCACTGAAGTA
mSlc13a3	Forward	GGAAGGCCGATGCCTCTATG
	Reverse	GGAAGTTGGTGTCGAGGAAGT
mSlc13a5	Forward	CTGTGGCTCCAGTGCTTGTA
	Reverse	GGGTGAAGCAAAAGAGCACG
mSlc16a1	Forward	GTGACTGGTCGGTCGTGTAG
	Reverse	GCAGCGCAAATCTAGTATCGTT
mSlc16a7	Forward	GTCCCCAACAAGTGGCTGAT
	Reverse	GGAGACGAAGGTGACCTGTT
mSlc16a8	Forward	CCAAGGCTGTTGGACACTCT
	Reverse	CTGCTTCTTCCTGCAAAGGC
mSlc16a3	Forward	GCACTTAAAGTCGCCCCCG
	Reverse	CACAAATGGTGTGCTGCCAA
mSlc25a1	Forward	TGCAGCCAGTGTCTTTGGAA
	Reverse	AGGATCTTCAAGCCGCAGTC
mSlc25a10	Forward	TACGAGACCATGCGGGACTA
	Reverse	AGGCTTTCTTCACGGGCTAC
mSlc25a11	Forward	GATTCAGGGCACACCAGACA
	Reverse	CTGTCAGCTGTTGAGTTACGA
mSlc25a21	Forward	CTGTGGCCTTCAATTTCTGGT
	Reverse	CCTTAGCTAGCTGCGACTGT
mSlc33a1	Forward	CACGGAACCCATTTTGGCAG
	Reverse	GACTGGTCGCTGTTTTGCAG
mSlc54a1	Forward	GCAAGGACTTCCGGGACTATC
	Reverse	TGGTCCTTGTACCGCTATGC
mSlc54a2	Forward	CACCTACCACCGACTCATGG
	Reverse	AGCACACACCAATCCCCATT
mSlc54a3	Forward	TGCTTGTAGAGCCCTCAGTT

	Reverse	TTTGTGCTTTTGCGCCTCTG
mCcnd1	Forward	CTGCTGCAAATGGAACTGCTT
	Reverse	TCATCCGCCTCTGGCATTTT
m*Ccnb1*	Forward	GGCTAACGGAAGTTGTCGAA
	Reverse	AAGGTGGCAACCACTGGAAA
m*Foxm1*	Forward	CGAGCACTTGGAATCACAGC
	Reverse	GGATGGGCACCAGGTATGAG
m*Cdc45*	Forward	TTATGGGACATCGTCGGCCA
	Reverse	TGCAGGATGCCAACATCAGT
m*Hk2*	Forward	CCGCCGTGGTGGACAAGATA
	Reverse	AGCAGTGATGAGAGCCGCTC
m*Cpt1α*	Forward	GAGAAATACCCTGACTATGTG
	Reverse	TGTGAGTCTGTCTCAGGGCTAG
m*Pkm2*	Forward	ATCATTTGTACCATTGGGCCTG
	Reverse	GCTCAGTACATCTTTCAAATTCGTG
m*Idh2*	Forward	GTAGATCGAGCTGGCACGTTC
	Reverse	TGGAAGCAGCTGTGCGCGAAGC
m*Fabp4*	Forward	TGCCTTTGTGGGAACCTG
	Reverse	GCTTGTCACCATCTCGTTTTC
m*Fabp1*	Forward	GAAGGCAATAGGTCTGCCCG
	Reverse	GTGAACTCATTGCGGACCAC
m*Cs*	Forward	TACTCACTGCAGCAACCCG
	Reverse	GCTCAGTACATCTTTCAAATTCGTG
m*Srebp1c*	Forward	CACCAGCATAGGCGAAGGA
	Reverse	ATGCCGACCAGATTCCCTAA
m*Srebp2*	Forward	CACCAGCATAGGCGAAGGA
	Reverse	ATGCCGACCAGATTCCCTAA
m*Scd1*	Forward	GCTGGAGTACGTCTGGAGGAA
	Reverse	TCCCGAAGAGGCAGGTGTAG
m*Fasn*	Forward	GGTTACACTGTGCTAGGTGTTG
	Reverse	TCCAGGCGCATGAGGCTCAGC
m*Ldlr*	Forward	GATGTCGACTGTGTTGACGGCTC
	Reverse	CTGACTTGTCCTTGCAGTCTGC
m*Mcm2*	Forward	CTCCAAGGCTGGCATCGTTA
	Reverse	GCCAGCATCTCATCCTGAACT
m*Hmgcr*	Forward	ATGTTCACCGGCAACAACAA
	Reverse	GCGATGCACCGCGTTATC
m*Fdft1*	Forward	GGATGTGACCTCCAAACAGGAC
	Reverse	CAGACCCATTGAGTTGGCACAC

Appendix Table S2

Antibodies	Manufacturers	Lot nos.
Rabbit-polyclonal-anti-SLC13A2	Designed and produced by Duoneng Bio. (Anhui, China)	N/A
Rabbit-monoclonal-anti-Phospho-SAP/JNK (Thr183/Tyr185) (81E11)	Cell Signaling Technology	4668S RRID: AB_823588
Rabbit-monoclonal-anti-Phospho-Akt (Ser473) (D9E)	Cell Signaling Technology	4060S RRID: AB_2315049
Rabbit-monoclonal-anti-Phospho-GSK-3β (Ser9) (5B3)	Cell Signaling Technology	9323P RRID: AB_2115201
Rabbit-polyclonal-anti-PCNA	Proteintech	10205-2-AP RRID: AB_2160330
Rabbit-polyclonal-anti-HSP90	Proteintech	13171-1-AP RRID: AB_2120924
Rabbit-monoclonal-anti-Phospho-ERK1-T202 + ERK2-T185	Abclonal	AP0485 RRID: AB_2863806
Rabbit-polyclonal-anti-Erk1/Erk2	Bioworld	BS1112 RRID: AB_1663392
Mouse-monoclonal-anti-SREBP2	Senta Cruz Biotechnology	sc-13552 RRID: AB_2194250
Mouse-monoclonal-anti-SREBP1	Senta Cruz Biotechnology	sc-13551 RRID: AB_628282
Rabbit-polyclonal-anti-LDLR	Proteintech	10785-1-AP RRID: AB_12281164
Rabbit-polyclonal-anti-HMGCR	Abclonal	A19063 RRID: AB_2862556
Rabbit-monoclonal-anti-Fatty Acid Synthase	Cell Signaling Technology	3180 RRID: AB_2100796
Mouse-monoclonal-anti-ACC	Proteintech	67373-1-Ig RRID: AB_2882621
Mouse-monoclonal-anti-BrdU	Proteintech	66241-1-Ig RRID: AB_2881630
Rabbit-recombinant anti-Ki67[B56]	Abcam	ab16667 RRID: AB_302459
HRP-conjugated Affinipure Goat Anti-Rabbit IgG(H+L)	Proteintech	SA00001-2 RRID: AB_2722564
HRP-conjugated Affinipure Goat Anti-Mouse IgG(H+L)	Proteintech	SA00001-1 RRID: AB_2722565

Appendix Table S3

REAGENT or RESOURCE	SOURCE	IDENTIFIER
**Antibodies**		
Antibodies, see Table S2		
**Bacterial and virus strains**		
AAV8-GFP virus	This paper	N/A
AAV8-SLC13A2 virus	This paper	N/A
pX602-AAV-LACZ virus	This paper	N/A
pX602-AAV-Cre-sgSLC13A2 virus	This paper	N/A
pAdeno-MCMV-3XFlag virus	OBiO	Cat#: GL2000
pAdeno-MCMV-SLC13A2-3XFlag virus	OBiO	Cat#: E5878
DH5α Chemically Competent Cell	Tsingke Biotech Co., Ltd.	Cat#: TSC-C14
NCM Stbl3 Competent Cell	New Cell & Molecular Biotech Co.,Ltd.	Cat#: MC012
**Chemicals, peptides, and recombinant proteins**		
Collagen type I	Corning	Cat#: 354236
Protease and Phosphatase inhibitor cocktail	Roche	Cat#: 11836153001
BMS-303141	TargetMol®	Cat#: 943962-47-8
Polyethylenimine	Poiysciences	Cat#: 19850
Opti-prep	Sigma-Aldrich	Cat#: D1556
Benzonase	Sigma-Aldrich	Cat#: 101654
Poloxamer 188 solution	Sigma-Aldrich	Cat#: P5556
Proteinase K	Beyotime Biotechnology	Cat#: ST533
Collagenase II	Biofroxx	Cat#: 2275MG100
5-bromo-2′-deoxyuridine	Beyotime Biotechnology	Cat#: ST1056
Cholesterol	Sigma-Aldrich	Cat#: C8667
Filipin complex	MCE	Cat#: HY-N6716
Isoflurane	Jiangsu H.F.Q. Biotechnology Co. Ltd.	Cat#: 20210801
Methanol HPLC grade	MERCK	Cat#: 67-56-1
Acetonitrile HPLC grade	MERCK	Cat#: 75-05-8
Lovastatin	Aladdin	Cat#: L107709
EGF	PEPROTECH	Cat#: AF-100-15
Ethylenediaminetetraacetic acid disodium salt, dihydrate	Solarbio	Cat#: E8030
4-Chloro-DL-phenylalanine	Sigma-Aldrich	Cat#: C6506
Crystal violet	Solarbio	Cat#: C8470
TRIzol reagent	Vazyme Biotech Co., Ltd.	Cat#: R401-01
Cell lysis buffer for Western an IP without inhibitors	New Cell & Molecular Biotech Co., Ltd.	Cat#: P70100
Sodium pyrophosphate	Beyotime Biotechnology	Cat#: ST640
BCA protein assay kit	Vazyme Biotech Co., Ltd.	Cat#: P0011
CCK8 assay kit	Vazyme Biotech Co., Ltd.	Cat#: A311-01
RNase A	Vazyme Biotech Co., Ltd.	Cat#: DE111-01-AA
Fetal bovine serum	TransGen Biotech	Cat#: FS401-02
DMEM	Sigma-Aldrich	Cat#: D5030
DMEM medium	KeyGen Biotechnology	Cat#: 12800017
**Critical commercial assays**		

PrimeScript™ RT reagent Kit	Vazyme Biotech Co., Ltd.	Cat#: Cat#: R122-01
qPCR SYBR Green Master Mix (High ROX Premixed) kit	AG Bio	Cat#: AG11719
Total cholesterol assay kit	Nanjing Jiancheng Bioengineering Institute	Cat#: A111-1-1
Triglyceride assay kit	Nanjing Jiancheng Bioengineering Institute	Cat#: A110-1-1
DAB Horseradish Peroxidase Color Development kit	Beyotime Biotechnology	Cat#: P0202
CCK-8 Cell Counting Kit	Vazyme Biotech Co., Ltd.	Cat#: A311-01
HighPure Maxi Plasmid Kit	TIANGEN Biotech (Beijing) Co., Ltd.	Cat#: DP116
EndoFree Plasmid Midi Kit	CWBIO	Cat#: CW2105
Alanine aminotransferase Assay Kit	Nanjing Jiancheng Bioengineering Institute	Cat#: C009-2-1
Aspartate aminotransferase Assay Kit	Nanjing Jiancheng Bioengineering Institute	Cat#: C010-2-1
**Oligonucleotides**		
Primers for qPCR, see Table S1	Tsingke Biotech Co., Ltd.	N/A
Guide RNA targeting sequence: gSLC13A2, see Table S1	Tsingke Biotech Co., Ltd.	N/A
**Recombinant DNA**		
pAdeno-MCMV-SLC13A2-3Flag	This paper	N/A
PAd Delta F6	Addgene	Cat#: 112867
pAAV2/8	Addgene	Cat#: 112864
pX602	Addgene	Cat#: 61593
pX602-AAV-Cre-SgSLC13A2	This paper	N/A
AAV-TBG-GFP	Addgene	Cat#: 105535
AAV-TBG-SLC13A2	This paper	N/A
**Experimental models: Cell lines**		
Mouse normal hepatocytes AML12	Institute of Biochemistry and Cell Biology of the Chinese Academy of Sciences	N/A
HEK293T	ATCC	Cat#: CRL-11268; RRID: CVCL_1926
**Experimental models: Organisms/strains**		
Mouse: Rosa26-flox-STOP-flox-Cas9 knockin mice	Jackson Lab	Cat#: 024857
Mouse: male C57BL/6J mice	Center for Comparative Medicine, Yangzhou University, Yangzhou，China	N/A
**Software and algorithms**		
Image J	National Institutes of Health	https://imagej.nih.gov/ij/
Graphpad prism 9	Graphpad Software	https://graphpad.com
R software	R Foundation Statutes	https://www.r-project.org/
