# Supplementary figures and images for: SLC13A2 promotes hepatocyte metabolic remodeling and liver regeneration by enhancing de novo cholesterol biosynthesis
Source: EMBO J. 2025 Jan 17;44(5):1442–63. doi: 10.1038/s44318-025-00362-y (PMC11876347; doi:10.1038/s44318-025-00362-y)

## Slide 1
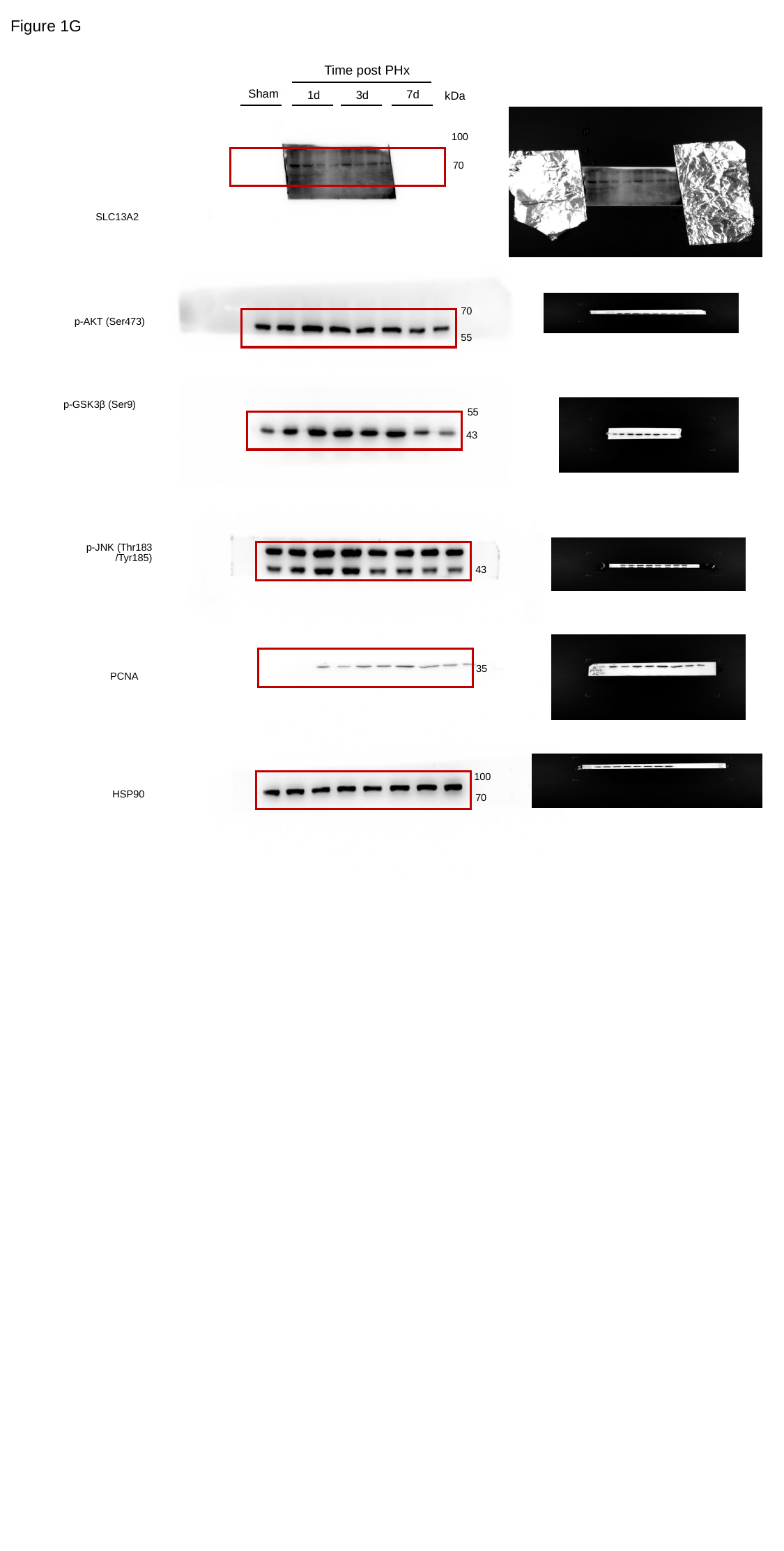

Figure 1G
Time post PHx
Sham
7d
1d
3d
kDa
100
70
SLC13A2
70
p-AKT (Ser473)
55
p-GSK3β (Ser9)
55
43
 p-JNK (Thr183
 /Tyr185)
43
35
PCNA
100
HSP90
70

Supplement: Supplementary file 3 — Source data Fig. 1 [file 44318_2025_362_MOESM3_ESM.zip › Figure 1/Figure 1G.pptx]

## Slide 1
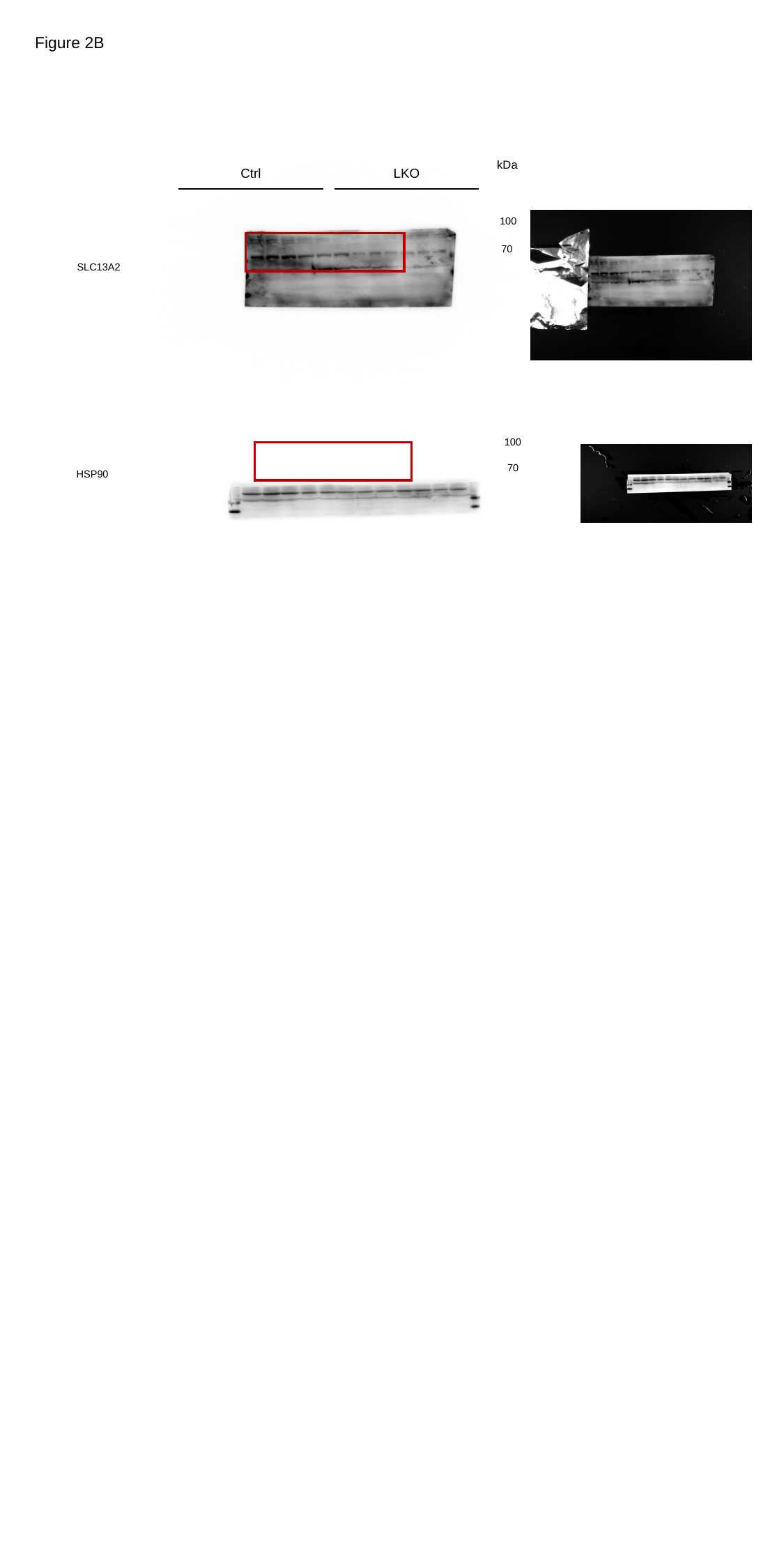

Figure 2B
kDa
Ctrl
LKO
100
70
SLC13A2
100
70
HSP90

Supplement: Supplementary file 4 — Source data Fig. 2 [file 44318_2025_362_MOESM4_ESM.zip › Figure 2/2B.pptx]

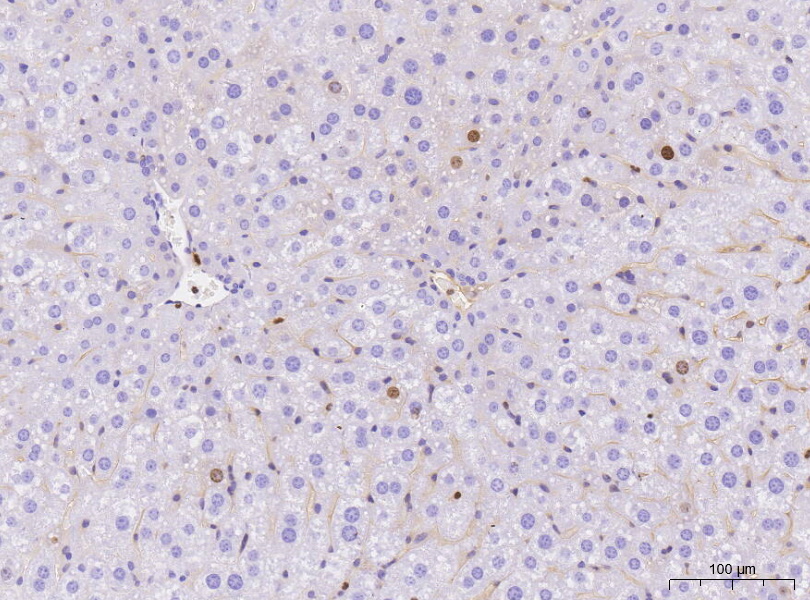

Supplement: Supplementary file 4 — Source data Fig. 2 [file 44318_2025_362_MOESM4_ESM.zip › Figure 2/2G/Ctrl-1.jpg]

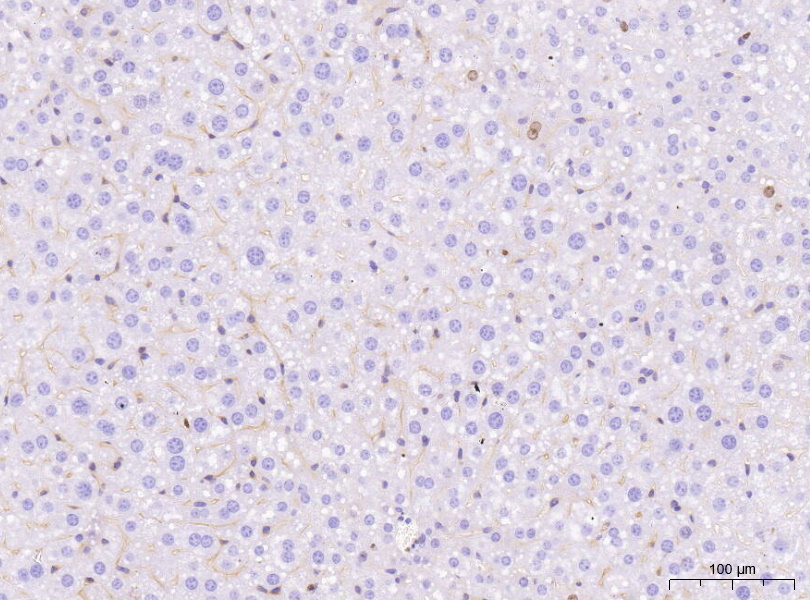

Supplement: Supplementary file 4 — Source data Fig. 2 [file 44318_2025_362_MOESM4_ESM.zip › Figure 2/2G/LKO-1.jpg]

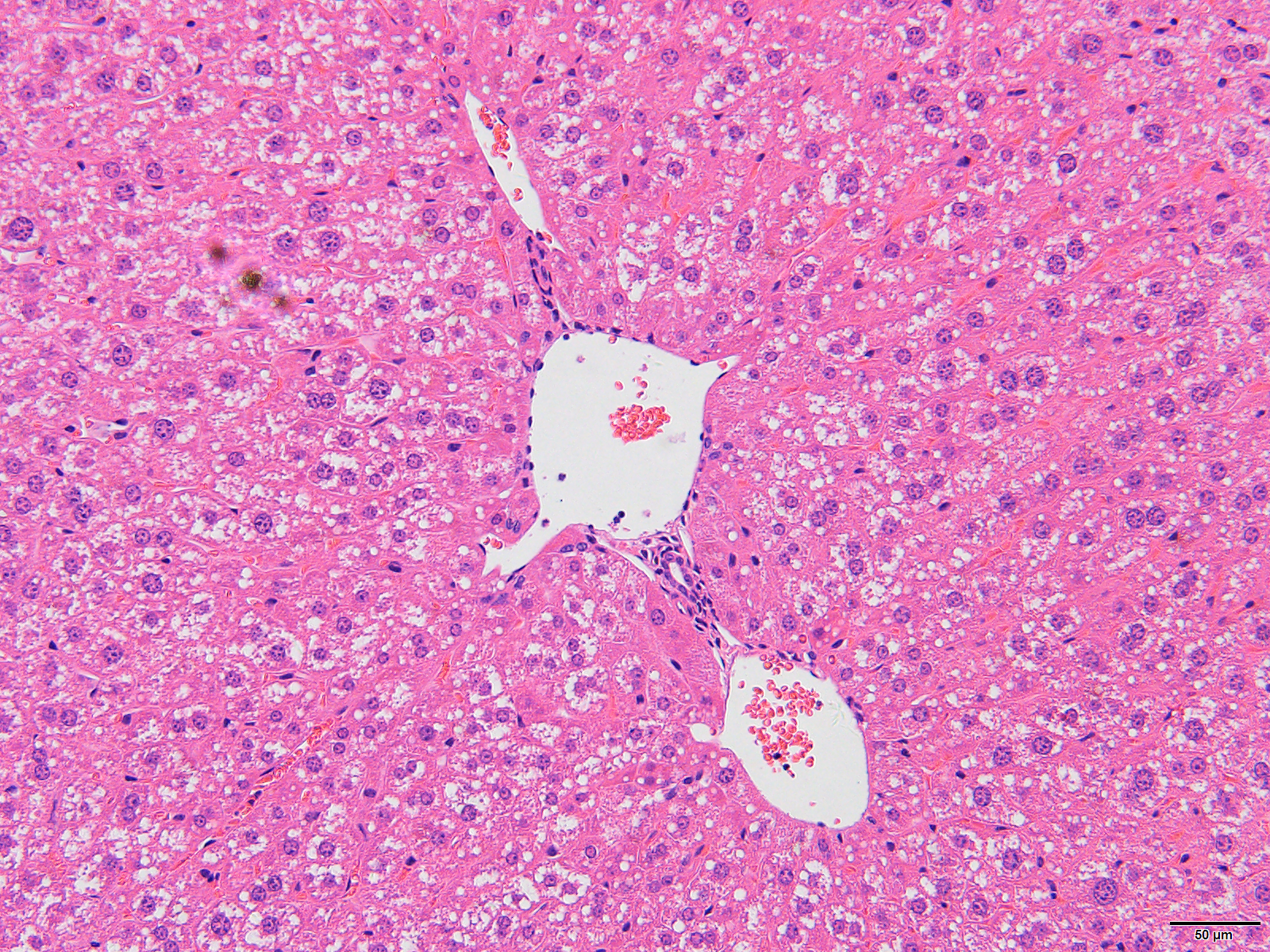

Supplement: Supplementary file 4 — Source data Fig. 2 [file 44318_2025_362_MOESM4_ESM.zip › Figure 2/2F/Ctrl-1.png]

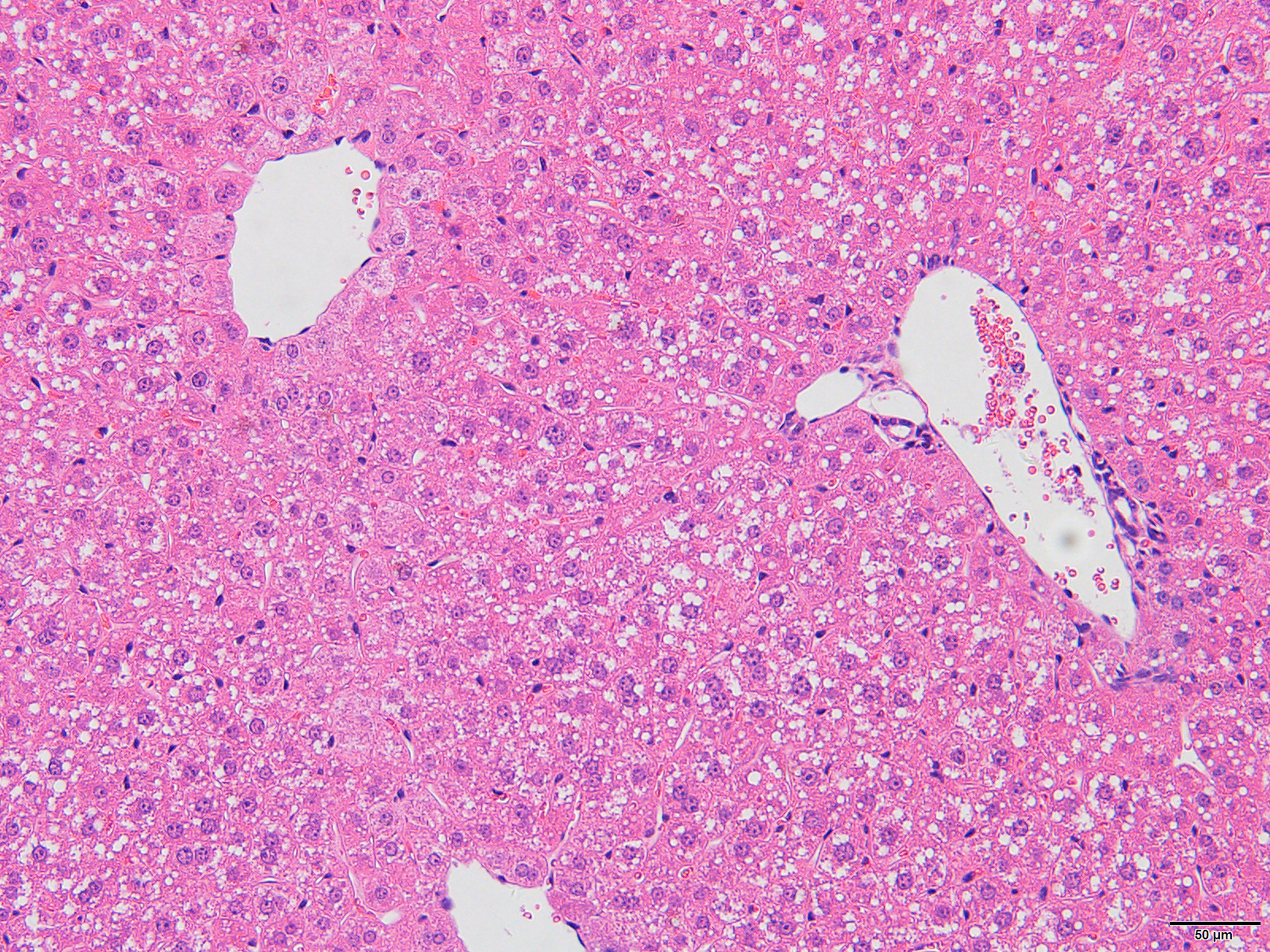

Supplement: Supplementary file 4 — Source data Fig. 2 [file 44318_2025_362_MOESM4_ESM.zip › Figure 2/2F/LKO-1.png]

## Slide 1
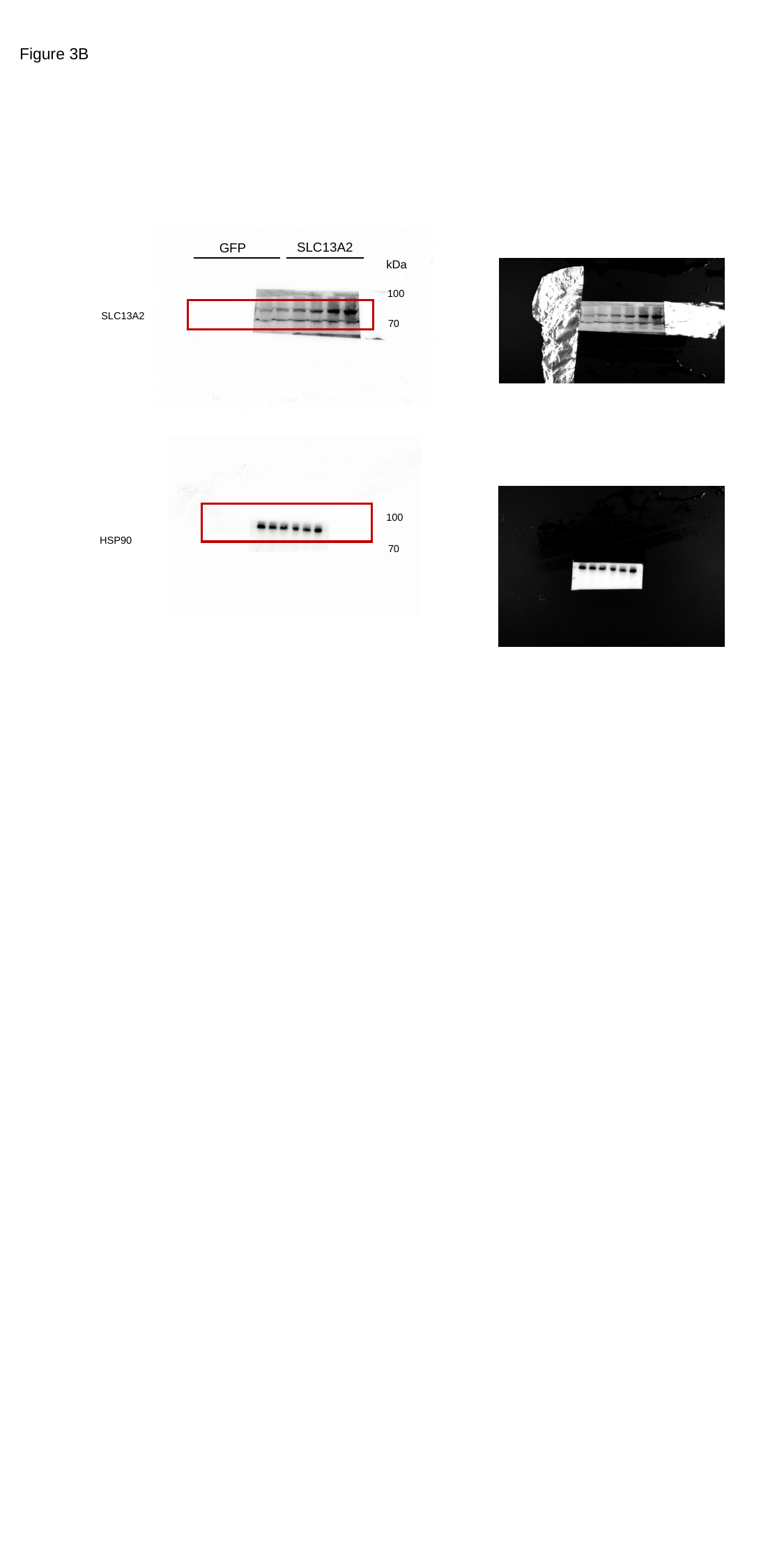

Figure 3B
SLC13A2
GFP
kDa
100
SLC13A2
70
100
HSP90
70

Supplement: Supplementary file 5 — Source data Fig. 3 [file 44318_2025_362_MOESM5_ESM.zip › Figure 3/3B.pptx]

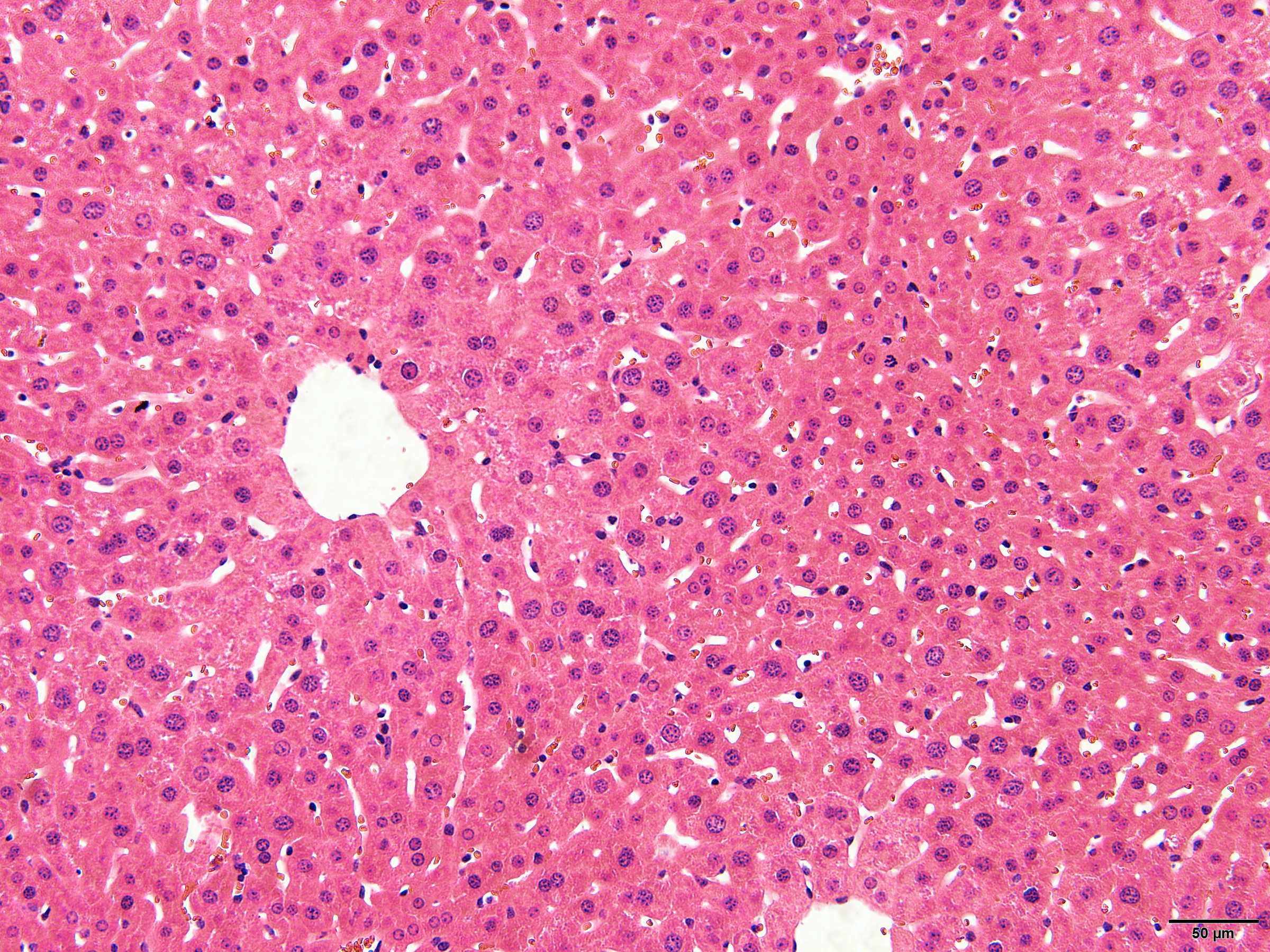

Supplement: Supplementary file 5 — Source data Fig. 3 [file 44318_2025_362_MOESM5_ESM.zip › Figure 3/3F/3d/SLC-1.tif]

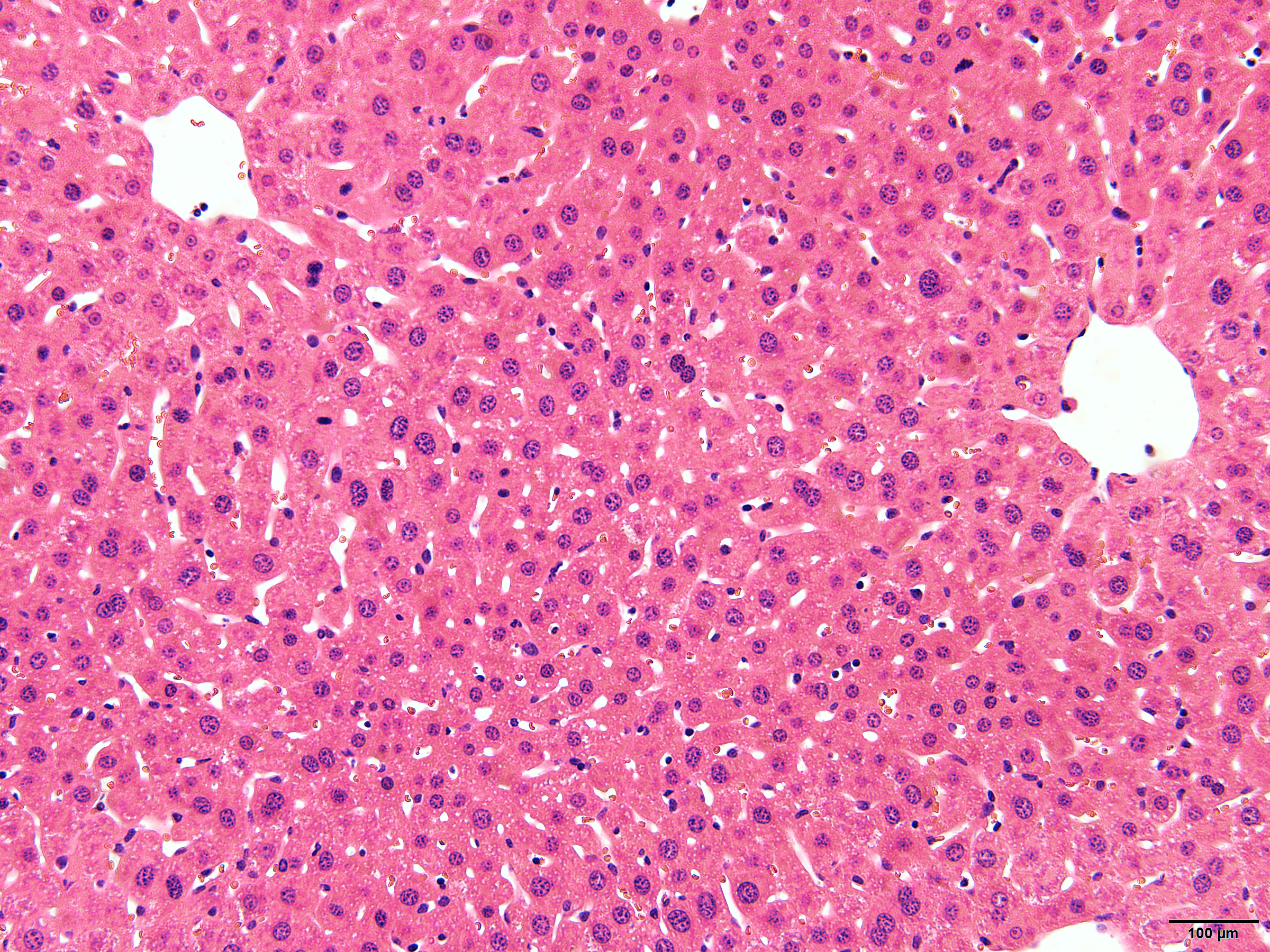

Supplement: Supplementary file 5 — Source data Fig. 3 [file 44318_2025_362_MOESM5_ESM.zip › Figure 3/3F/3d/GFP-1.tif]

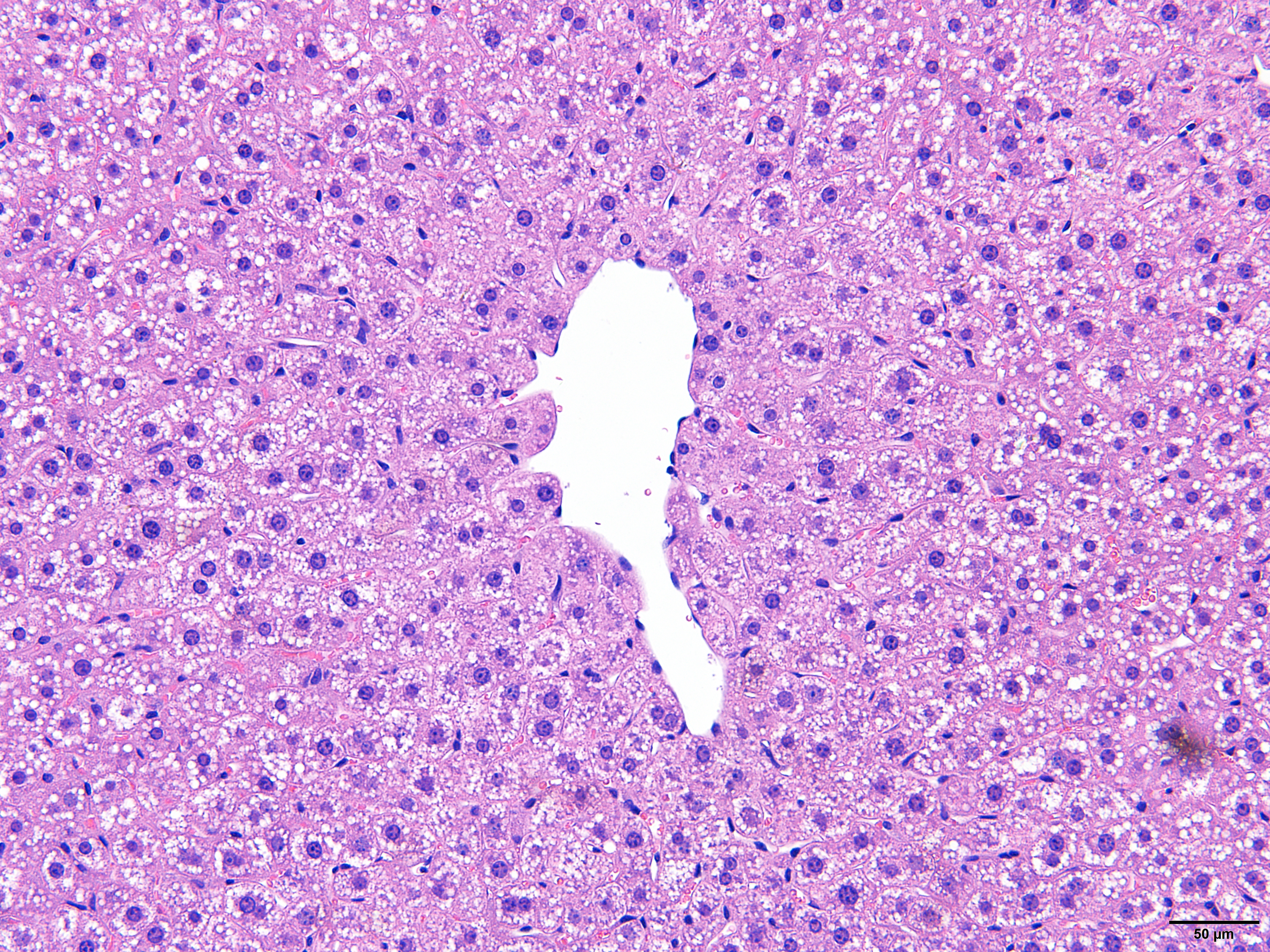

Supplement: Supplementary file 5 — Source data Fig. 3 [file 44318_2025_362_MOESM5_ESM.zip › Figure 3/3F/1d/SLC-1.tif]

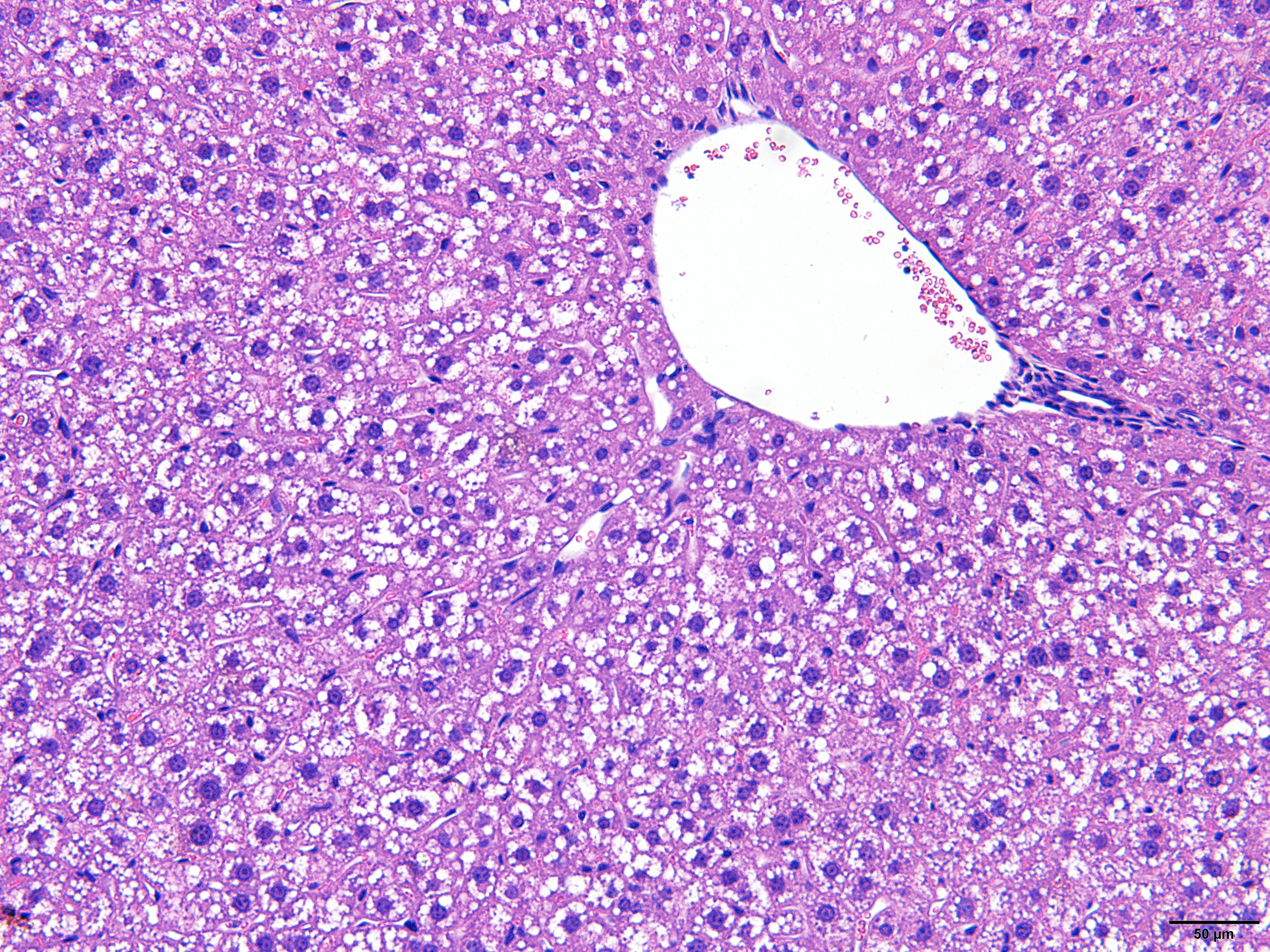

Supplement: Supplementary file 5 — Source data Fig. 3 [file 44318_2025_362_MOESM5_ESM.zip › Figure 3/3F/1d/GFP-1.tif]

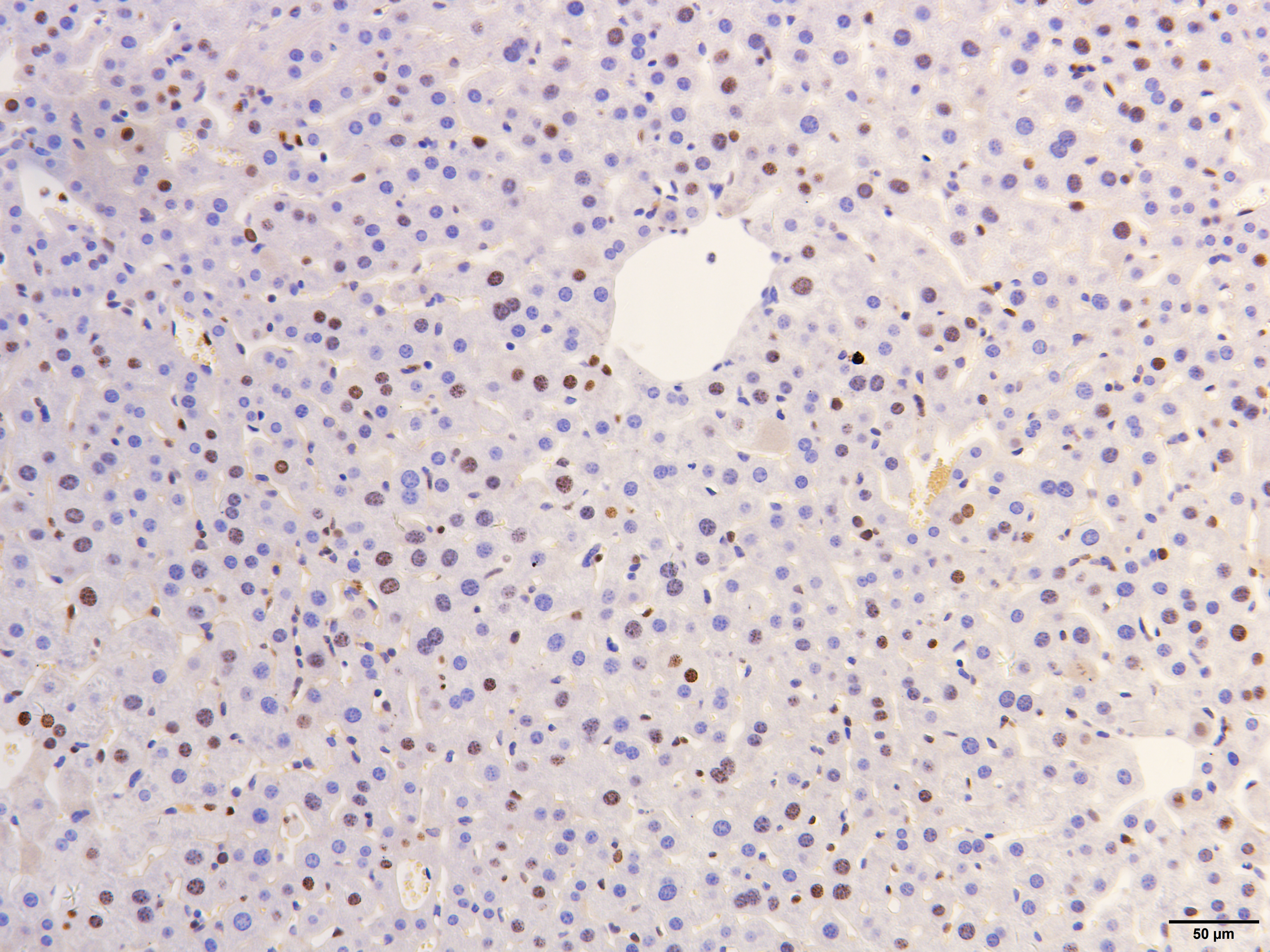

Supplement: Supplementary file 5 — Source data Fig. 3 [file 44318_2025_362_MOESM5_ESM.zip › Figure 3/3G/3d/SLC-1.tif]

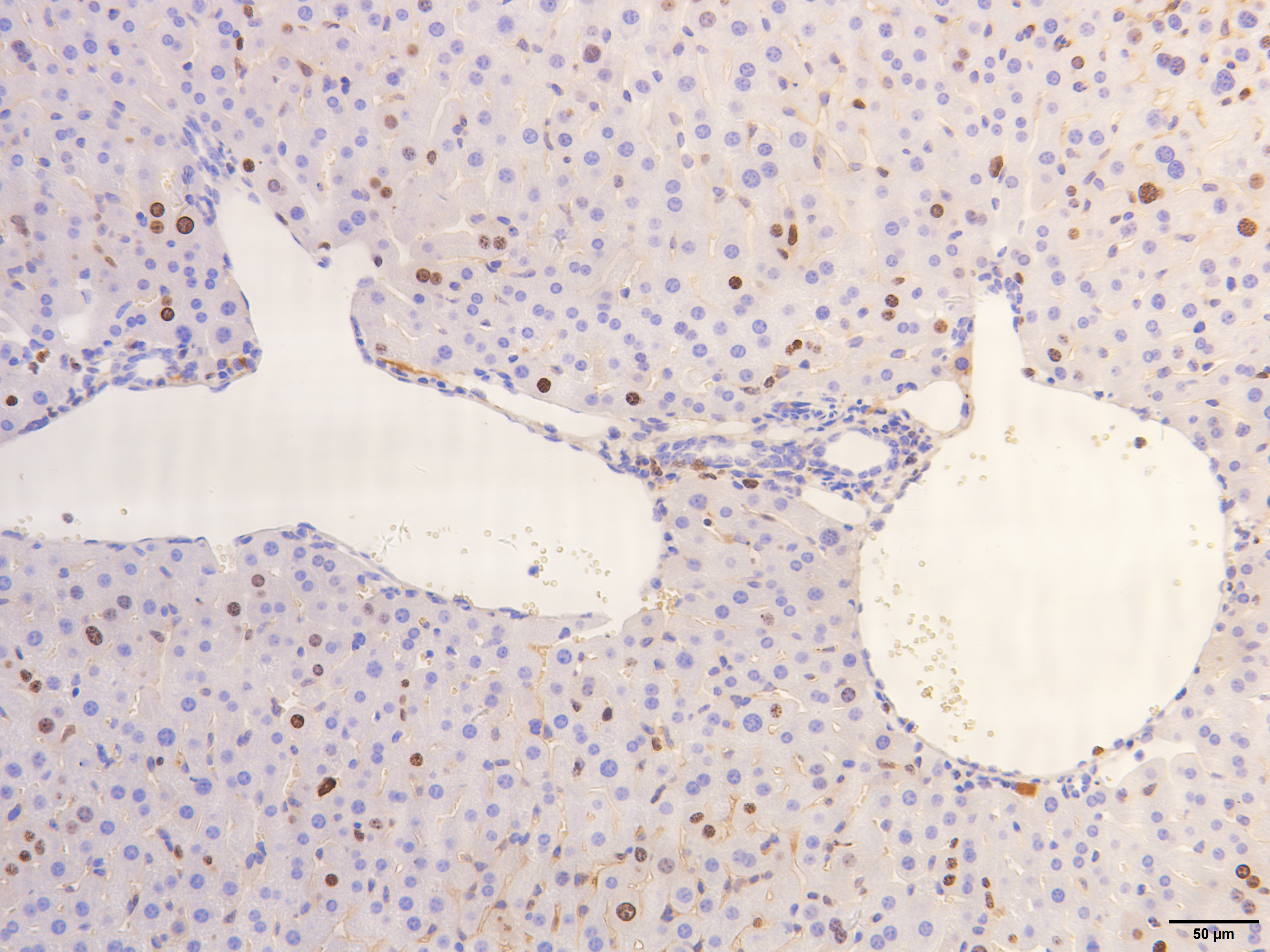

Supplement: Supplementary file 5 — Source data Fig. 3 [file 44318_2025_362_MOESM5_ESM.zip › Figure 3/3G/3d/GFP-1.tif]

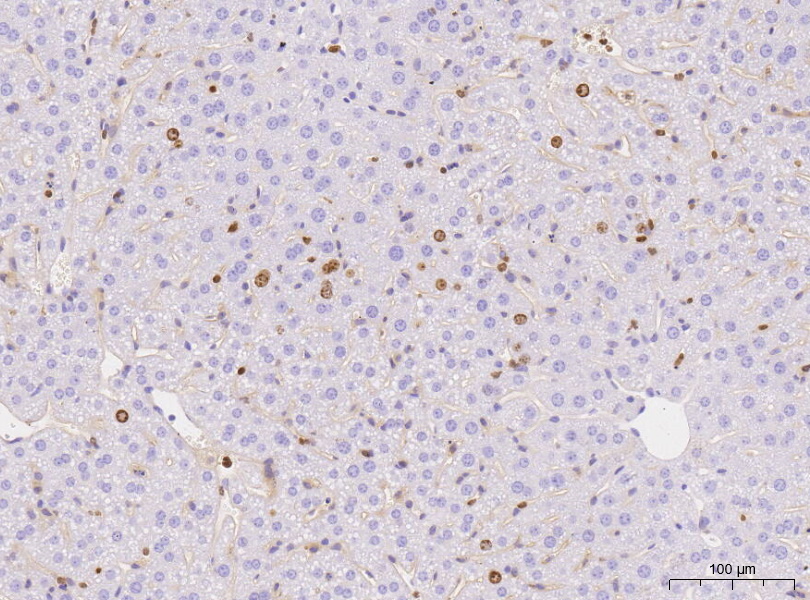

Supplement: Supplementary file 5 — Source data Fig. 3 [file 44318_2025_362_MOESM5_ESM.zip › Figure 3/3G/1d/SLC-1.jpg]

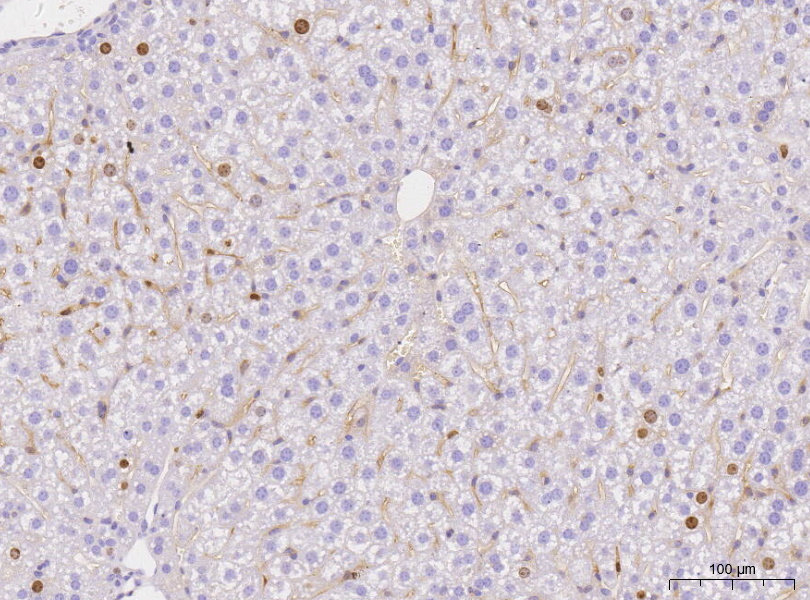

Supplement: Supplementary file 5 — Source data Fig. 3 [file 44318_2025_362_MOESM5_ESM.zip › Figure 3/3G/1d/GFP-1.jpg]

## Slide 1
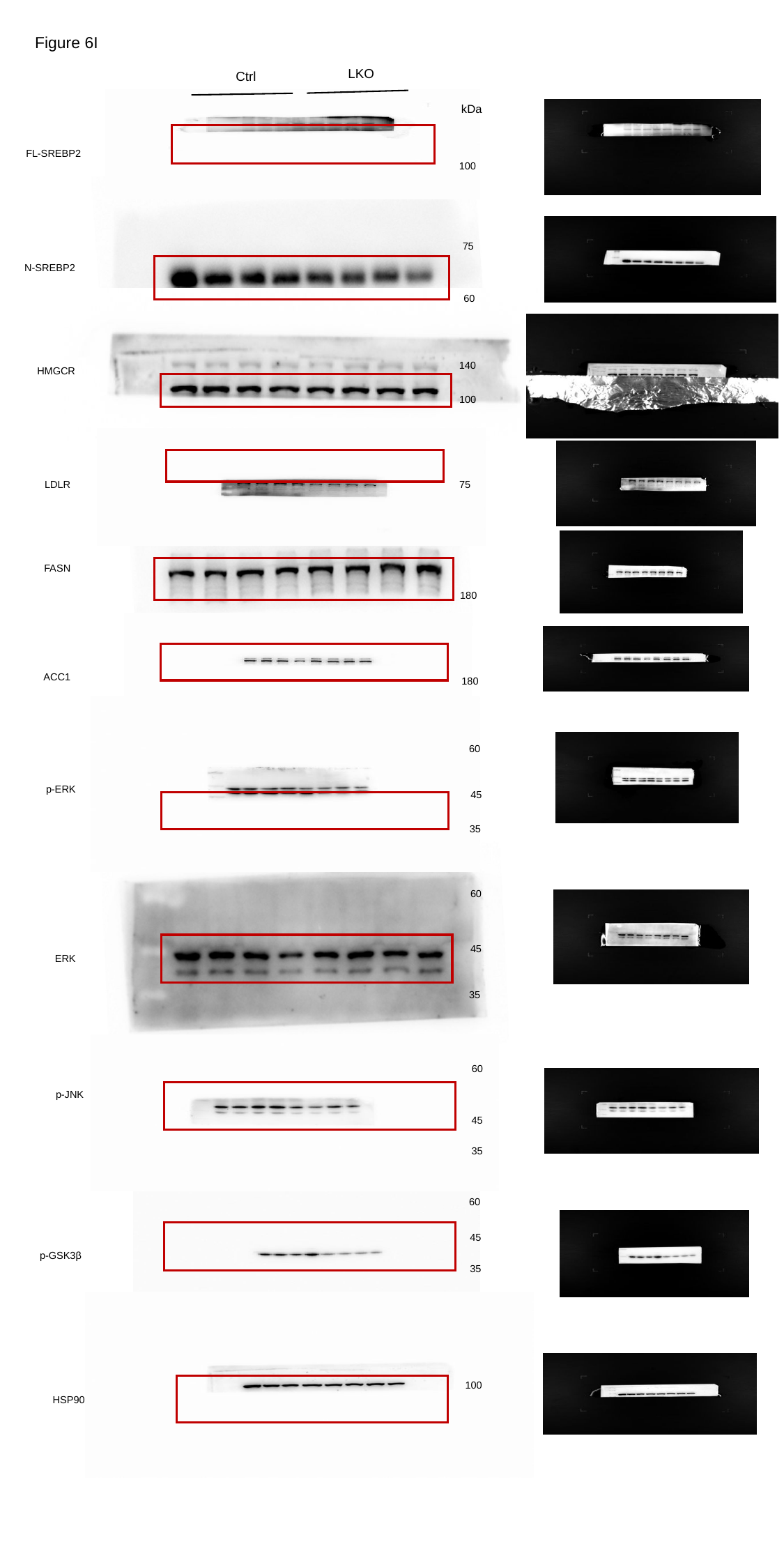

Figure 6I
LKO
Ctrl
kDa
FL-SREBP2
100
75
N-SREBP2
60
140
HMGCR
100
75
LDLR
FASN
180
ACC1
180
60
p-ERK
45
35
60
45
ERK
35
60
p-JNK
45
35
60
45
p-GSK3β
35
100
HSP90

Supplement: Supplementary file 8 — Source data Fig. 6 [file 44318_2025_362_MOESM8_ESM.zip › Figure 6/6I.pptx]

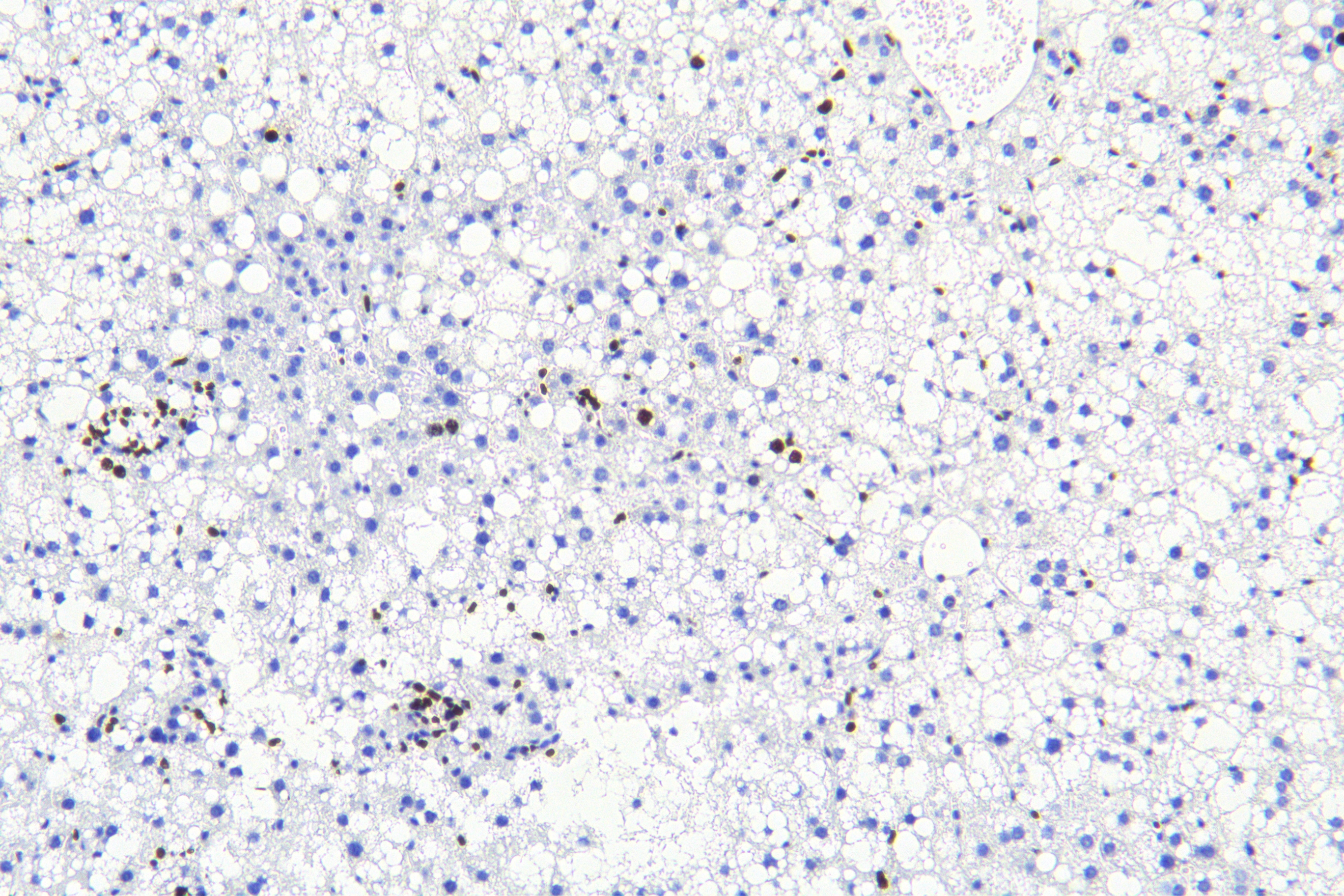

Supplement: Supplementary file 8 — Source data Fig. 6 [file 44318_2025_362_MOESM8_ESM.zip › Figure 6/6F/Ctrl-1.jpg]

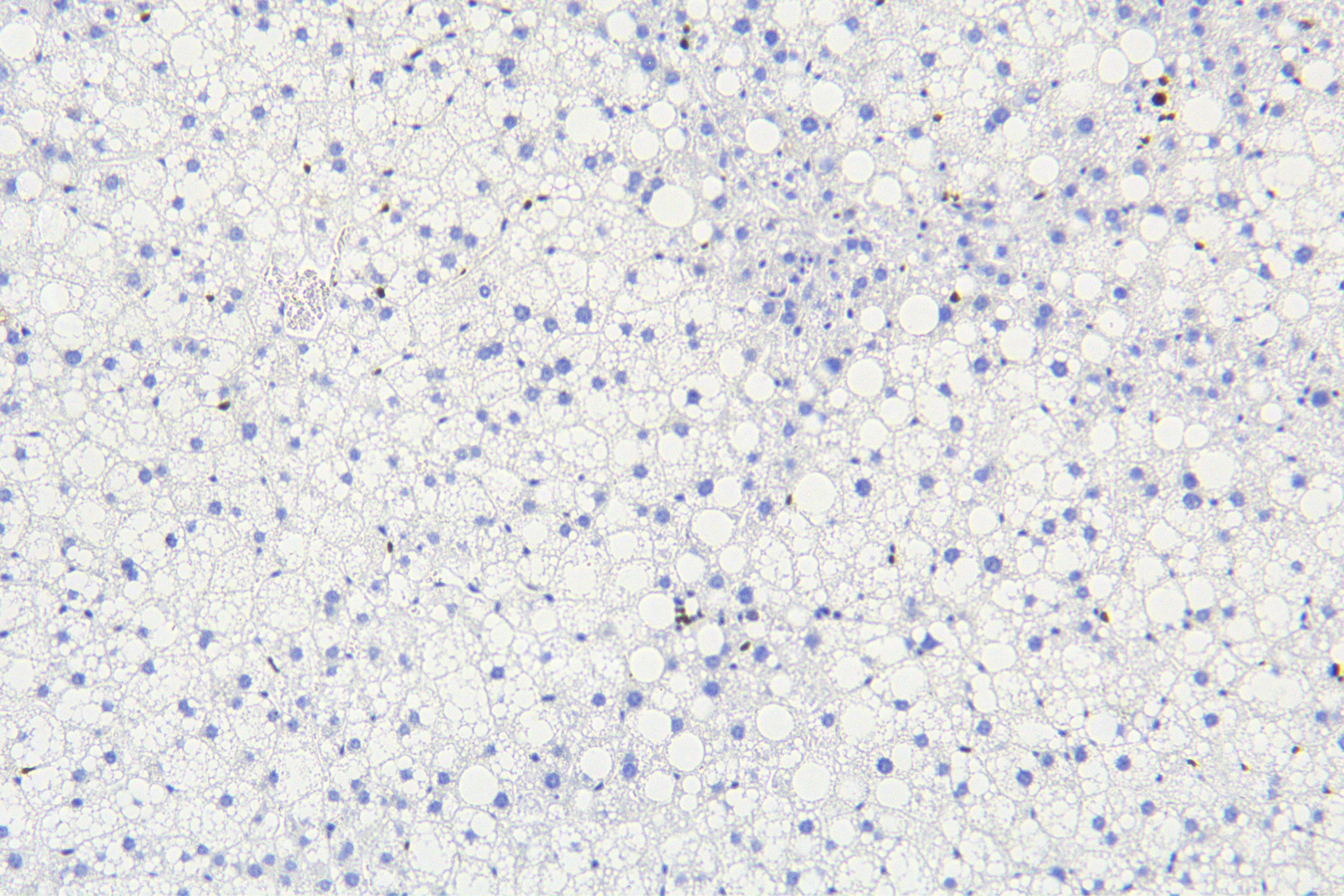

Supplement: Supplementary file 8 — Source data Fig. 6 [file 44318_2025_362_MOESM8_ESM.zip › Figure 6/6F/LKO-1.jpg]

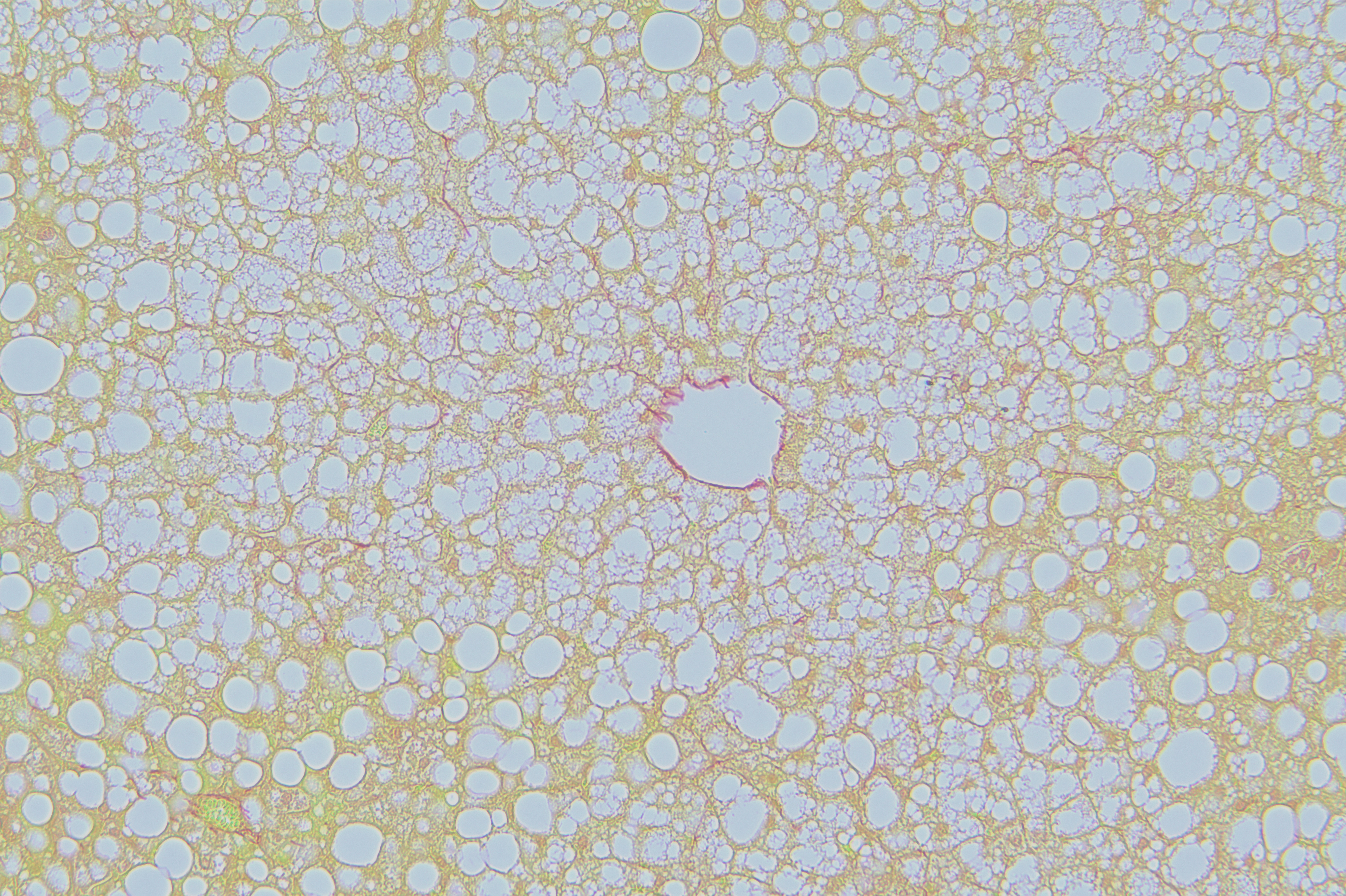

Supplement: Supplementary file 8 — Source data Fig. 6 [file 44318_2025_362_MOESM8_ESM.zip › Figure 6/6H/Ctrl-1.jpg]

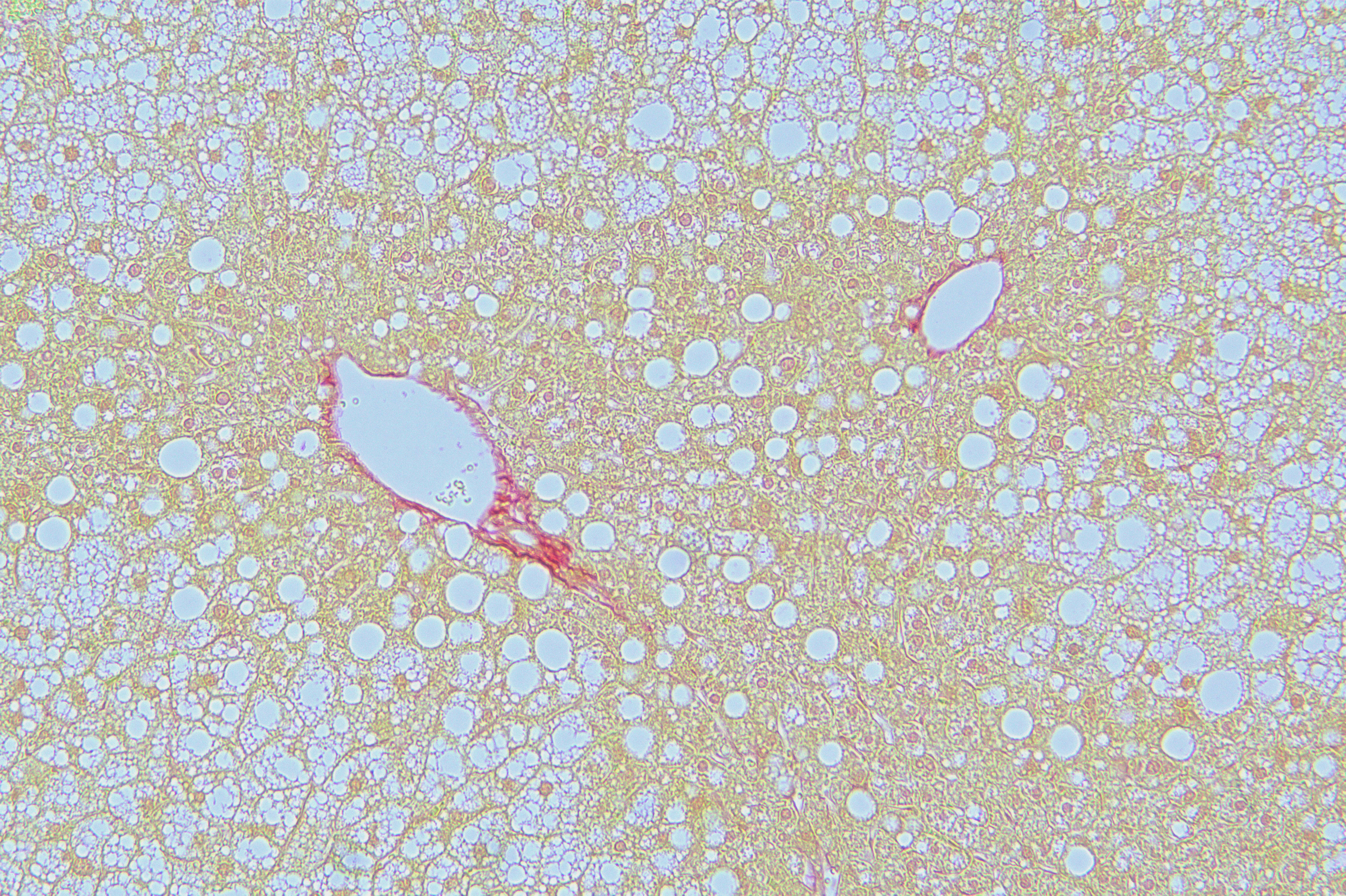

Supplement: Supplementary file 8 — Source data Fig. 6 [file 44318_2025_362_MOESM8_ESM.zip › Figure 6/6H/LKO-1.jpg]

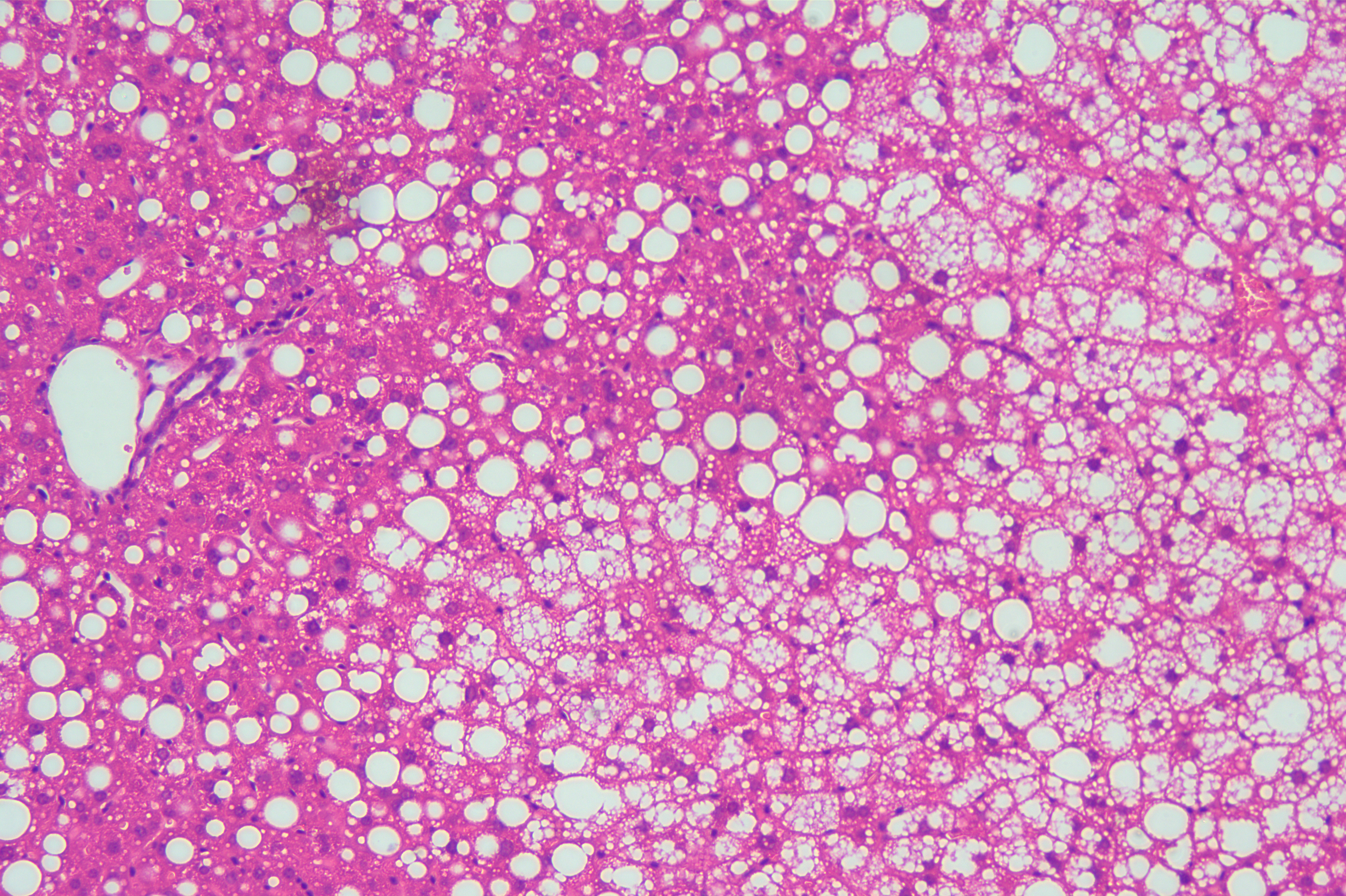

Supplement: Supplementary file 8 — Source data Fig. 6 [file 44318_2025_362_MOESM8_ESM.zip › Figure 6/6E/Ctrl-1.jpg]

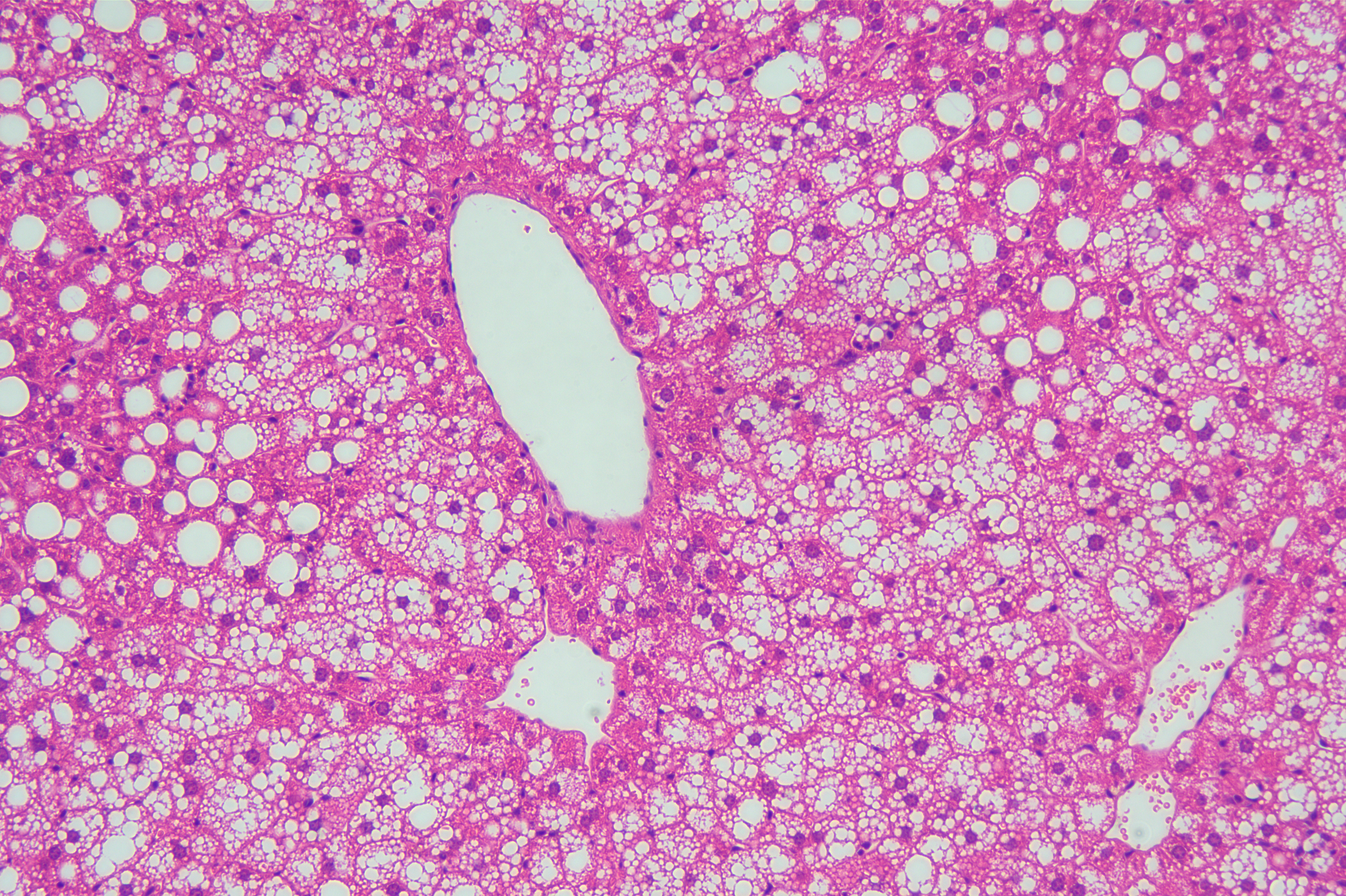

Supplement: Supplementary file 8 — Source data Fig. 6 [file 44318_2025_362_MOESM8_ESM.zip › Figure 6/6E/LKO-1.jpg]

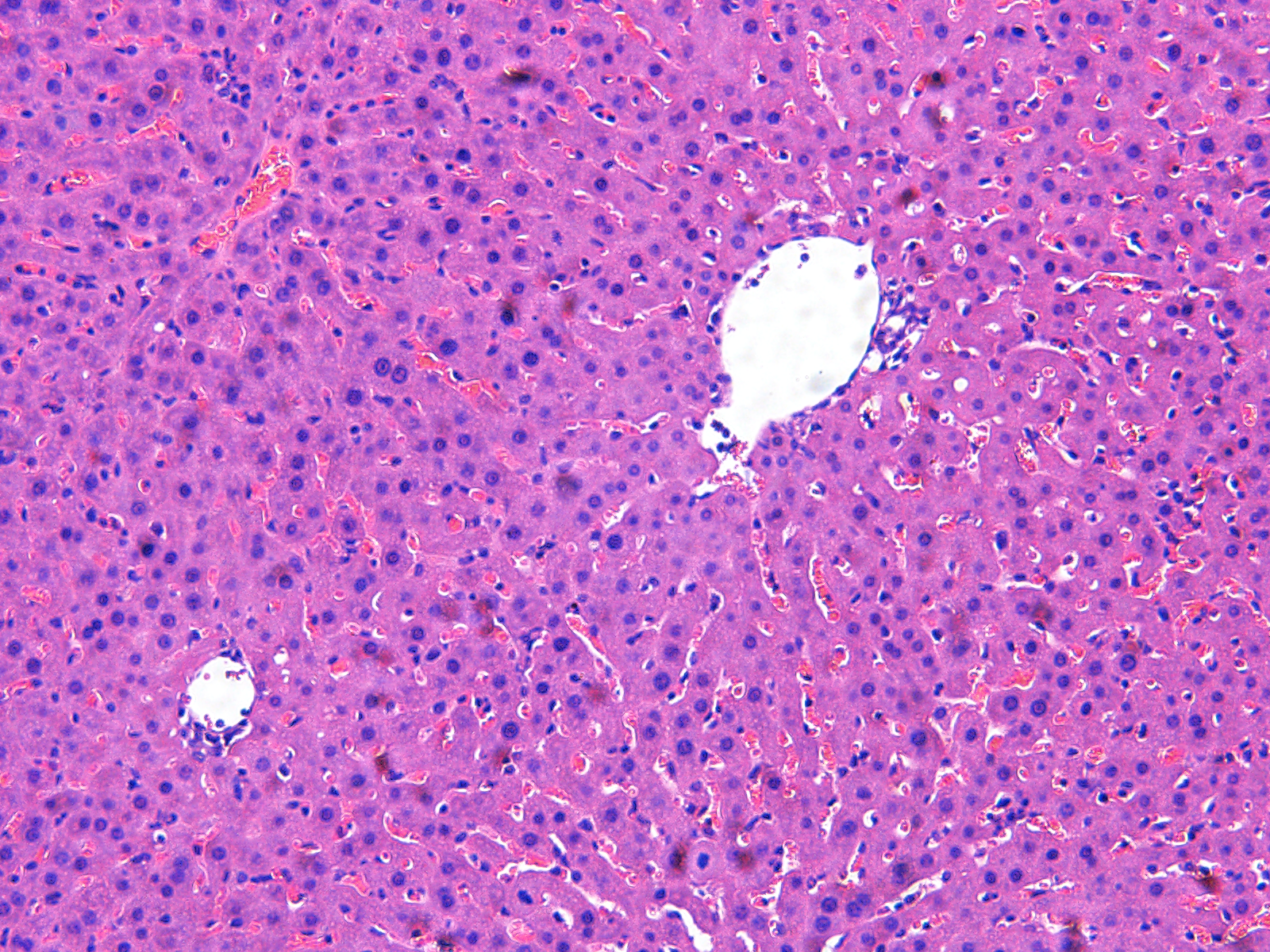

Supplement: Supplementary file 9 — Source data Fig. 7 [file 44318_2025_362_MOESM9_ESM.zip › Figure 7/7D/HE/GFP+Lova-20x.tif]

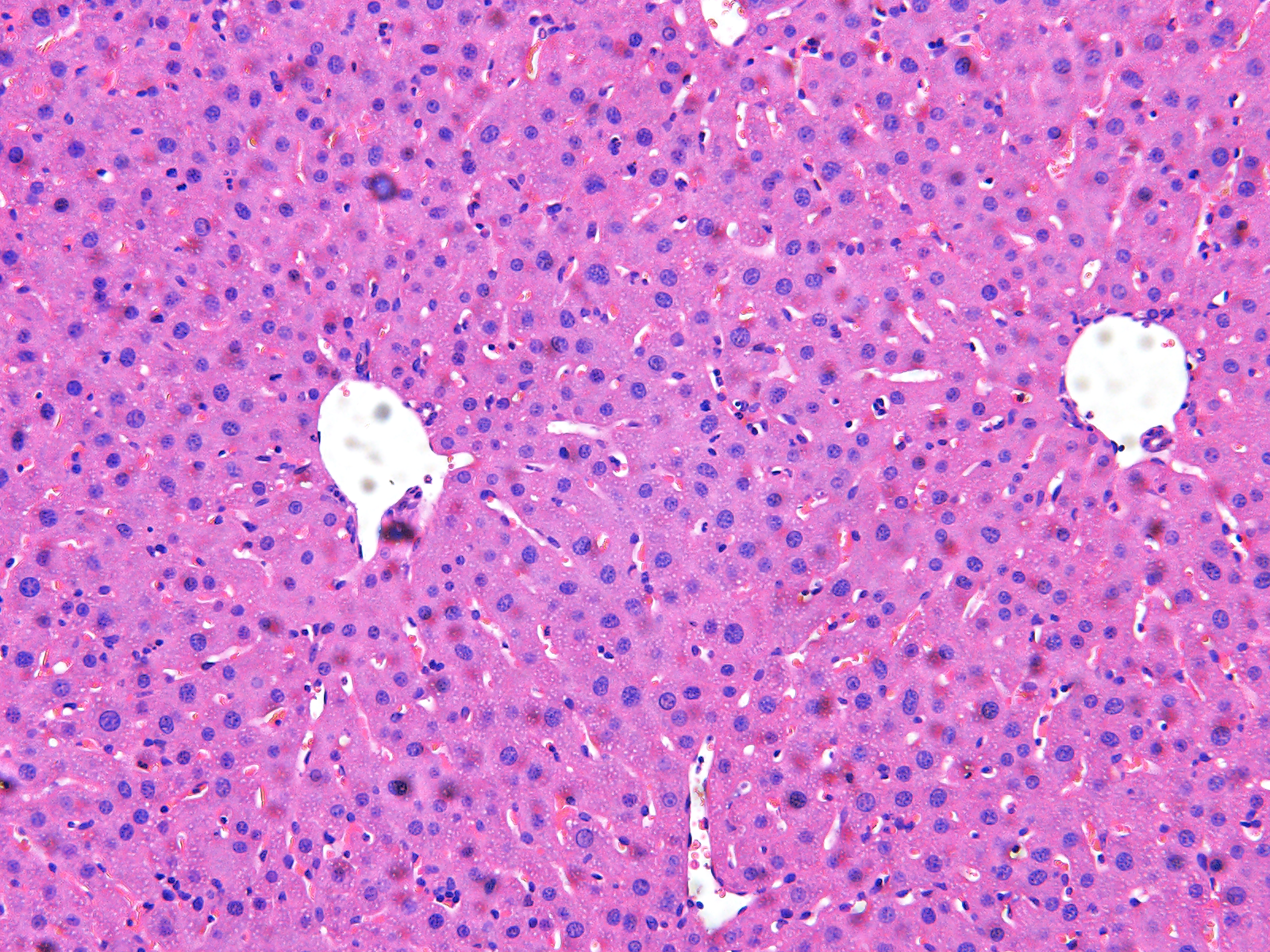

Supplement: Supplementary file 9 — Source data Fig. 7 [file 44318_2025_362_MOESM9_ESM.zip › Figure 7/7D/HE/GFP-20x.tif]

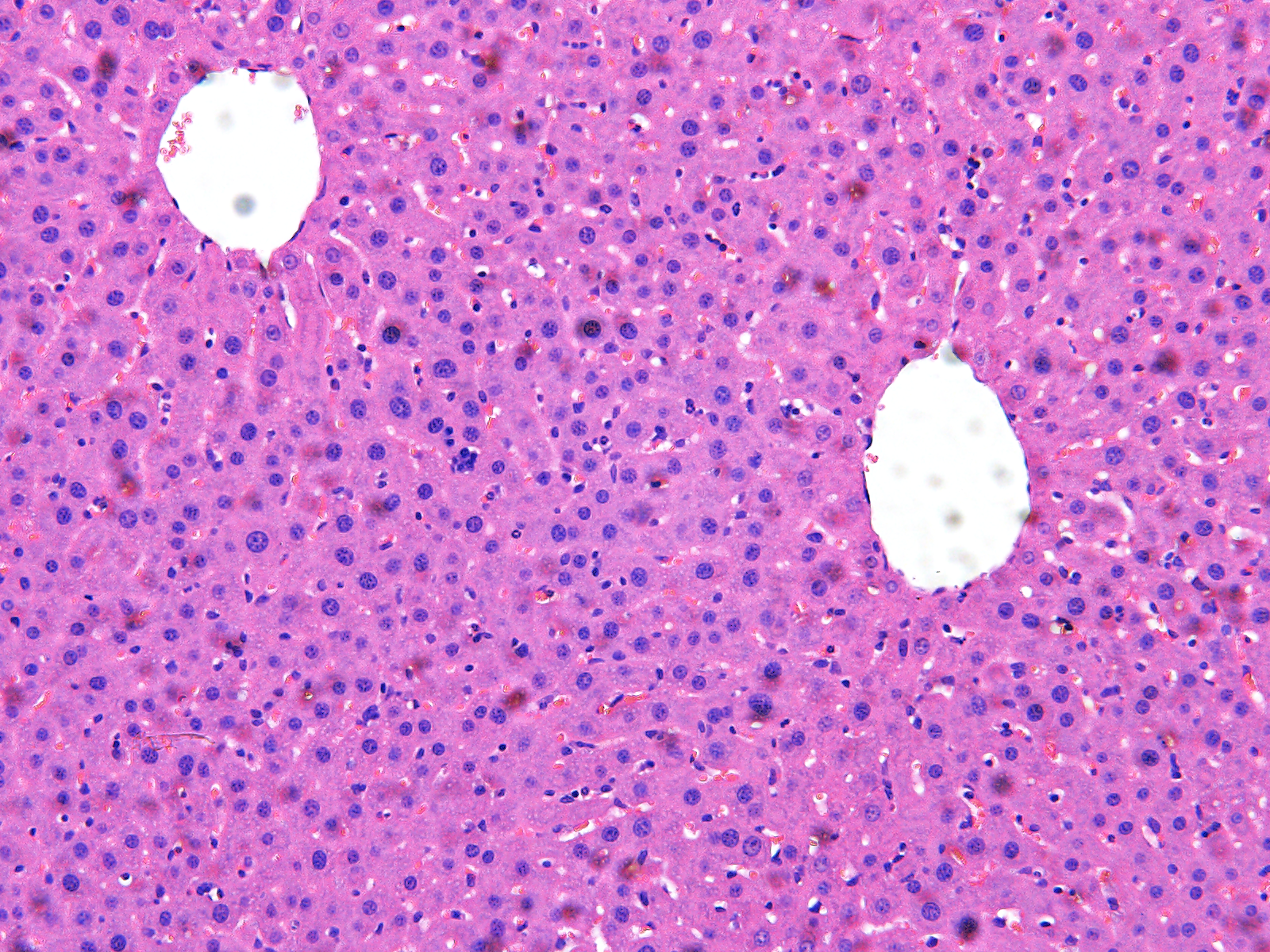

Supplement: Supplementary file 9 — Source data Fig. 7 [file 44318_2025_362_MOESM9_ESM.zip › Figure 7/7D/HE/SLC-20x.tif]

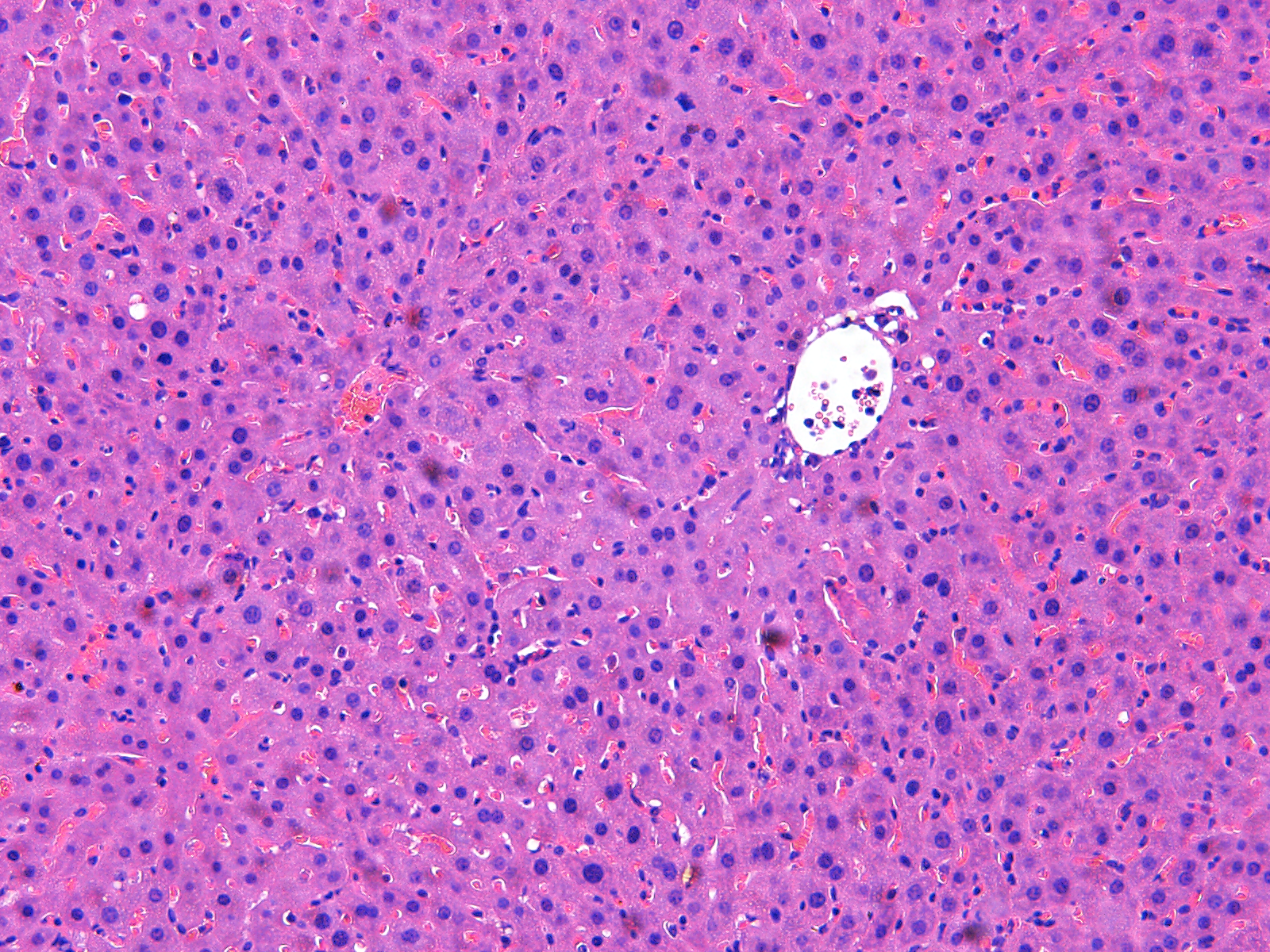

Supplement: Supplementary file 9 — Source data Fig. 7 [file 44318_2025_362_MOESM9_ESM.zip › Figure 7/7D/HE/SLC+Lova-20x.tif]

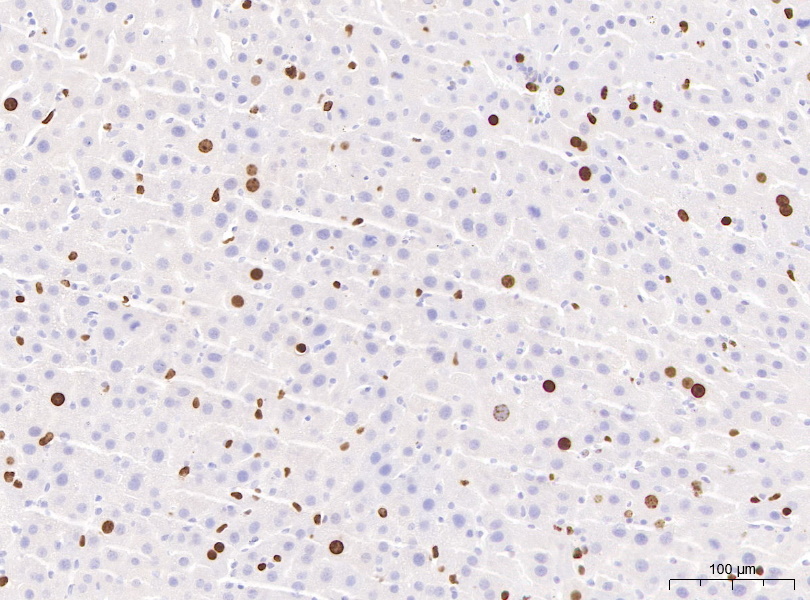

Supplement: Supplementary file 9 — Source data Fig. 7 [file 44318_2025_362_MOESM9_ESM.zip › Figure 7/7D/BrdU/GFP-20x.jpg]

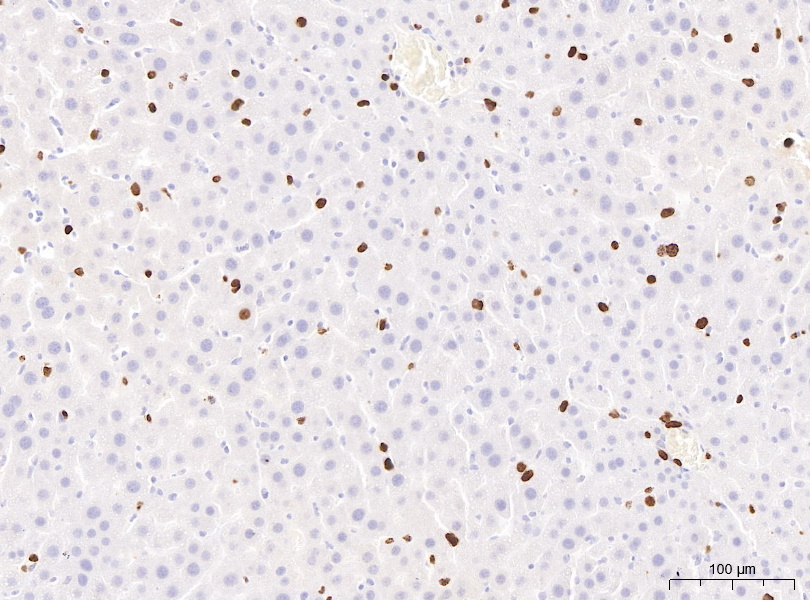

Supplement: Supplementary file 9 — Source data Fig. 7 [file 44318_2025_362_MOESM9_ESM.zip › Figure 7/7D/BrdU/GFP+Lova-20x.jpg]

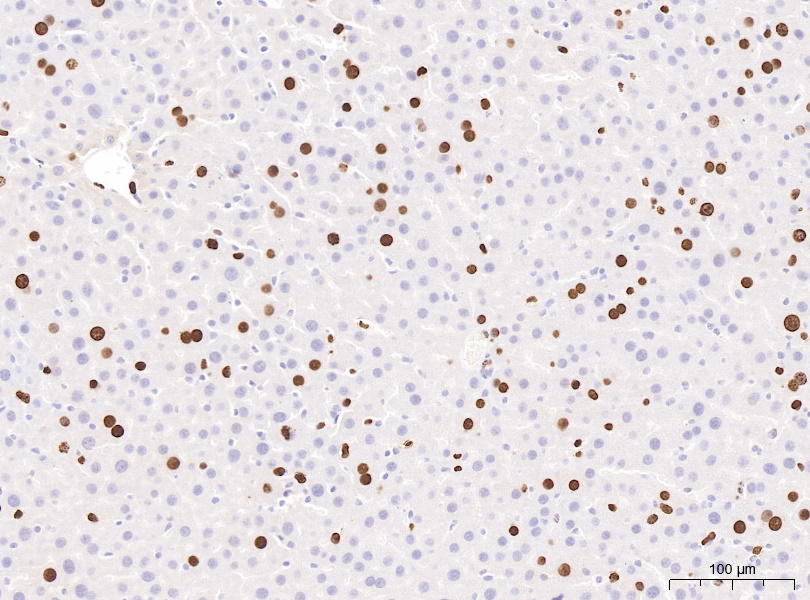

Supplement: Supplementary file 9 — Source data Fig. 7 [file 44318_2025_362_MOESM9_ESM.zip › Figure 7/7D/BrdU/SLC-20x.jpg]

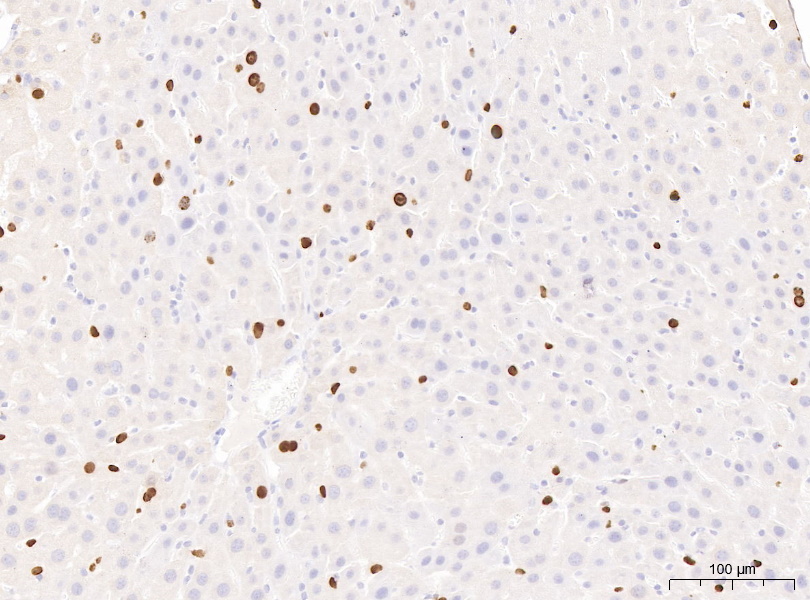

Supplement: Supplementary file 9 — Source data Fig. 7 [file 44318_2025_362_MOESM9_ESM.zip › Figure 7/7D/BrdU/SLC+Lova-20x.jpg]

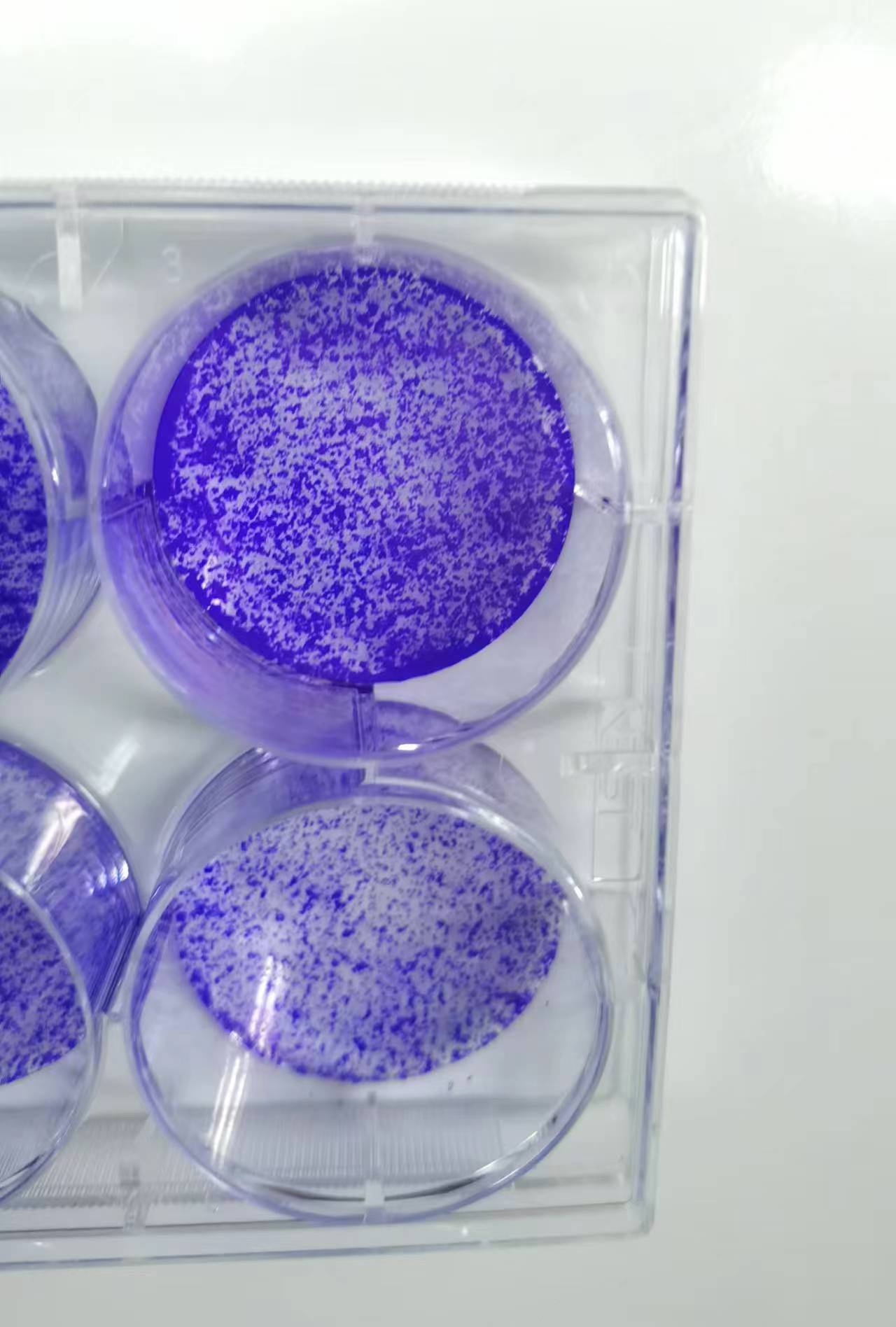

Supplement: Supplementary file 10 — Source data Fig. 8 [file 44318_2025_362_MOESM10_ESM.zip › Figure 8/8A/Colony/SLC13A2.jpg]

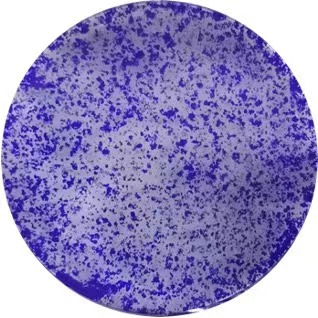

Supplement: Supplementary file 10 — Source data Fig. 8 [file 44318_2025_362_MOESM10_ESM.zip › Figure 8/8A/Colony/Vector-cut.jpg]

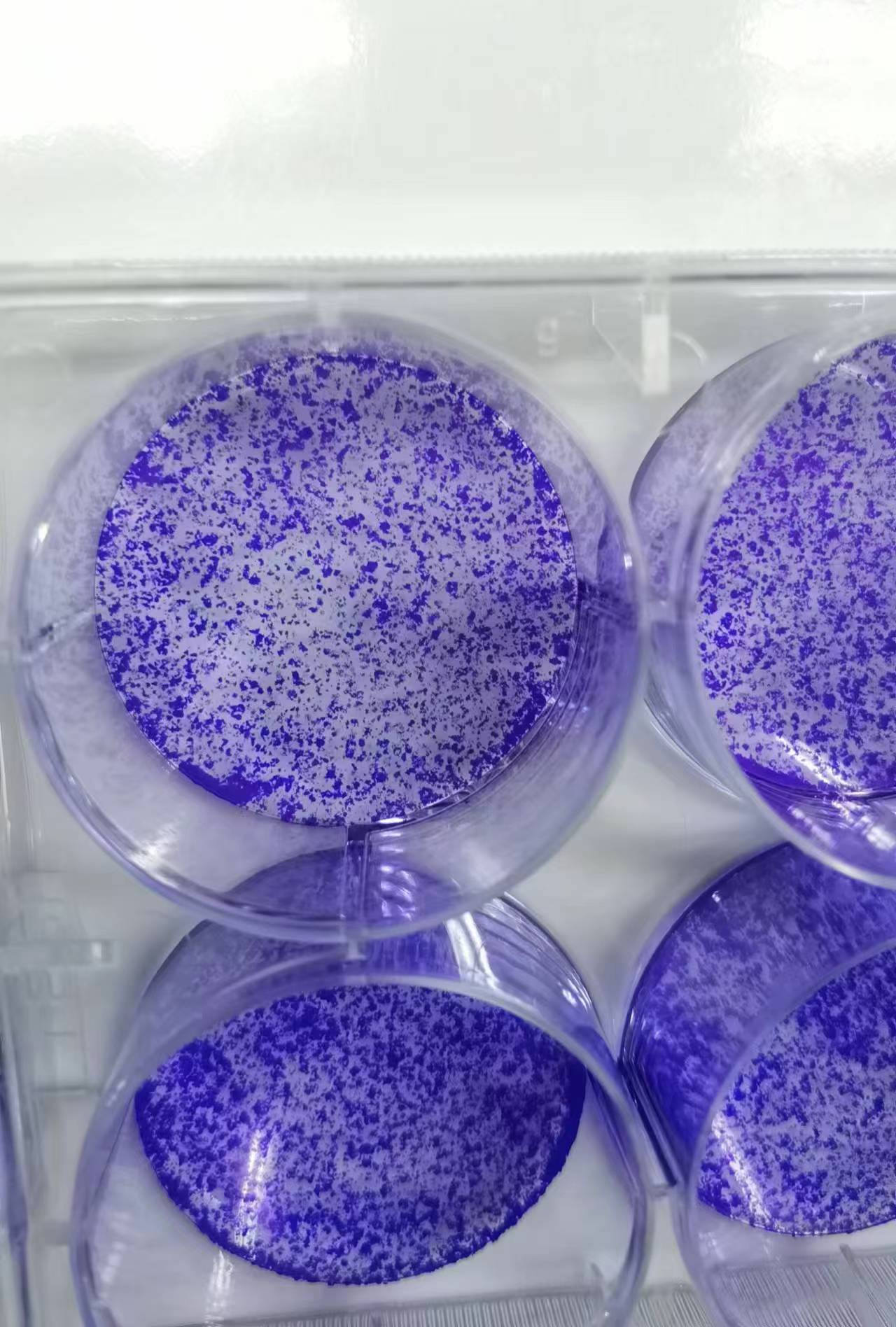

Supplement: Supplementary file 10 — Source data Fig. 8 [file 44318_2025_362_MOESM10_ESM.zip › Figure 8/8A/Colony/Vector.jpg]

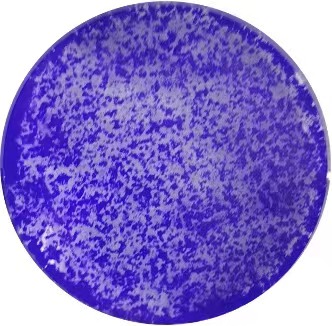

Supplement: Supplementary file 10 — Source data Fig. 8 [file 44318_2025_362_MOESM10_ESM.zip › Figure 8/8A/Colony/SLC13A2-cut.jpg]

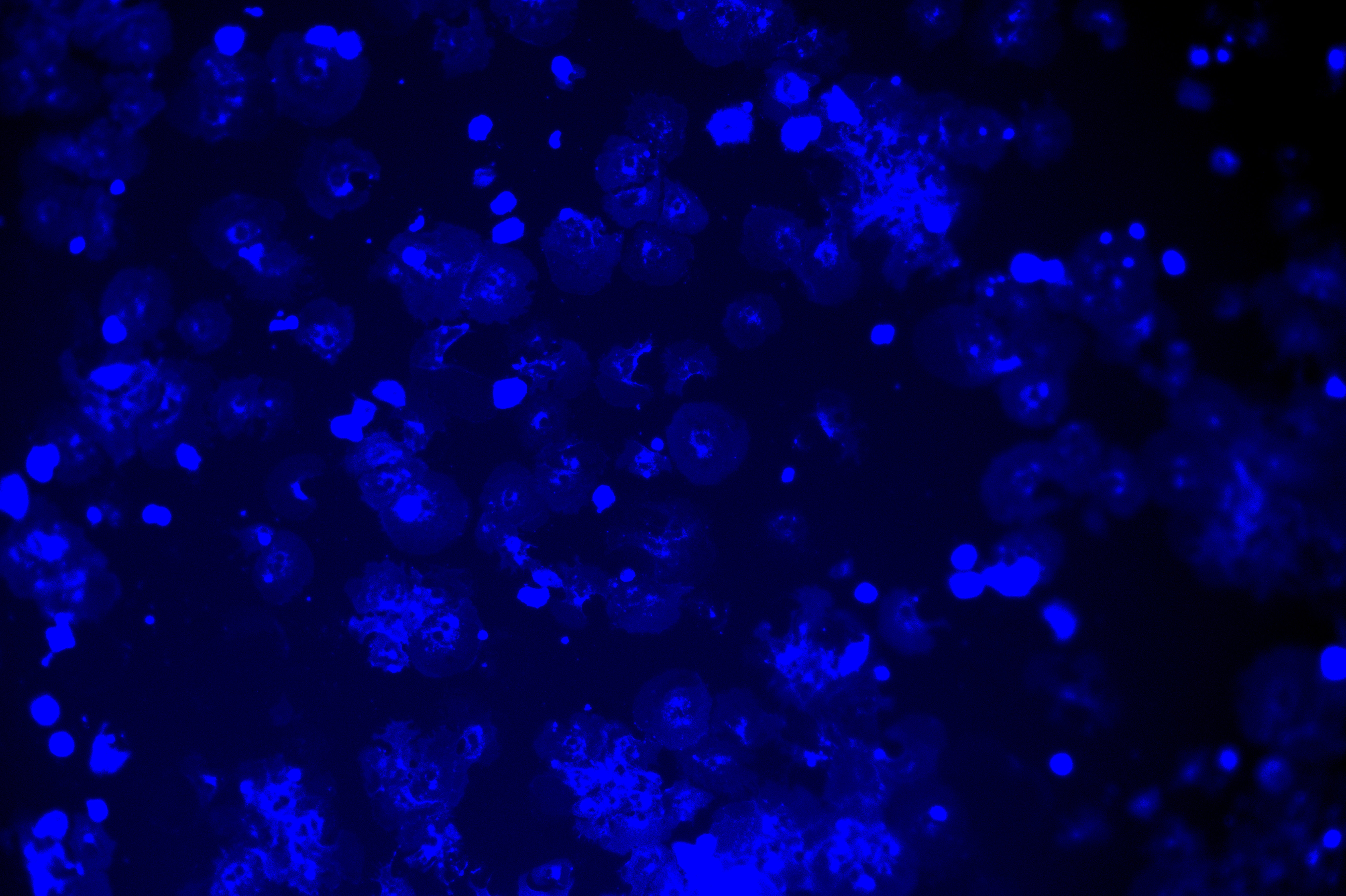

Supplement: Supplementary file 10 — Source data Fig. 8 [file 44318_2025_362_MOESM10_ESM.zip › Figure 8/8B/MPH-Cholesterol/SLC.jpg]

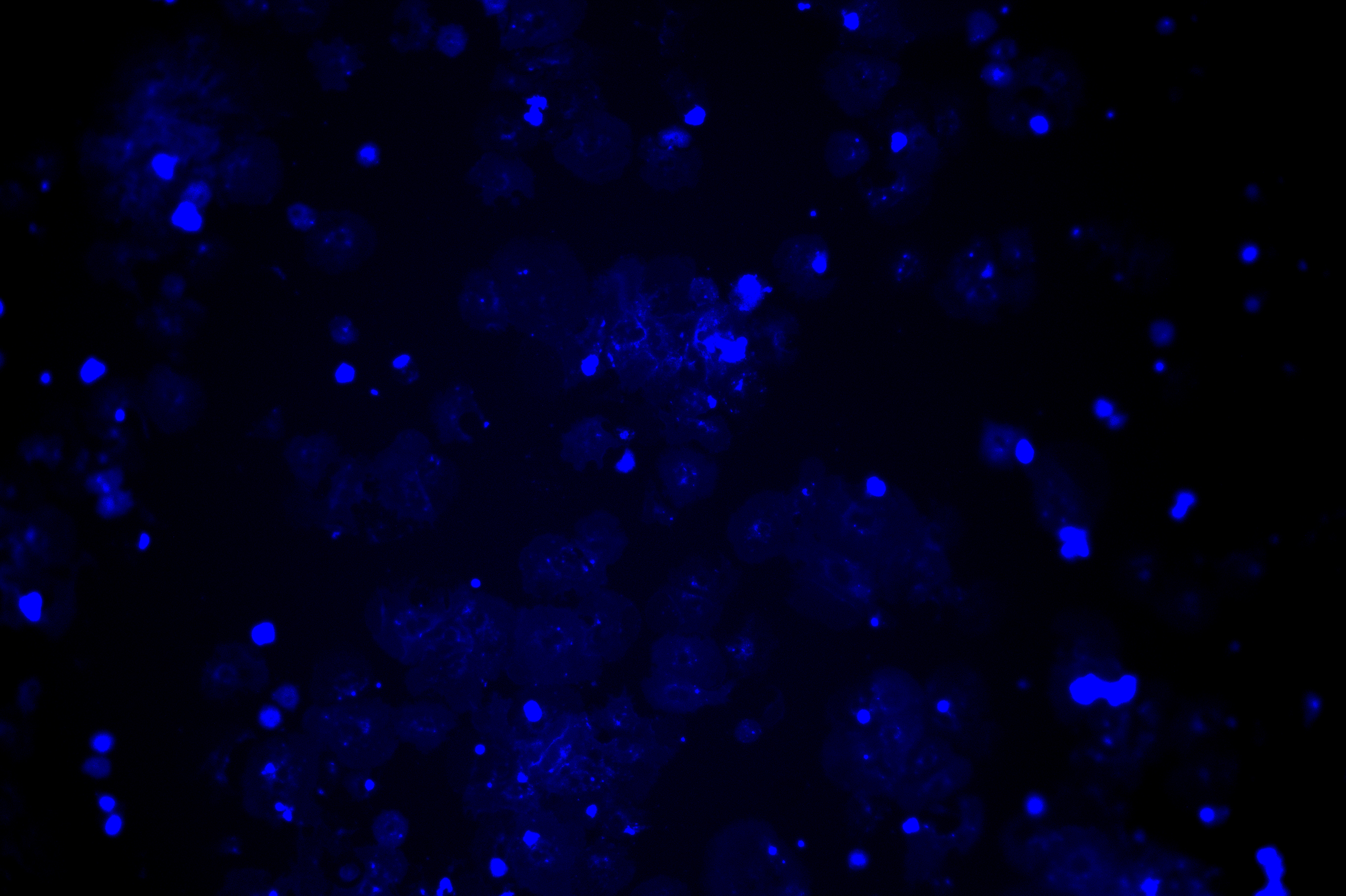

Supplement: Supplementary file 10 — Source data Fig. 8 [file 44318_2025_362_MOESM10_ESM.zip › Figure 8/8B/MPH-Cholesterol/Vector.jpg]

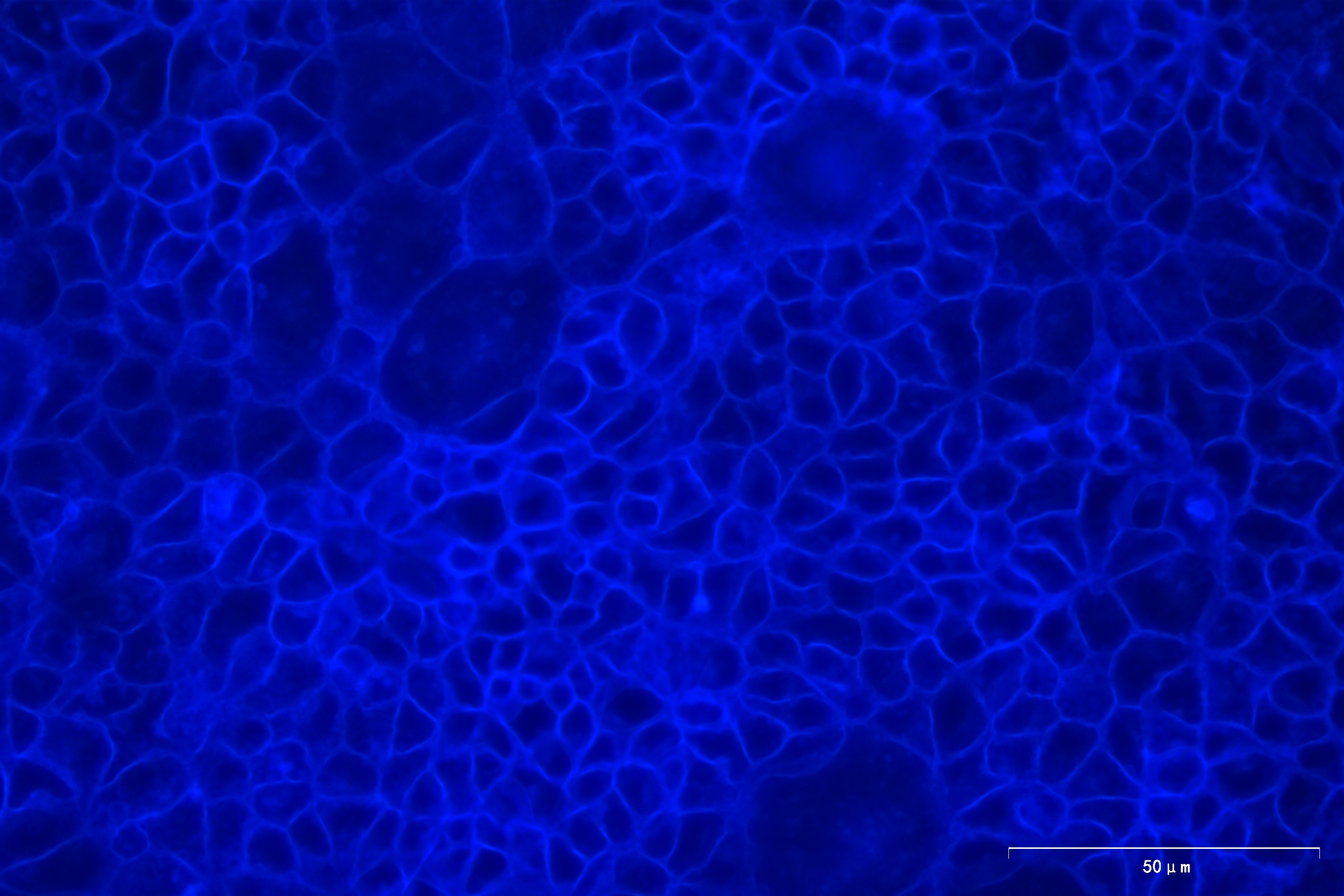

Supplement: Supplementary file 10 — Source data Fig. 8 [file 44318_2025_362_MOESM10_ESM.zip › Figure 8/8B/AML12-Cholesterol/SLC.jpg]

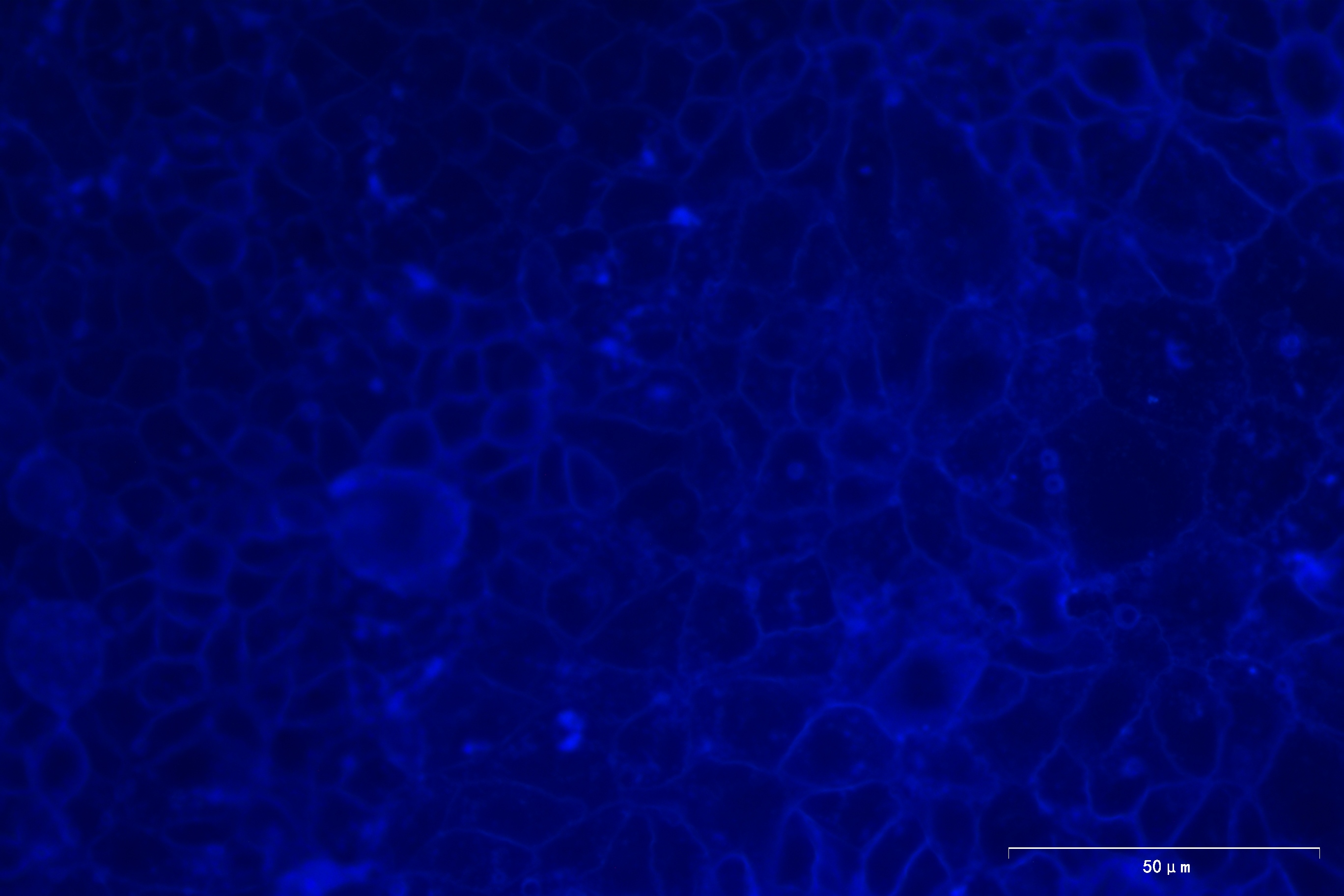

Supplement: Supplementary file 10 — Source data Fig. 8 [file 44318_2025_362_MOESM10_ESM.zip › Figure 8/8B/AML12-Cholesterol/Vector.jpg]

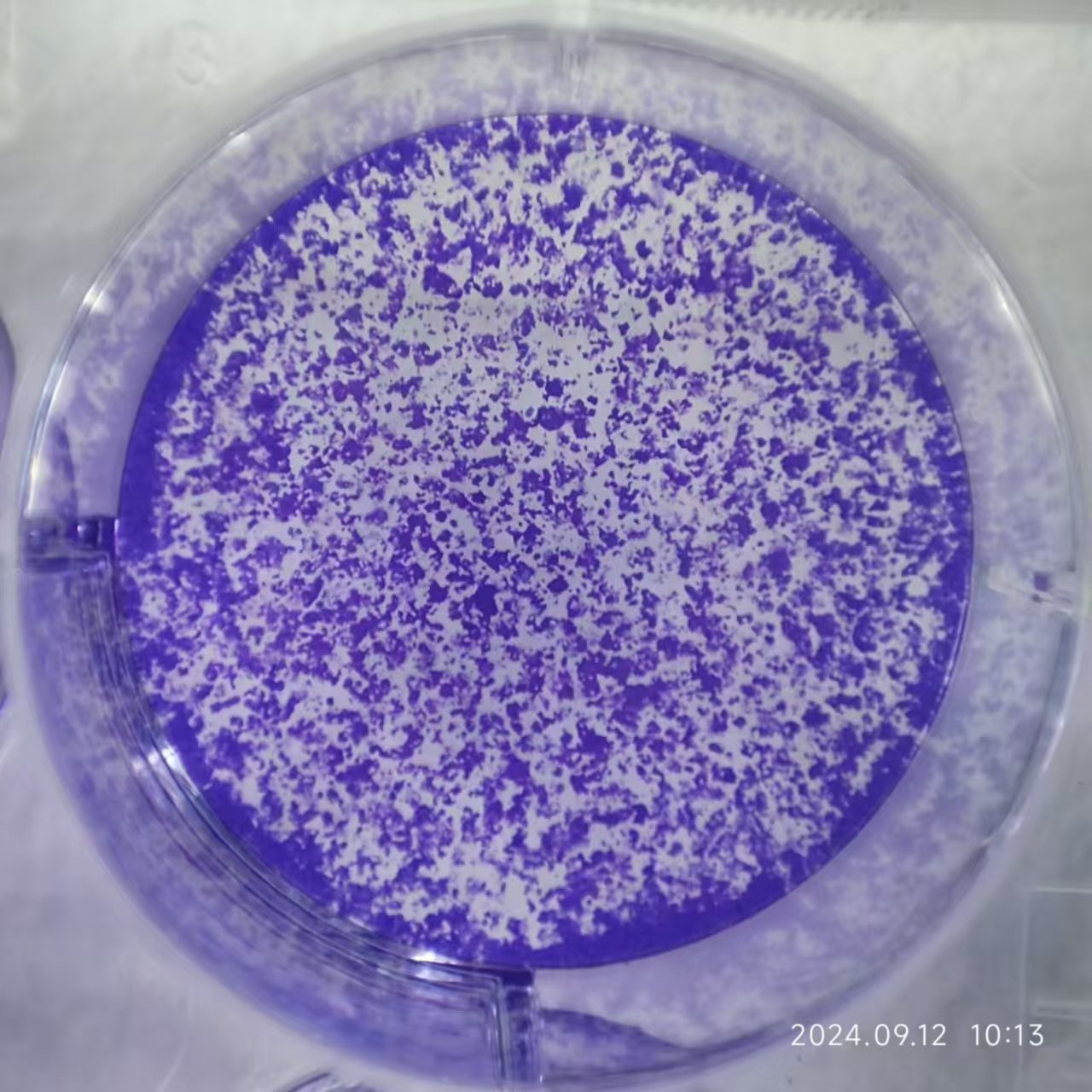

Supplement: Supplementary file 10 — Source data Fig. 8 [file 44318_2025_362_MOESM10_ESM.zip › Figure 8/8A/Colony/Replicate/SLC13A2/SLC-1.jpg]

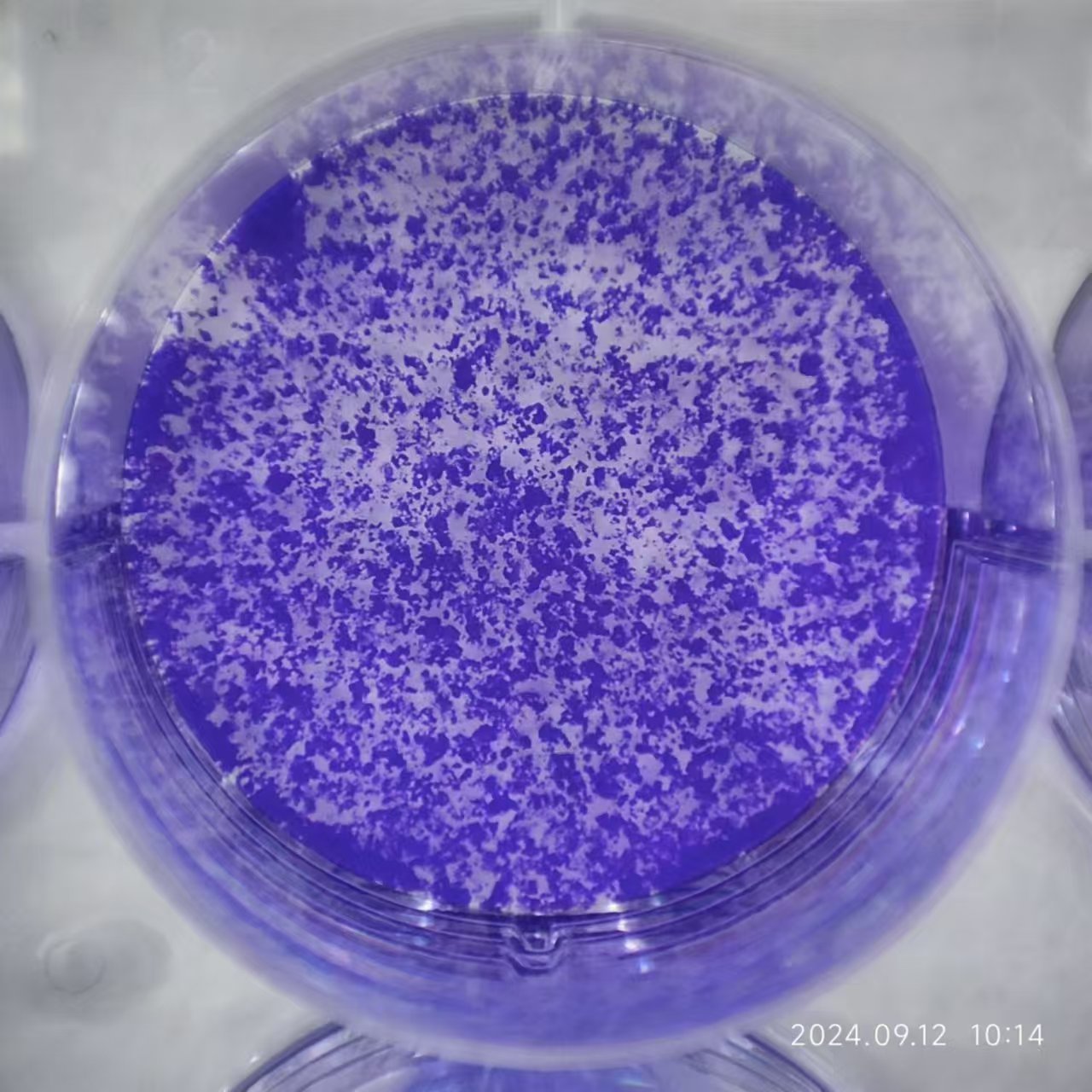

Supplement: Supplementary file 10 — Source data Fig. 8 [file 44318_2025_362_MOESM10_ESM.zip › Figure 8/8A/Colony/Replicate/SLC13A2/SLC-2.jpg]

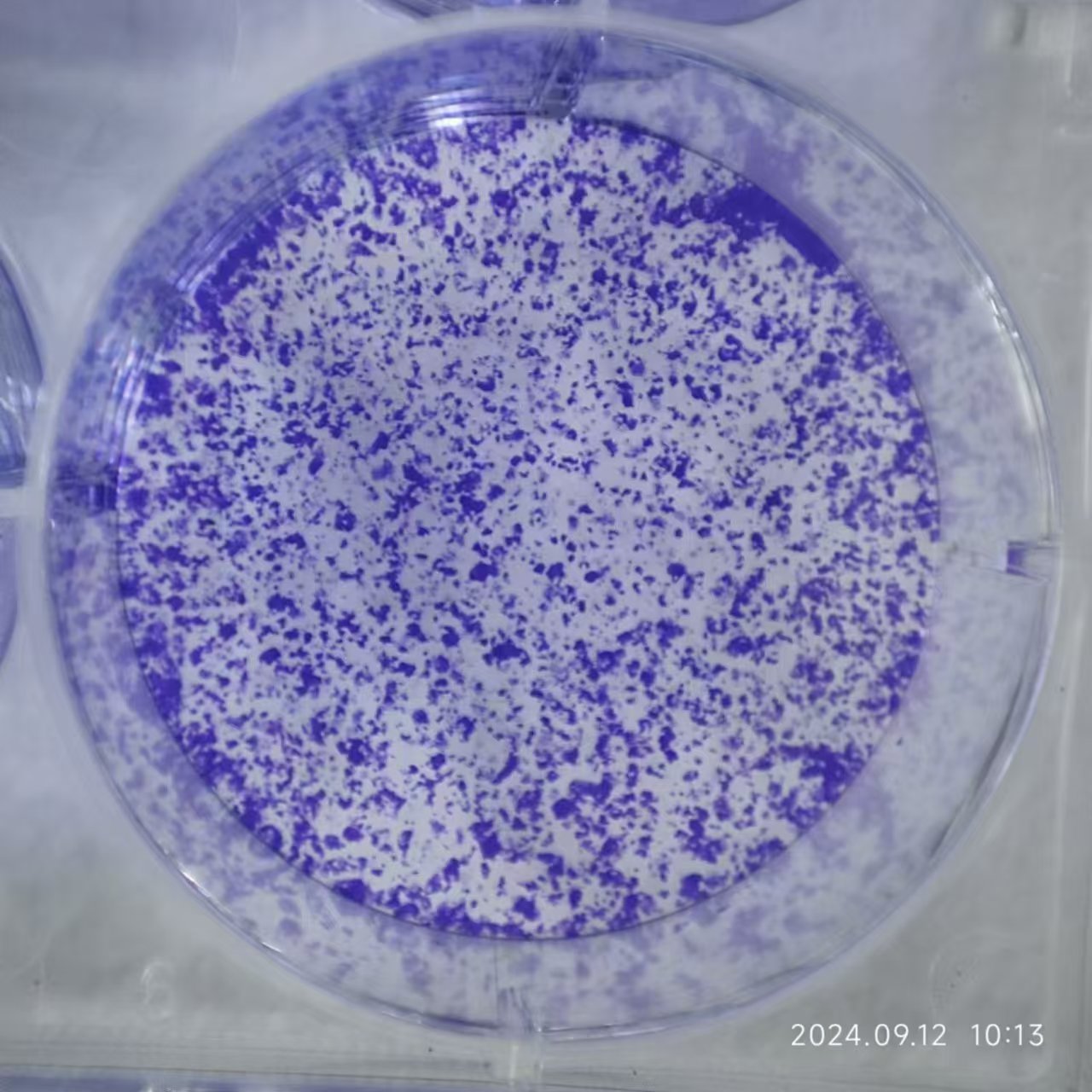

Supplement: Supplementary file 10 — Source data Fig. 8 [file 44318_2025_362_MOESM10_ESM.zip › Figure 8/8A/Colony/Replicate/Vector/Vector-1.jpg]

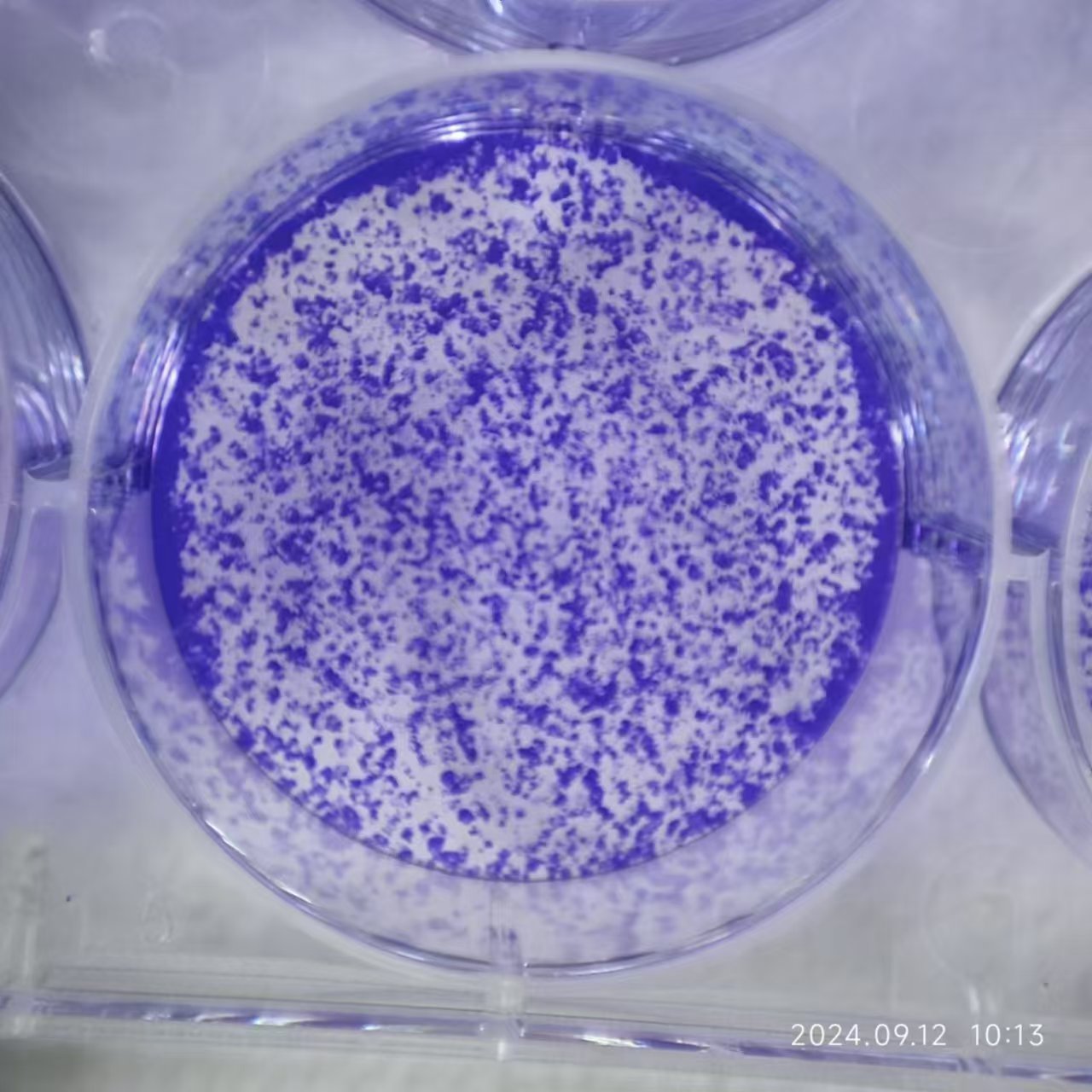

Supplement: Supplementary file 10 — Source data Fig. 8 [file 44318_2025_362_MOESM10_ESM.zip › Figure 8/8A/Colony/Replicate/Vector/Vector-2.jpg]

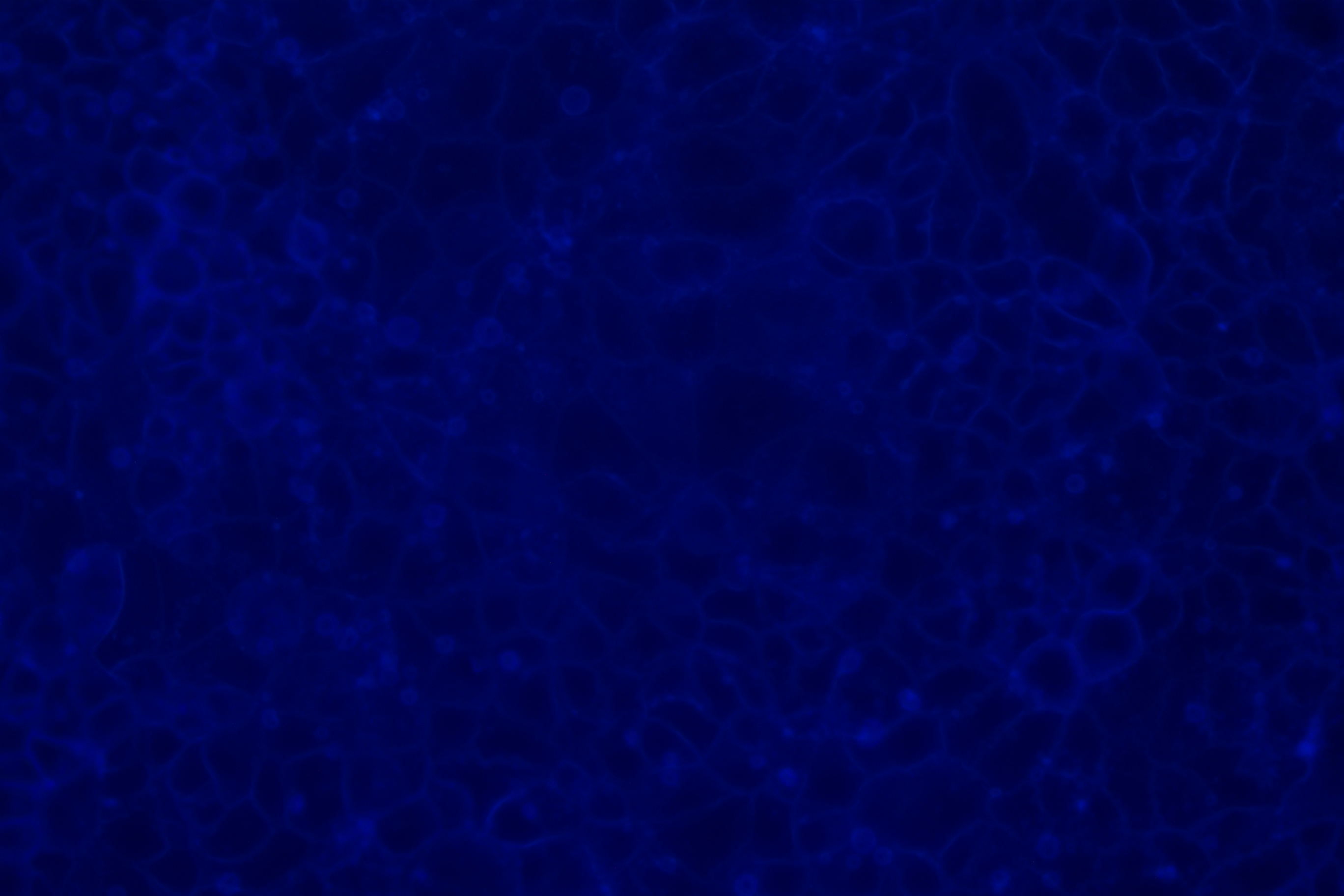

Supplement: Supplementary file 11 — Source data Fig. 9 [file 44318_2025_362_MOESM11_ESM.zip › Figure 9/9J/Vector+ACLYi/Cholesterol.jpg]

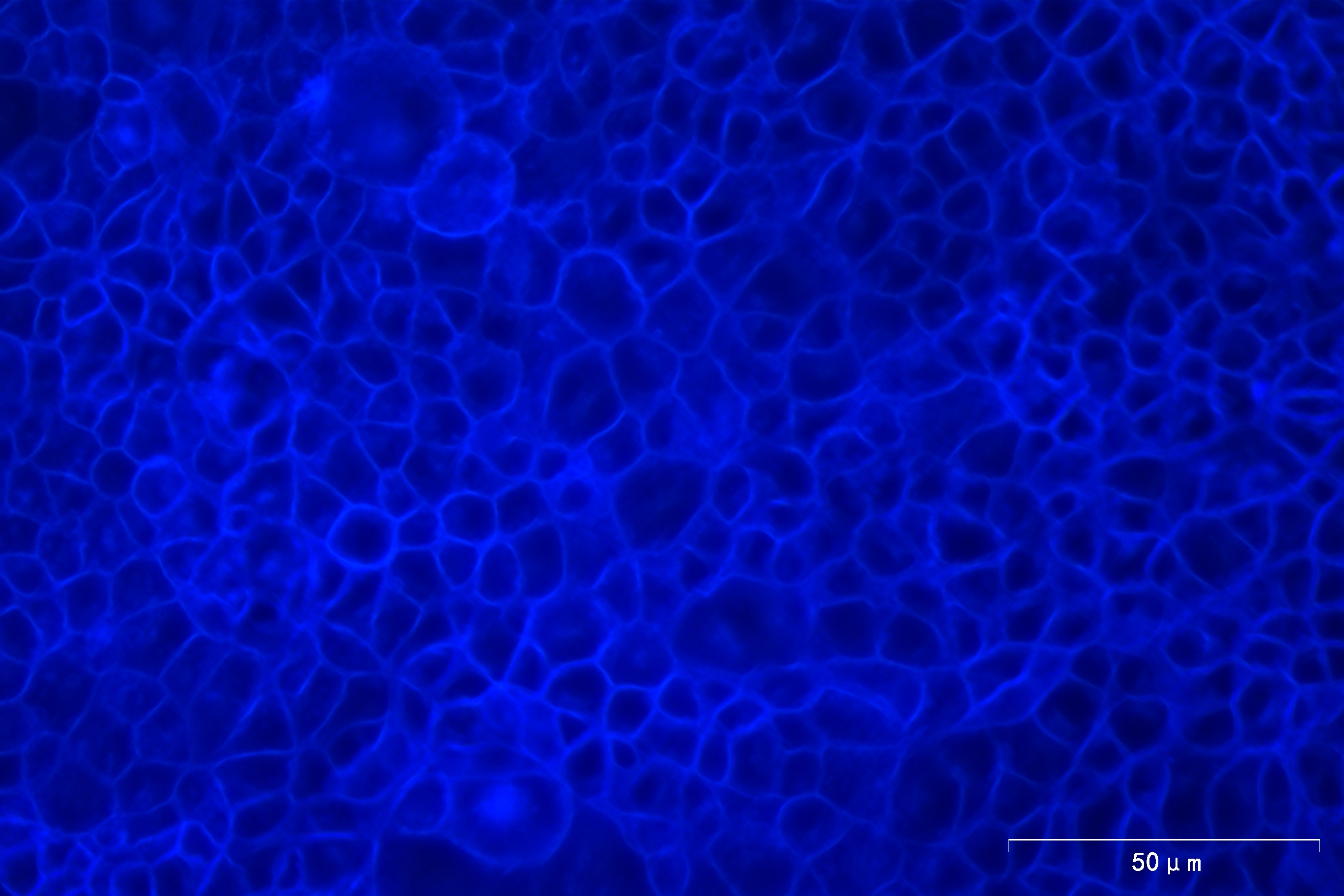

Supplement: Supplementary file 11 — Source data Fig. 9 [file 44318_2025_362_MOESM11_ESM.zip › Figure 9/9J/SLC13A2+Vehicle/Cholesterol.jpg]

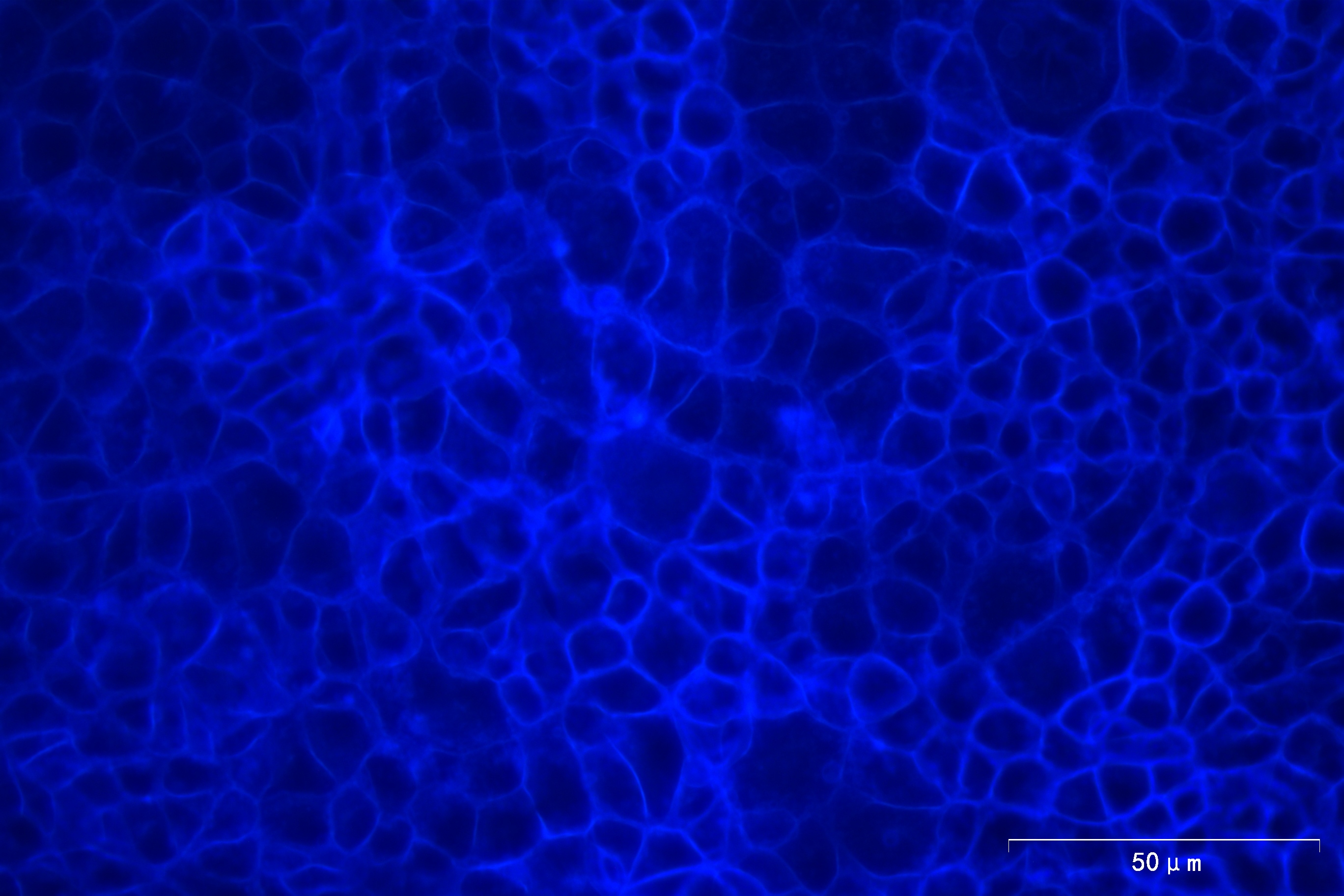

Supplement: Supplementary file 11 — Source data Fig. 9 [file 44318_2025_362_MOESM11_ESM.zip › Figure 9/9J/SLC13A2+ACLYi+Cholesterol/Cholesterol.jpg]

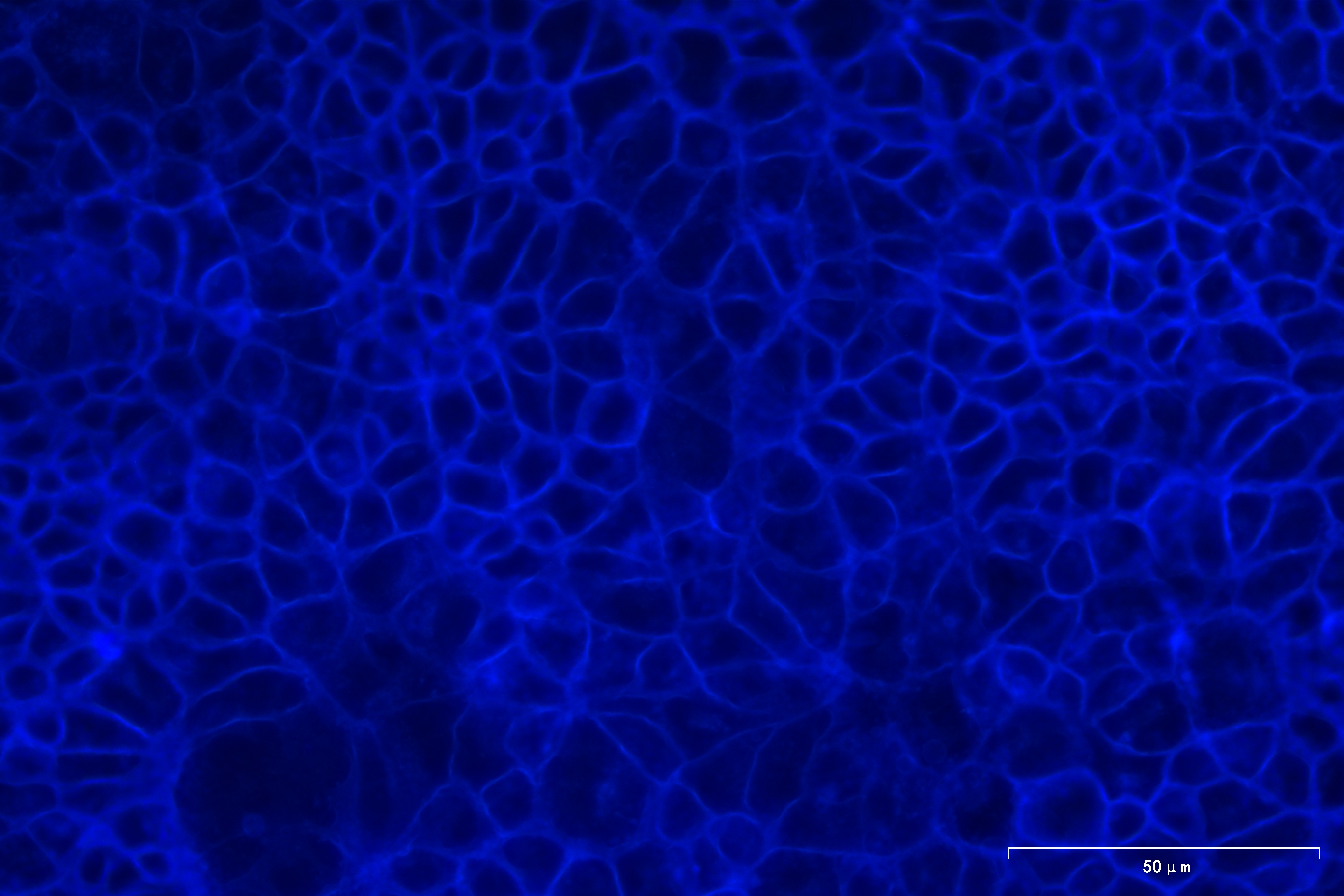

Supplement: Supplementary file 11 — Source data Fig. 9 [file 44318_2025_362_MOESM11_ESM.zip › Figure 9/9J/SLC13A2+ACLYi/Cholesterol.jpg]

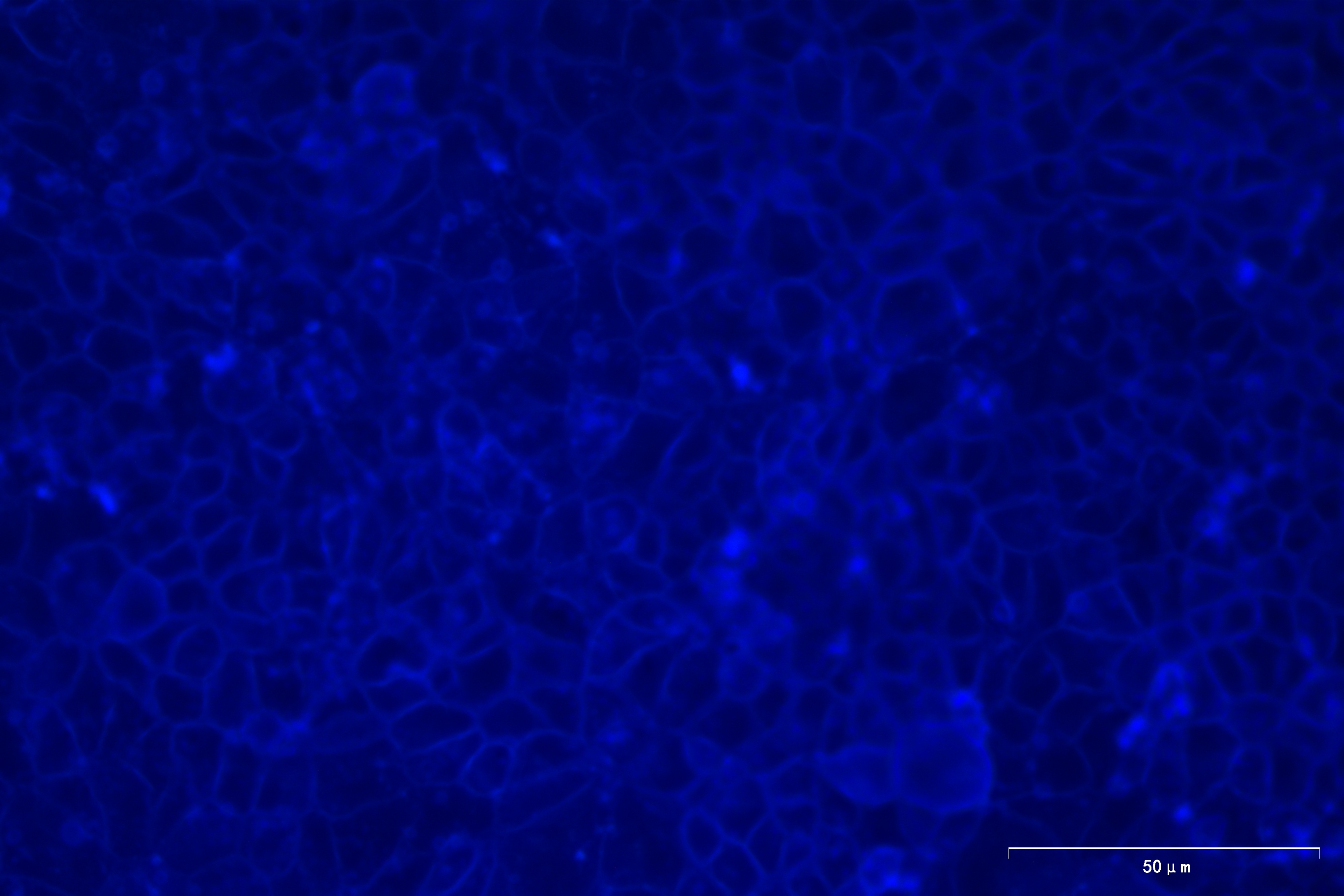

Supplement: Supplementary file 11 — Source data Fig. 9 [file 44318_2025_362_MOESM11_ESM.zip › Figure 9/9J/Vector+Vehicle/Cholesterol.jpg]

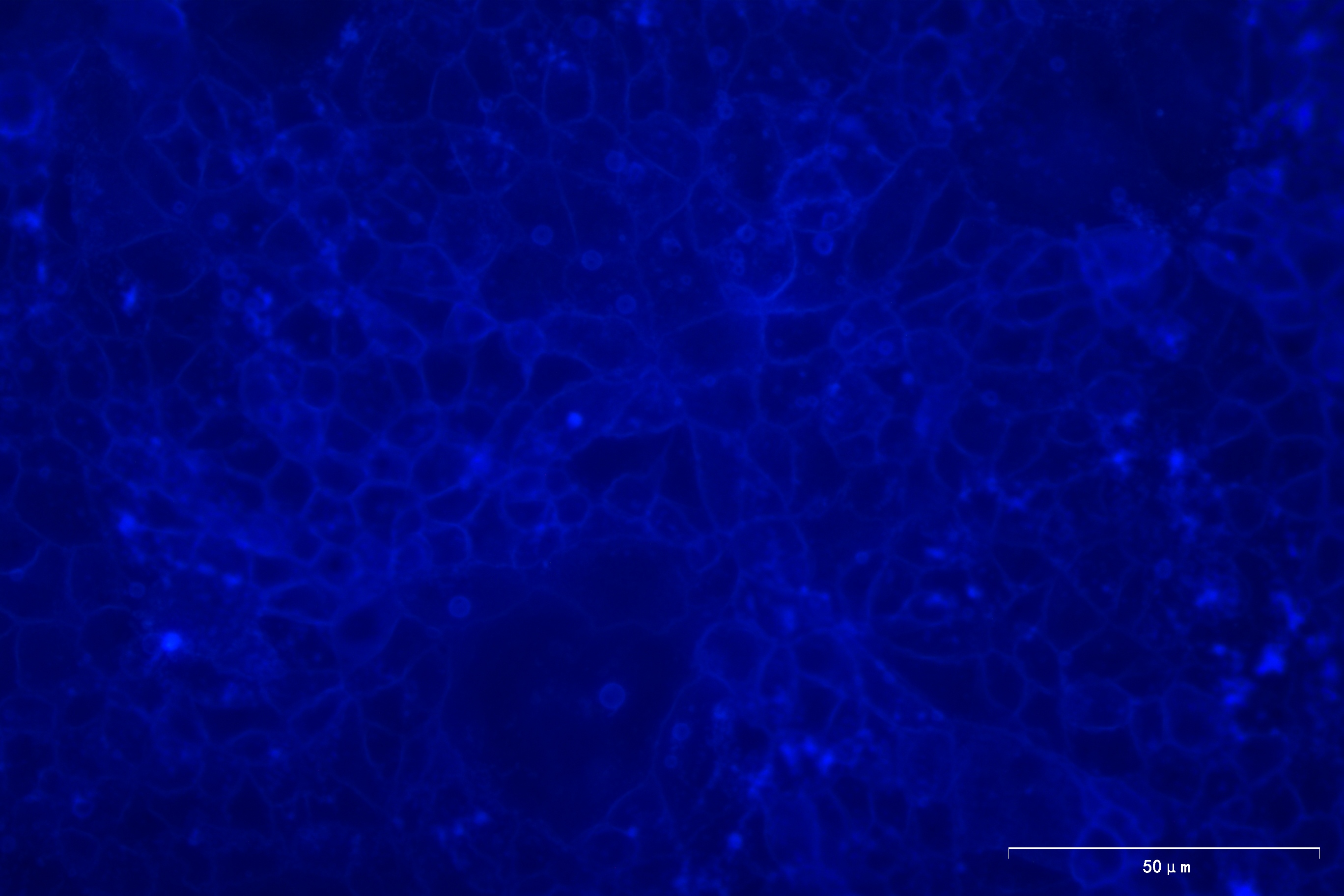

Supplement: Supplementary file 11 — Source data Fig. 9 [file 44318_2025_362_MOESM11_ESM.zip › Figure 9/9J/Vector+ACLYi+Cholesterol/Cholesterol.jpg]

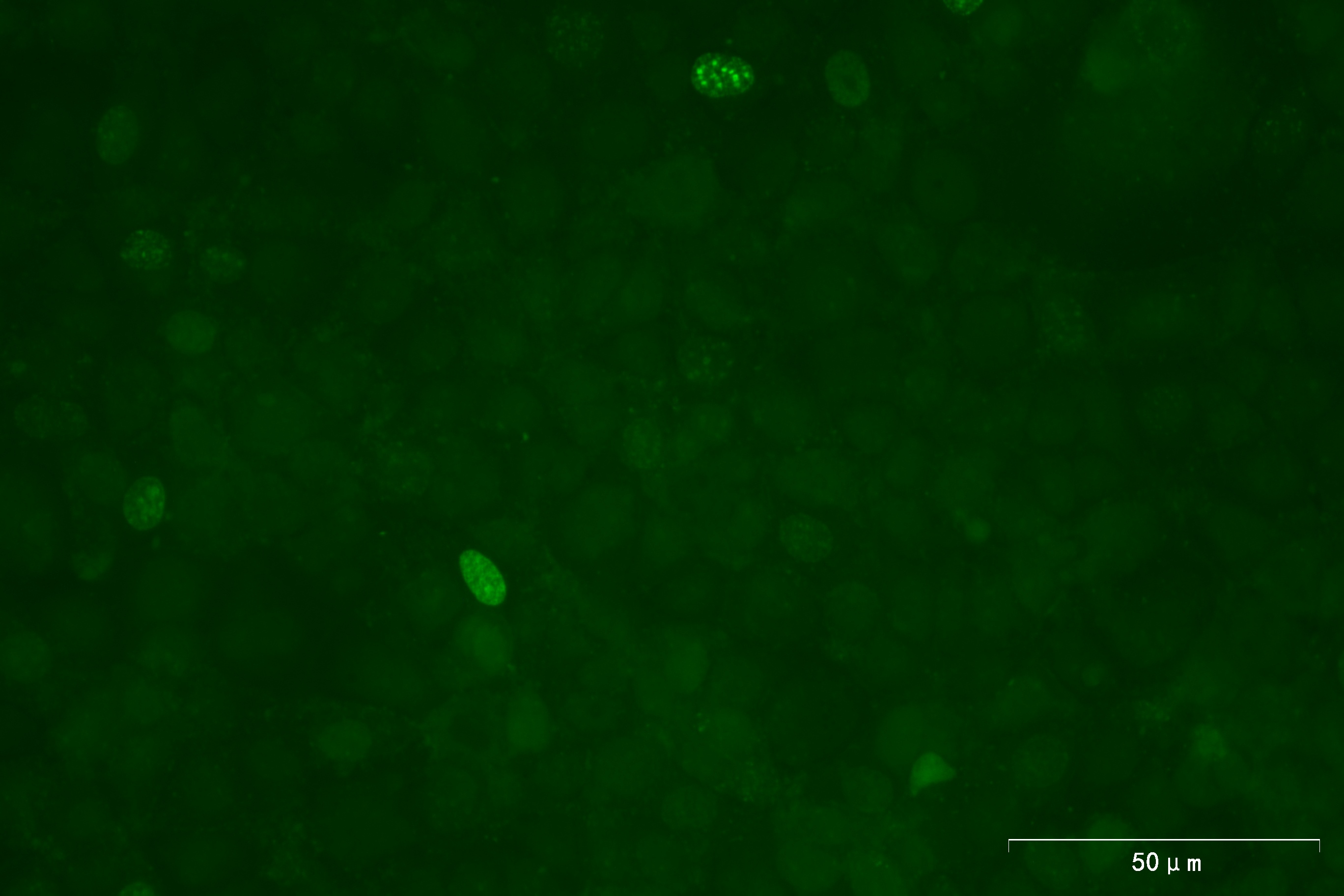

Supplement: Supplementary file 11 — Source data Fig. 9 [file 44318_2025_362_MOESM11_ESM.zip › Figure 9/9I/Vector+ACLYi/EdU.jpg]

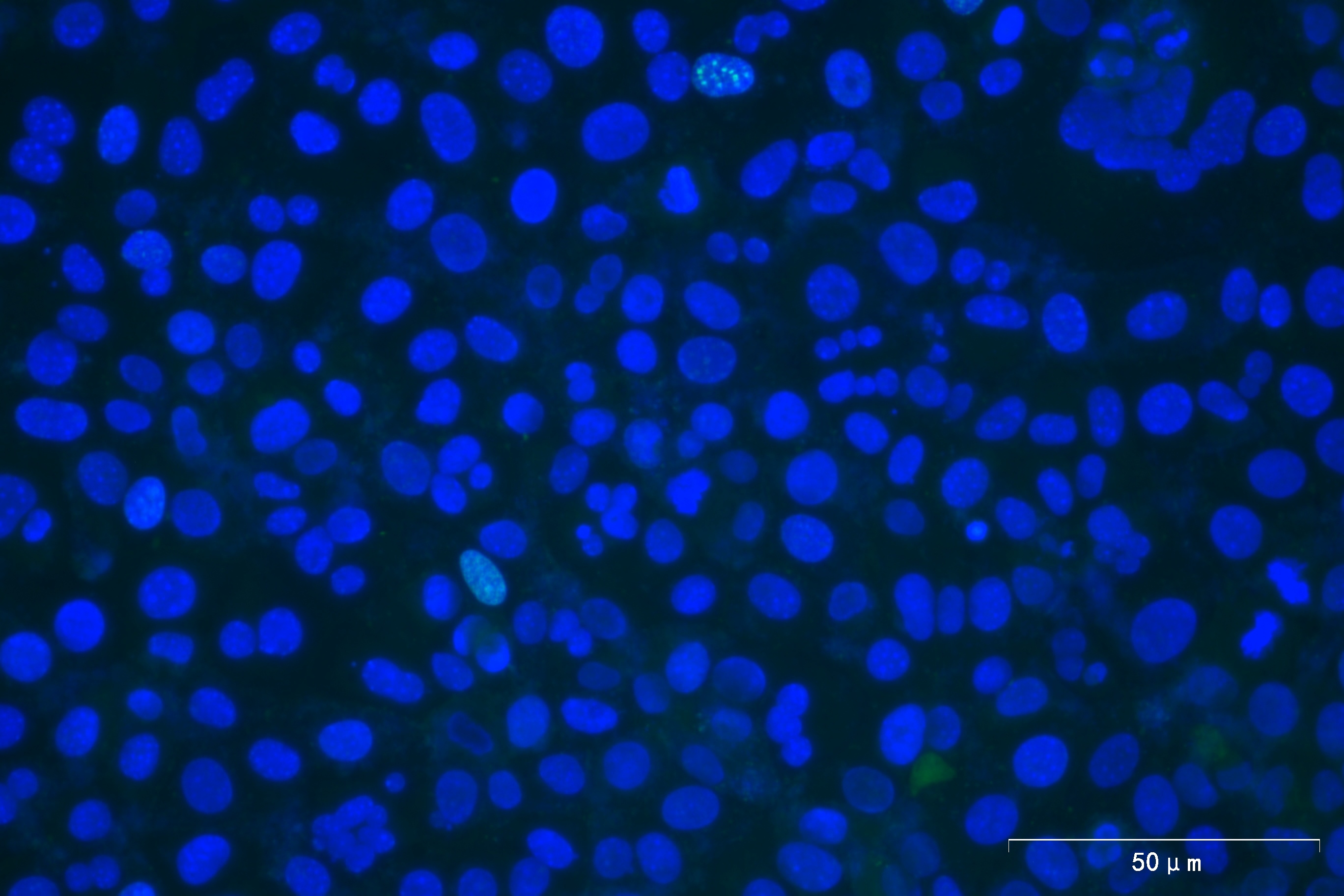

Supplement: Supplementary file 11 — Source data Fig. 9 [file 44318_2025_362_MOESM11_ESM.zip › Figure 9/9I/Vector+ACLYi/Merge.jpg]

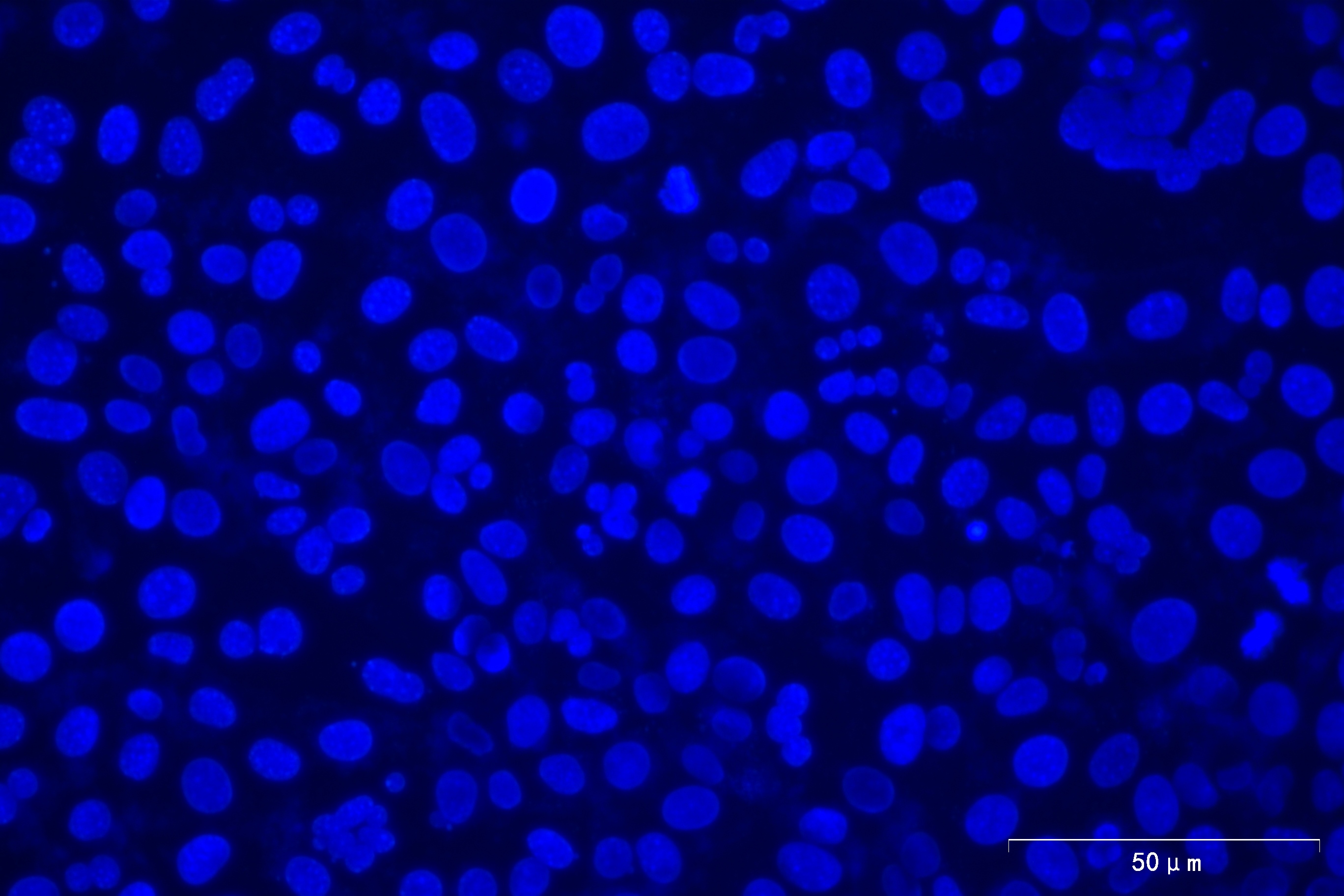

Supplement: Supplementary file 11 — Source data Fig. 9 [file 44318_2025_362_MOESM11_ESM.zip › Figure 9/9I/Vector+ACLYi/DAPI.jpg]

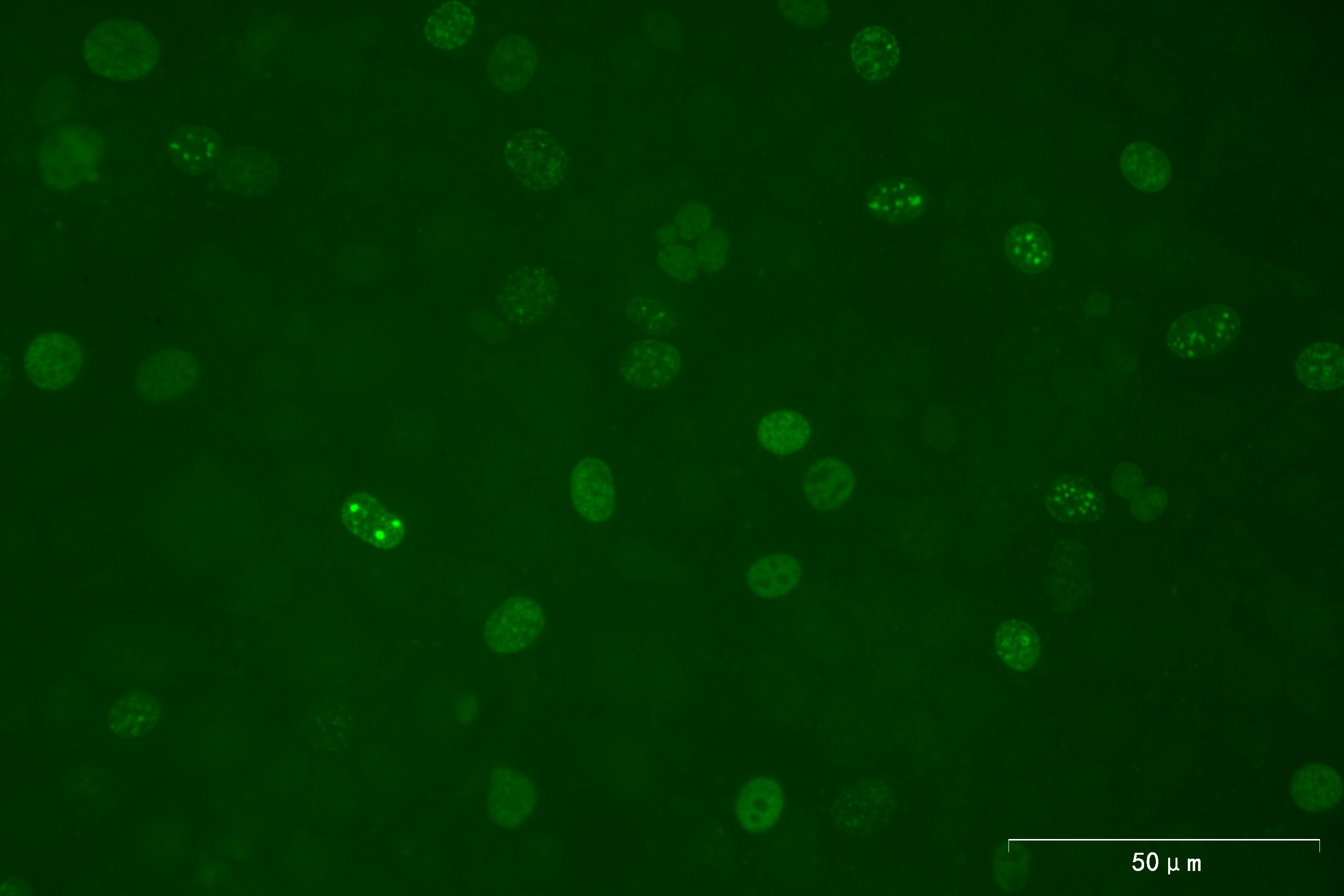

Supplement: Supplementary file 11 — Source data Fig. 9 [file 44318_2025_362_MOESM11_ESM.zip › Figure 9/9I/SLC13A2+Vehicle/EdU.jpg]

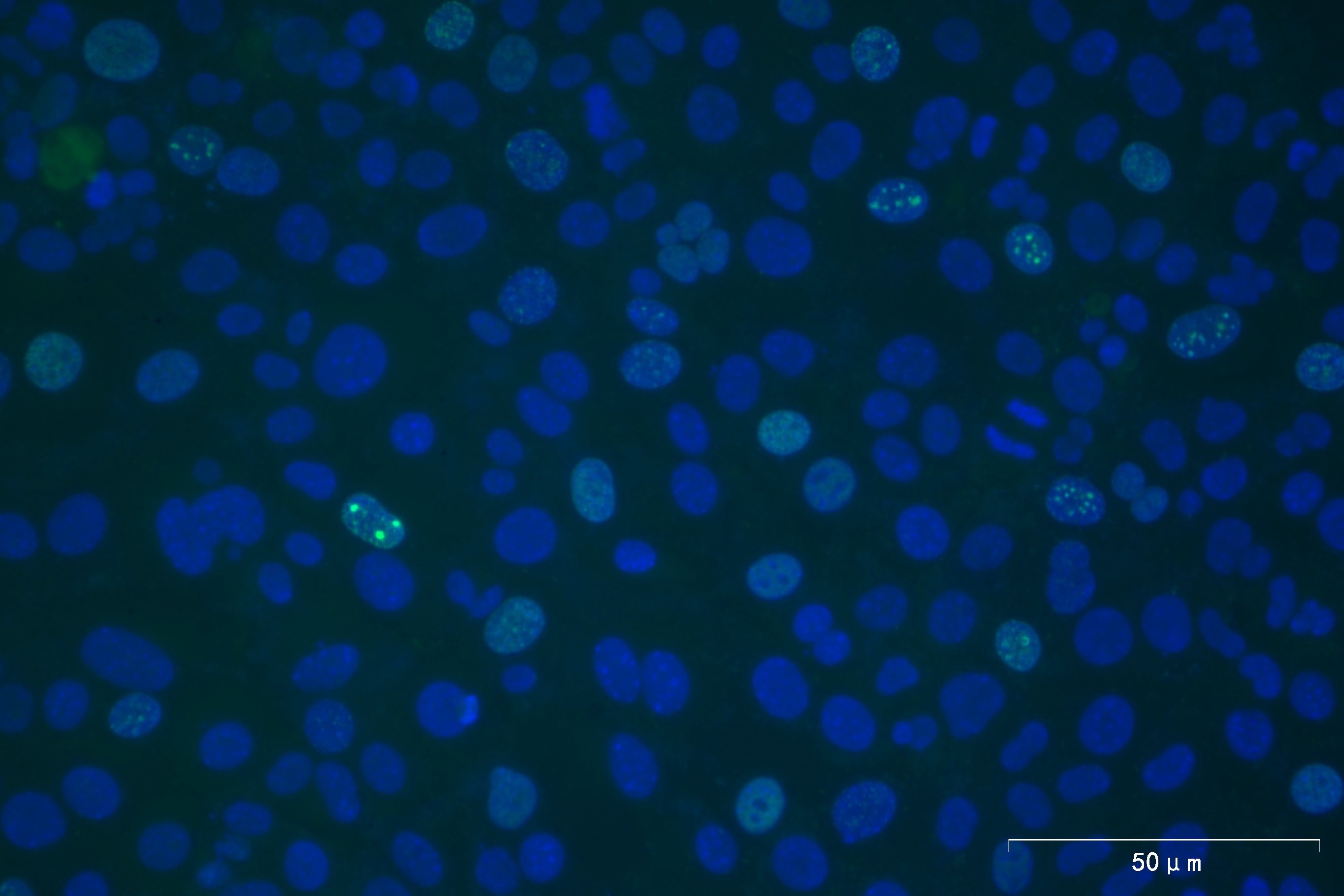

Supplement: Supplementary file 11 — Source data Fig. 9 [file 44318_2025_362_MOESM11_ESM.zip › Figure 9/9I/SLC13A2+Vehicle/Merge.jpg]

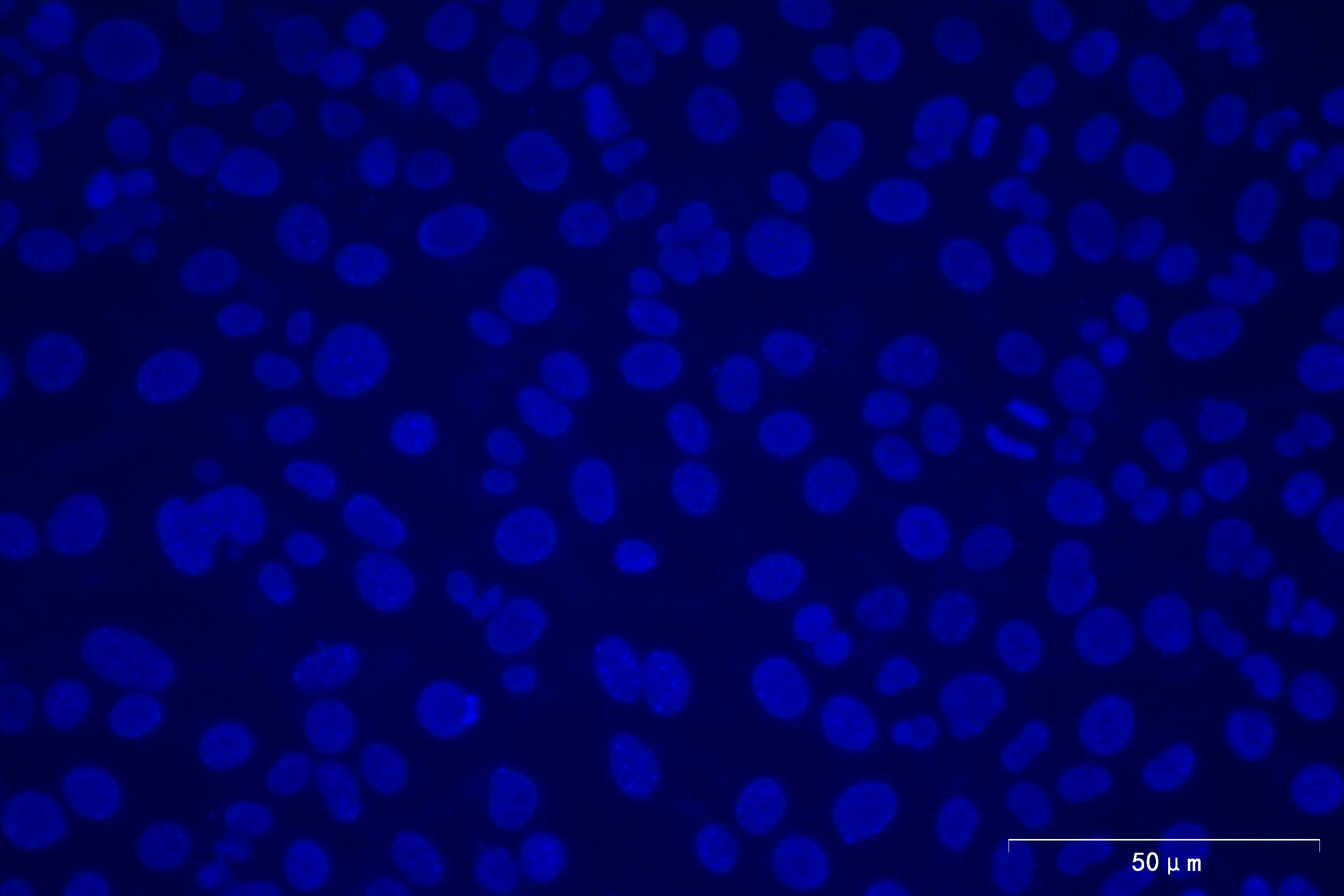

Supplement: Supplementary file 11 — Source data Fig. 9 [file 44318_2025_362_MOESM11_ESM.zip › Figure 9/9I/SLC13A2+Vehicle/DAPI.jpg]

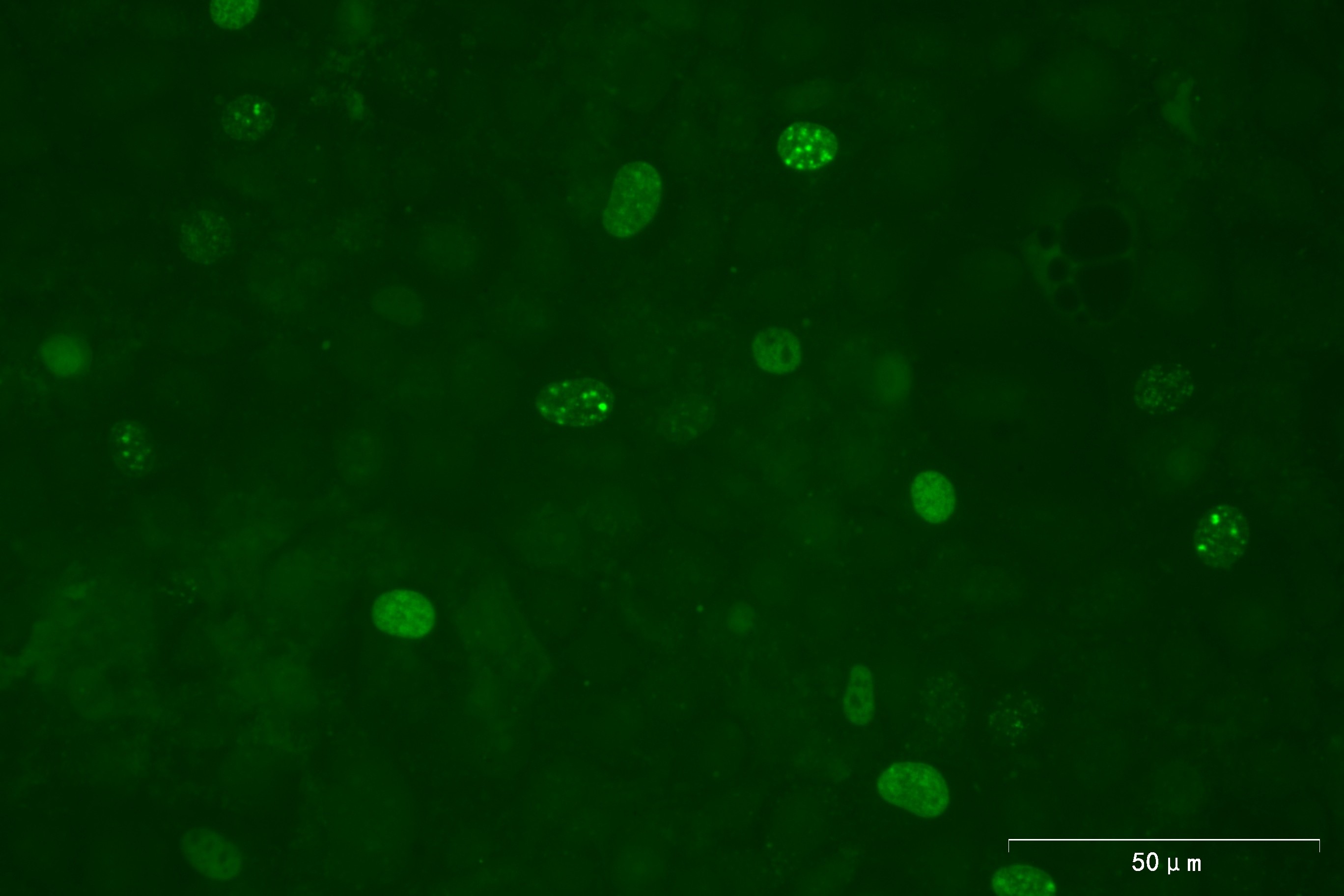

Supplement: Supplementary file 11 — Source data Fig. 9 [file 44318_2025_362_MOESM11_ESM.zip › Figure 9/9I/SLC13A2+ACLYi+Cholesterol/EdU.jpg]

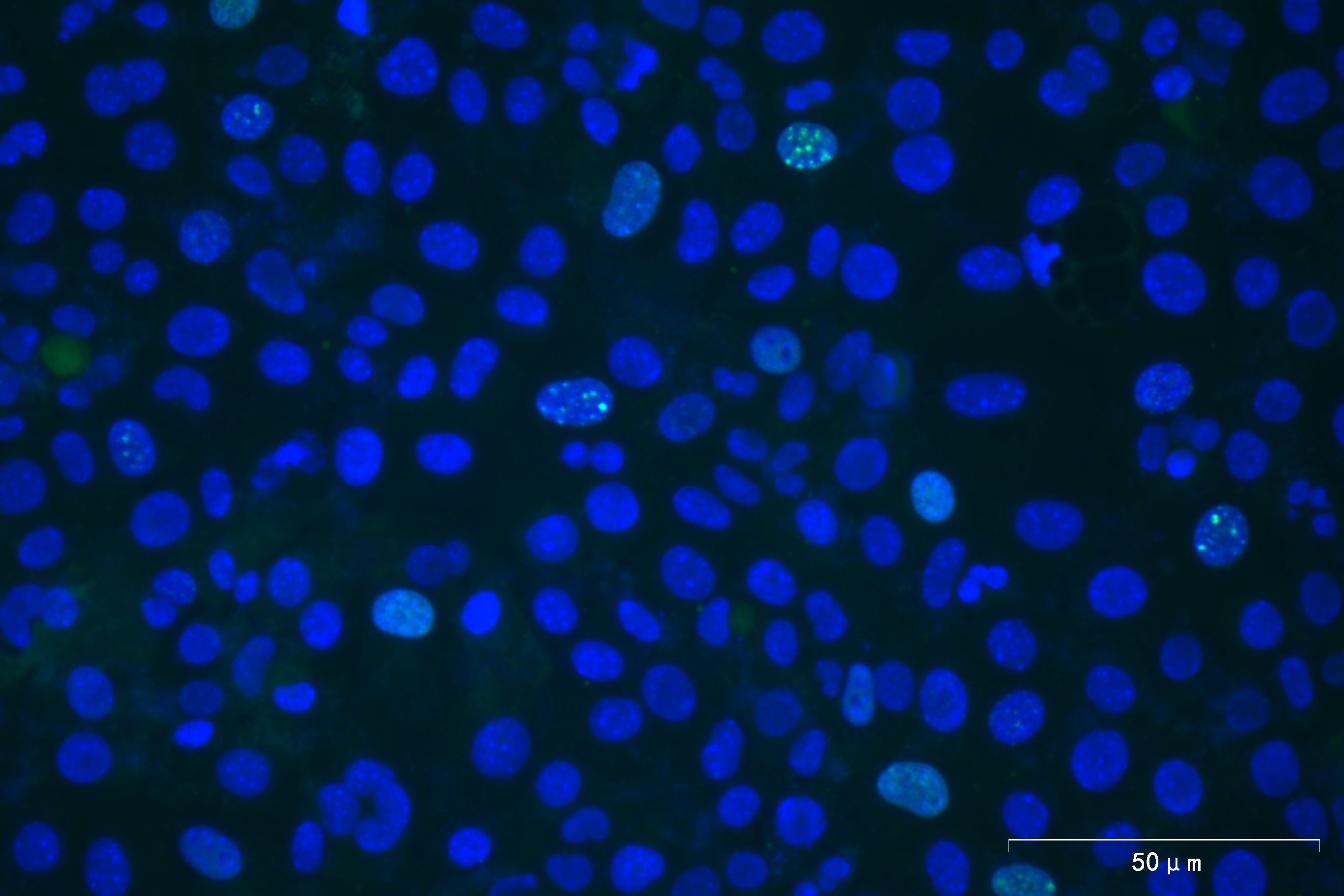

Supplement: Supplementary file 11 — Source data Fig. 9 [file 44318_2025_362_MOESM11_ESM.zip › Figure 9/9I/SLC13A2+ACLYi+Cholesterol/Merge.jpg]

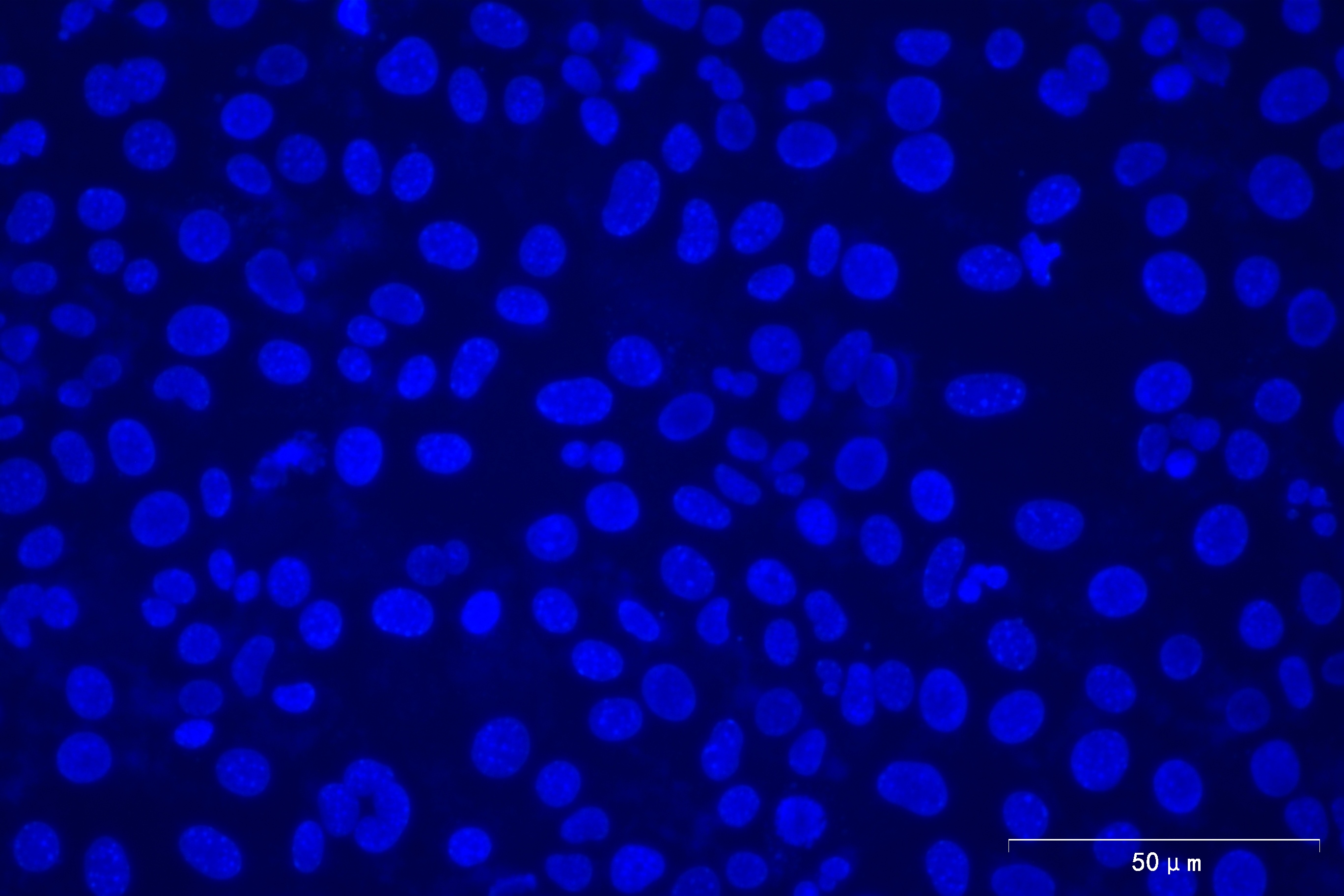

Supplement: Supplementary file 11 — Source data Fig. 9 [file 44318_2025_362_MOESM11_ESM.zip › Figure 9/9I/SLC13A2+ACLYi+Cholesterol/DAPI.jpg]

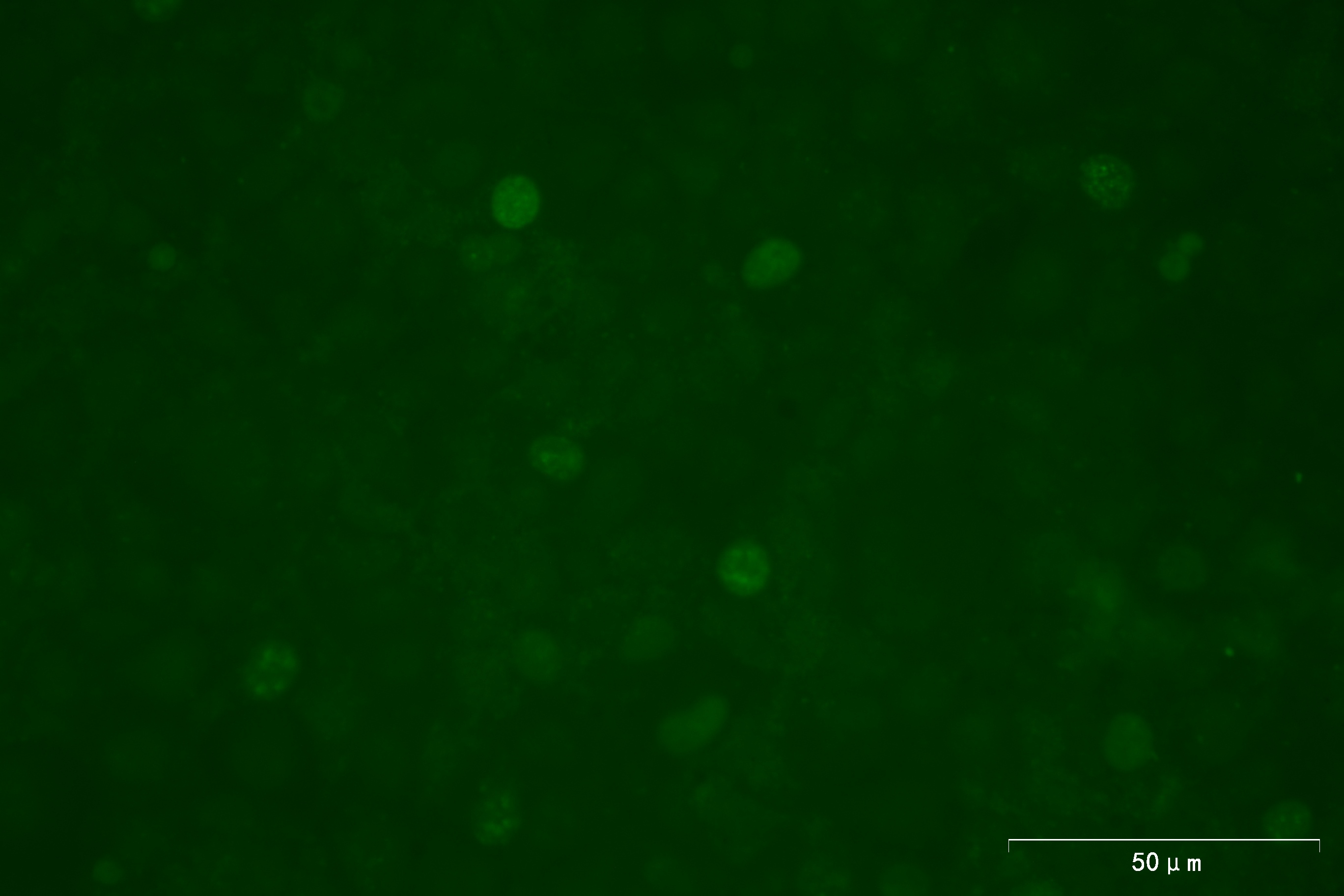

Supplement: Supplementary file 11 — Source data Fig. 9 [file 44318_2025_362_MOESM11_ESM.zip › Figure 9/9I/SLC13A2+ACLYi/EdU.jpg]

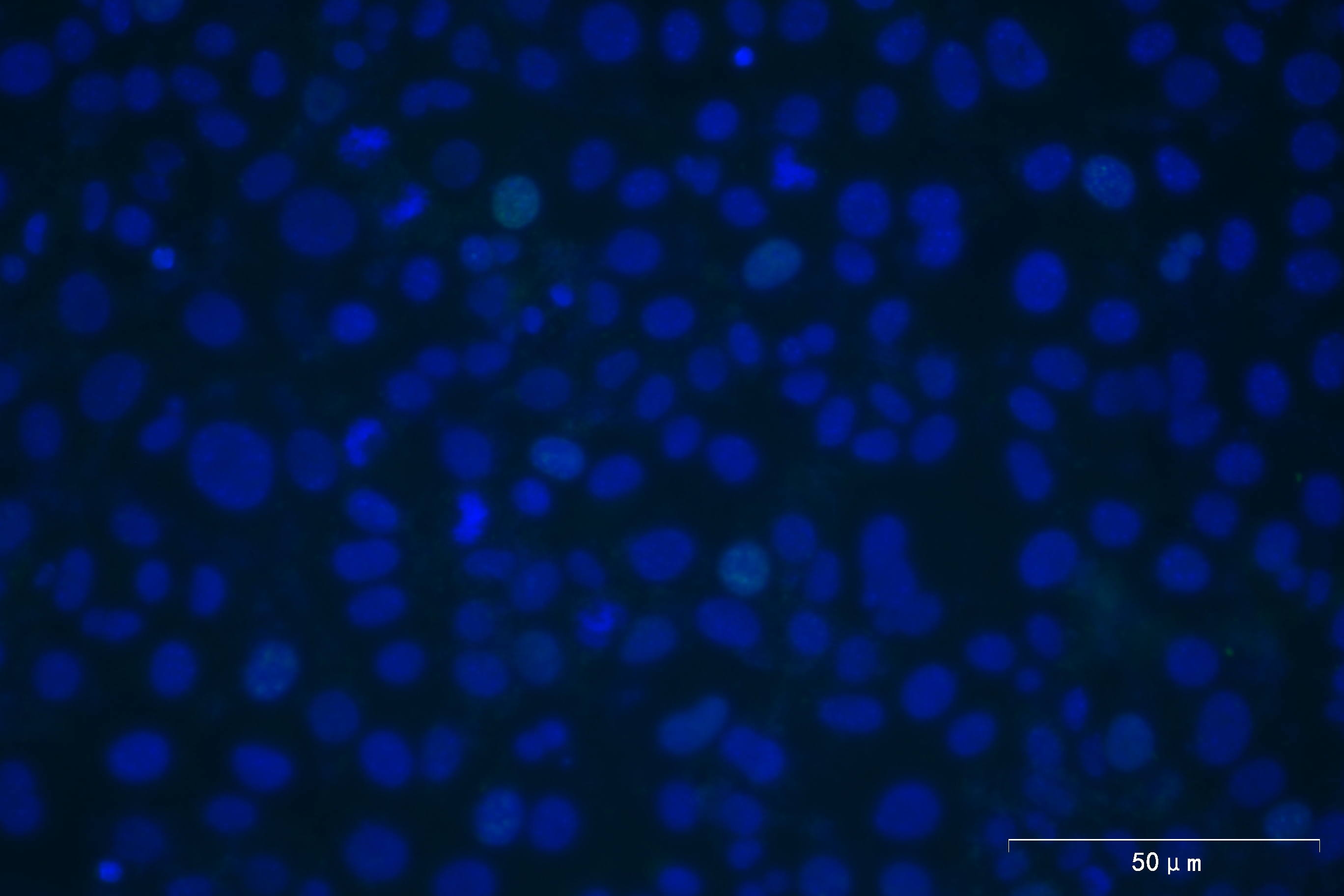

Supplement: Supplementary file 11 — Source data Fig. 9 [file 44318_2025_362_MOESM11_ESM.zip › Figure 9/9I/SLC13A2+ACLYi/Merge.jpg]

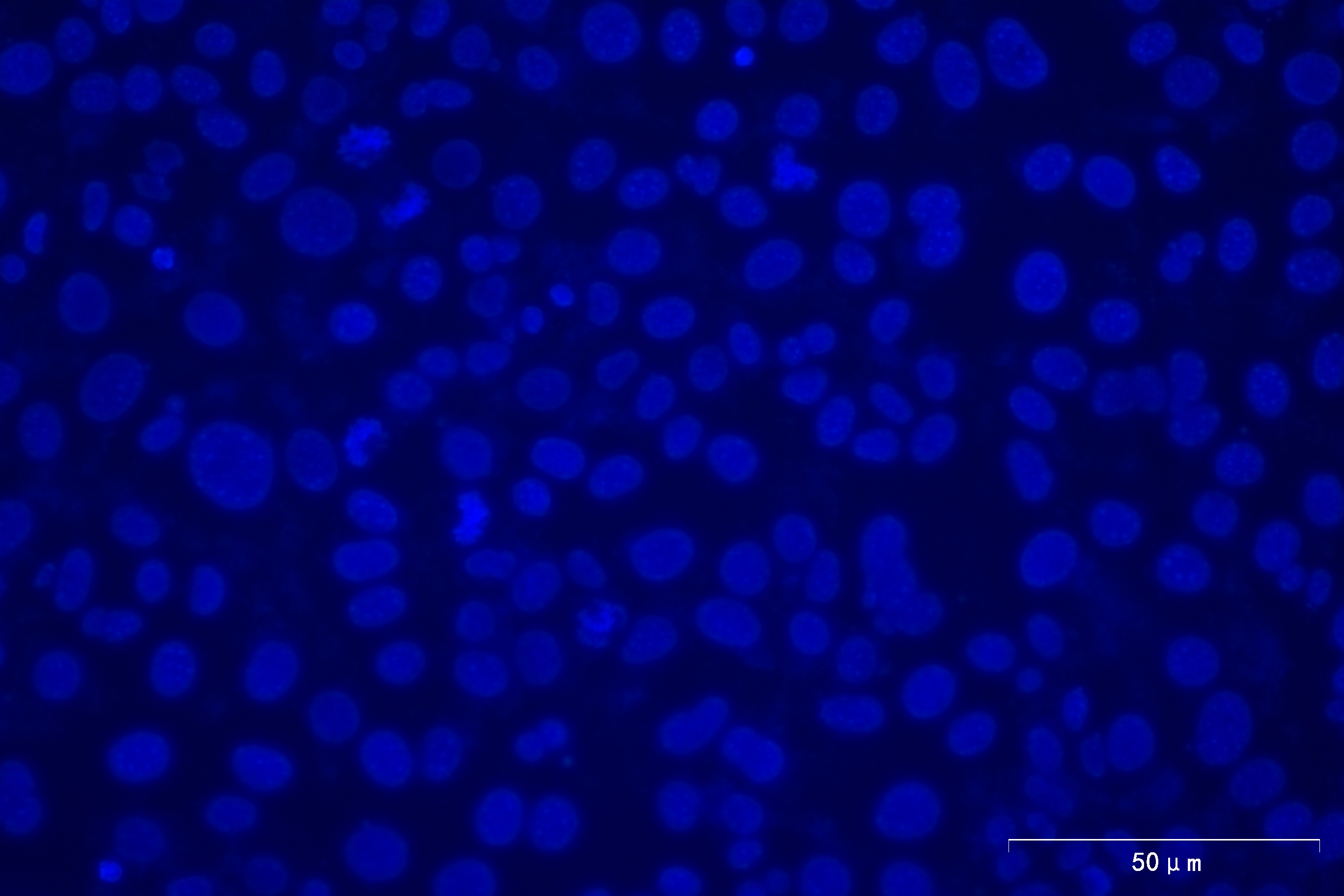

Supplement: Supplementary file 11 — Source data Fig. 9 [file 44318_2025_362_MOESM11_ESM.zip › Figure 9/9I/SLC13A2+ACLYi/DAPI.jpg]

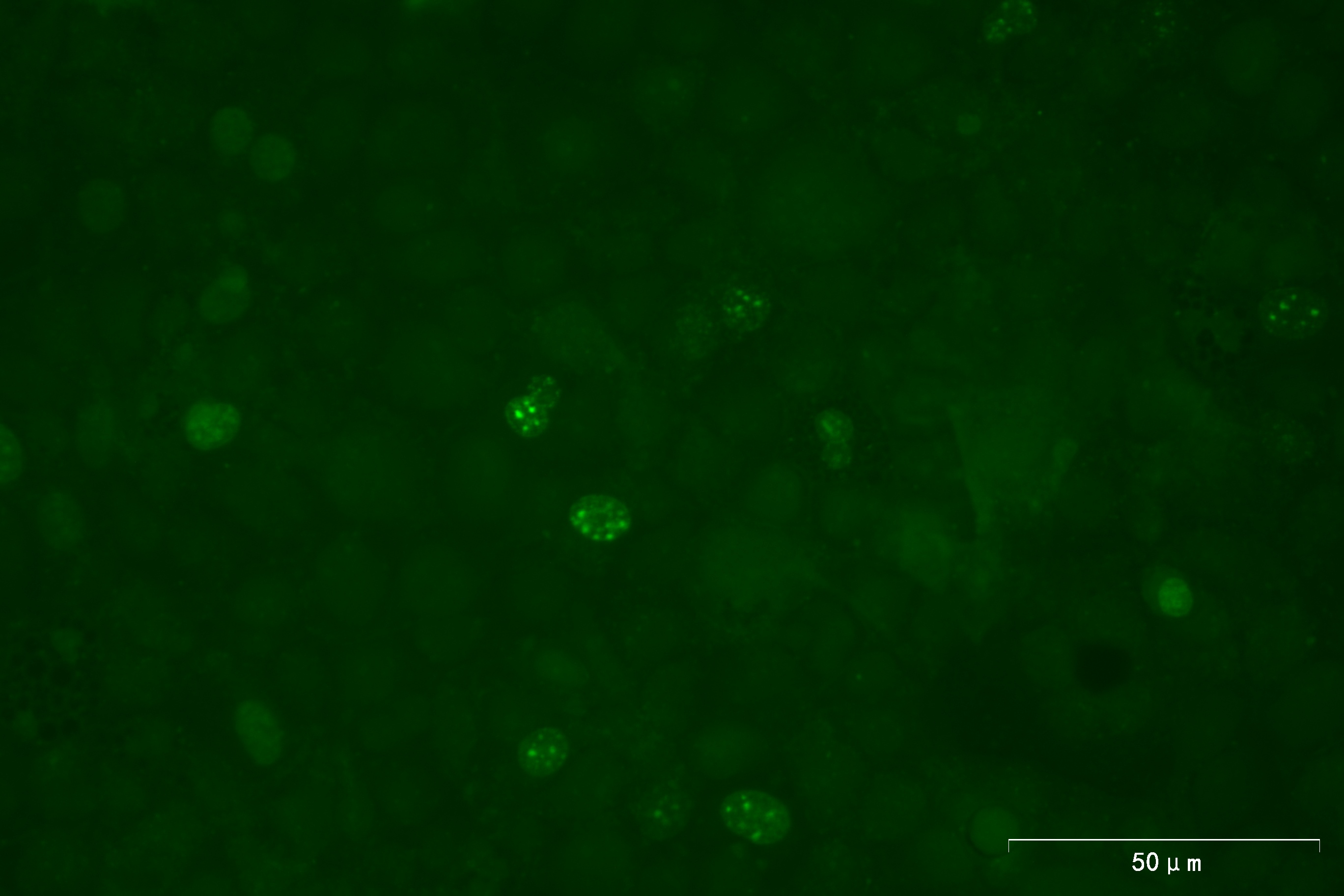

Supplement: Supplementary file 11 — Source data Fig. 9 [file 44318_2025_362_MOESM11_ESM.zip › Figure 9/9I/Vector+Vehicle/EdU.jpg]

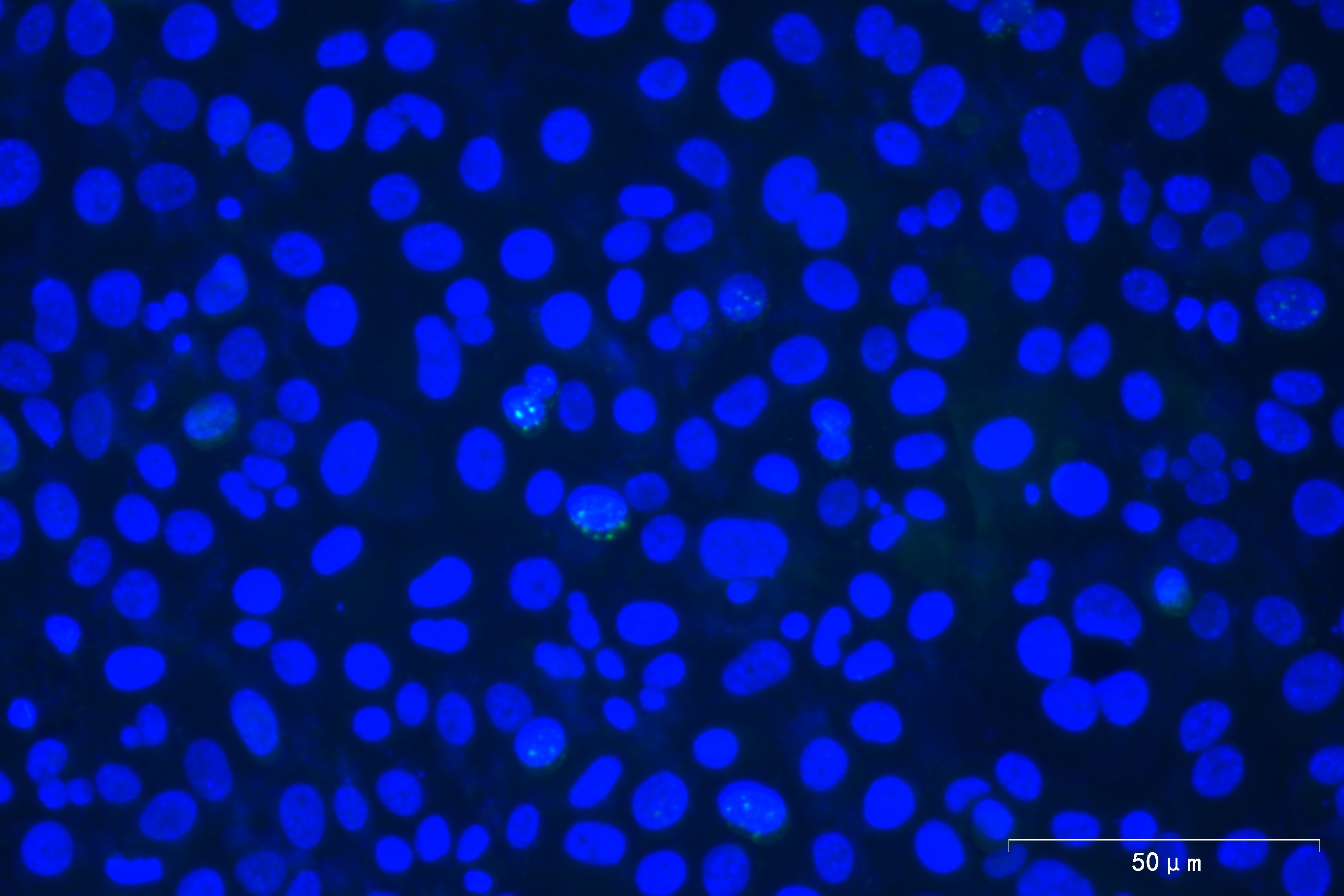

Supplement: Supplementary file 11 — Source data Fig. 9 [file 44318_2025_362_MOESM11_ESM.zip › Figure 9/9I/Vector+Vehicle/Merge.jpg]

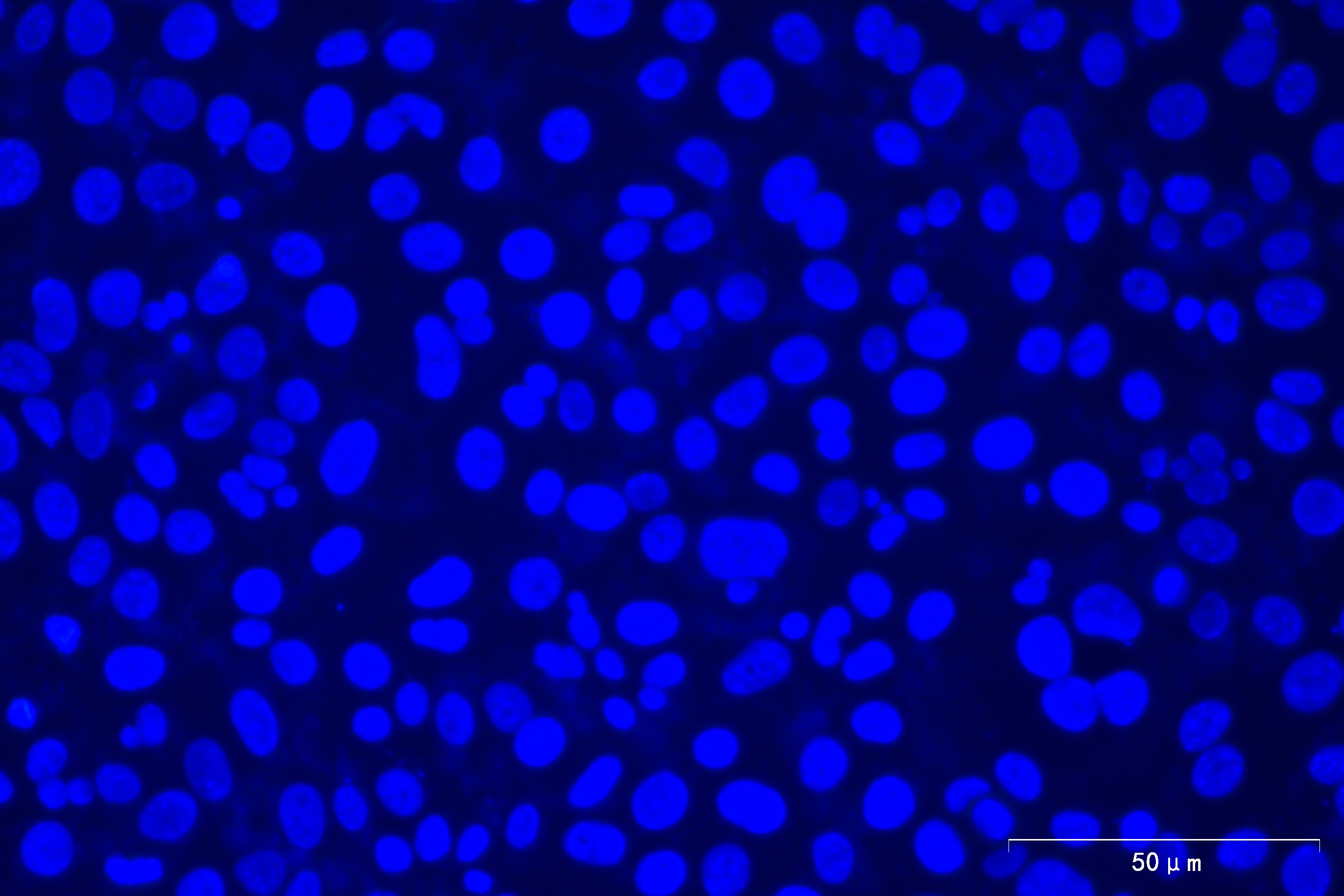

Supplement: Supplementary file 11 — Source data Fig. 9 [file 44318_2025_362_MOESM11_ESM.zip › Figure 9/9I/Vector+Vehicle/DAPI.jpg]

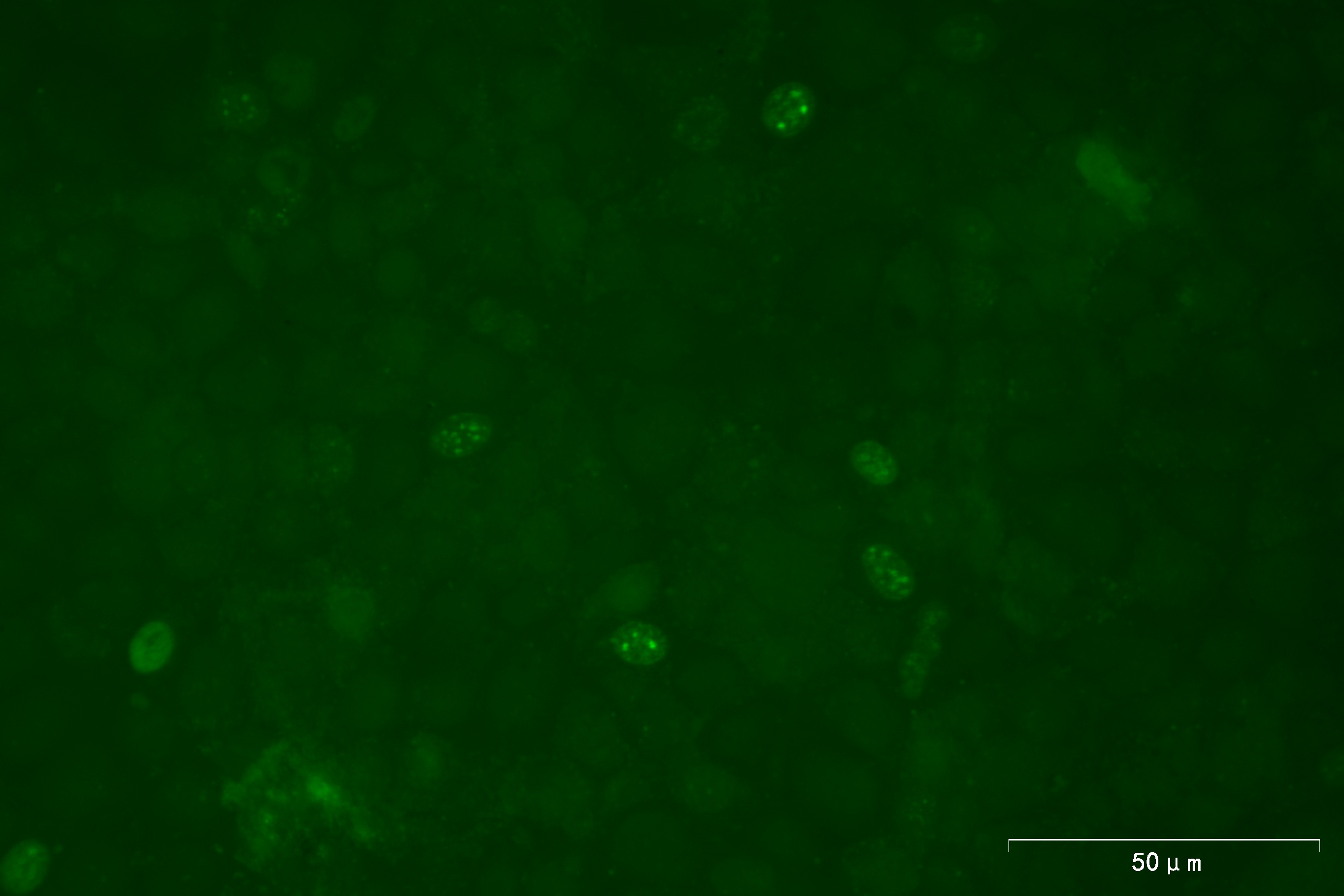

Supplement: Supplementary file 11 — Source data Fig. 9 [file 44318_2025_362_MOESM11_ESM.zip › Figure 9/9I/Vector+ACLYi+Cholesterol/EdU.jpg]

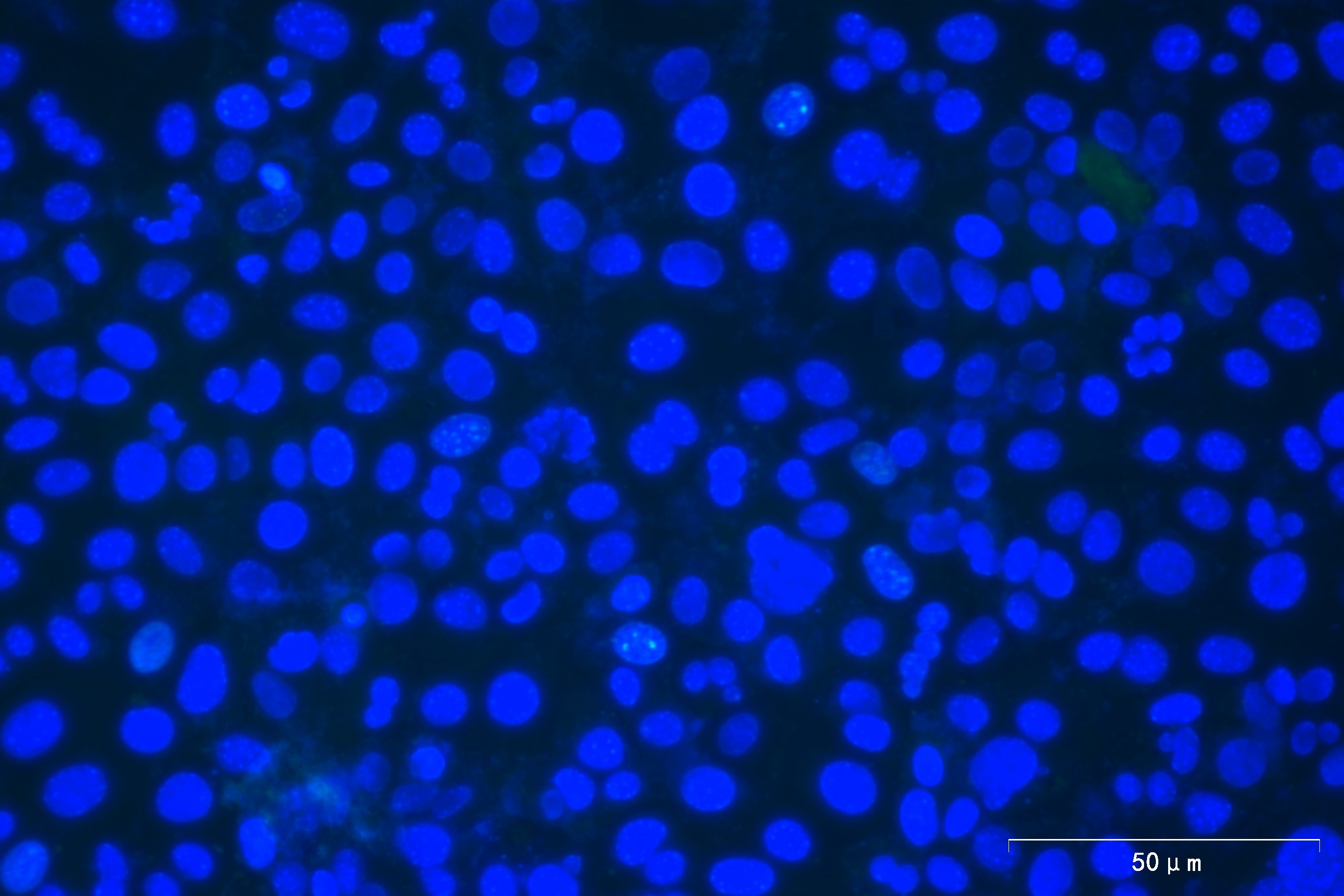

Supplement: Supplementary file 11 — Source data Fig. 9 [file 44318_2025_362_MOESM11_ESM.zip › Figure 9/9I/Vector+ACLYi+Cholesterol/Merge.jpg]

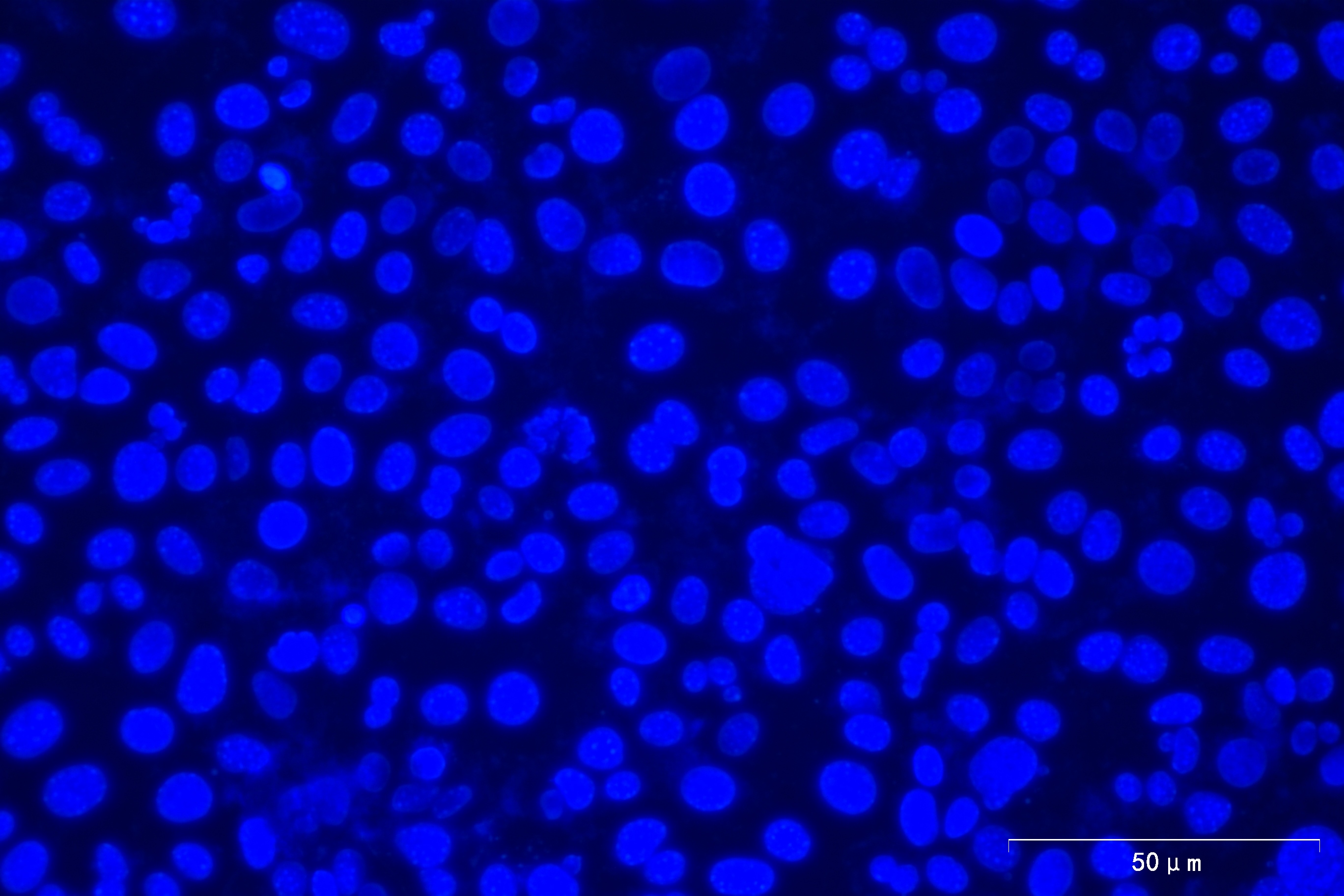

Supplement: Supplementary file 11 — Source data Fig. 9 [file 44318_2025_362_MOESM11_ESM.zip › Figure 9/9I/Vector+ACLYi+Cholesterol/DAPI.jpg]

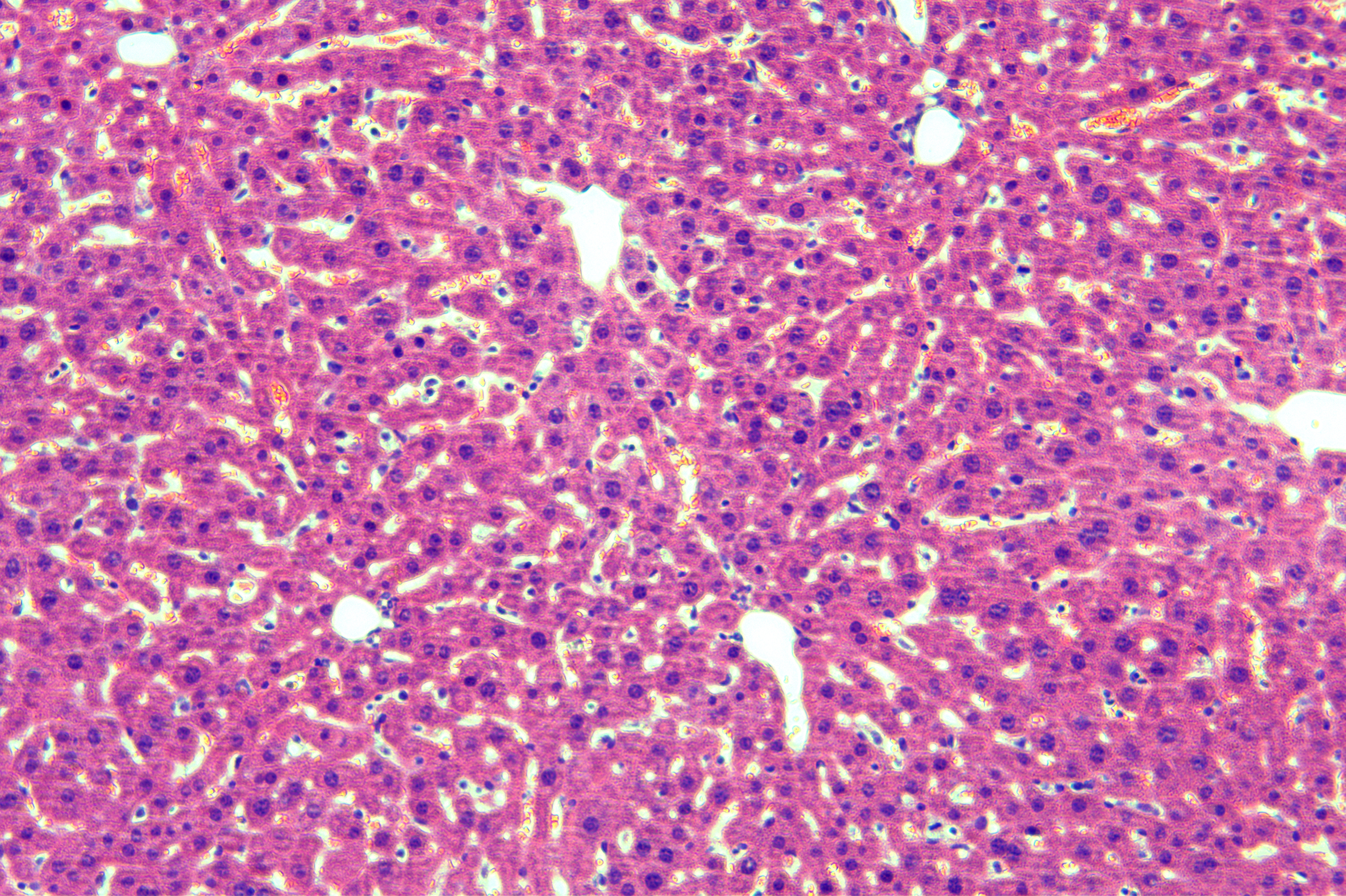

Supplement: Supplementary file 11 — Source data Fig. 9 [file 44318_2025_362_MOESM11_ESM.zip › Figure 9/9G/HE/SLC-ACLYi-1.jpg]

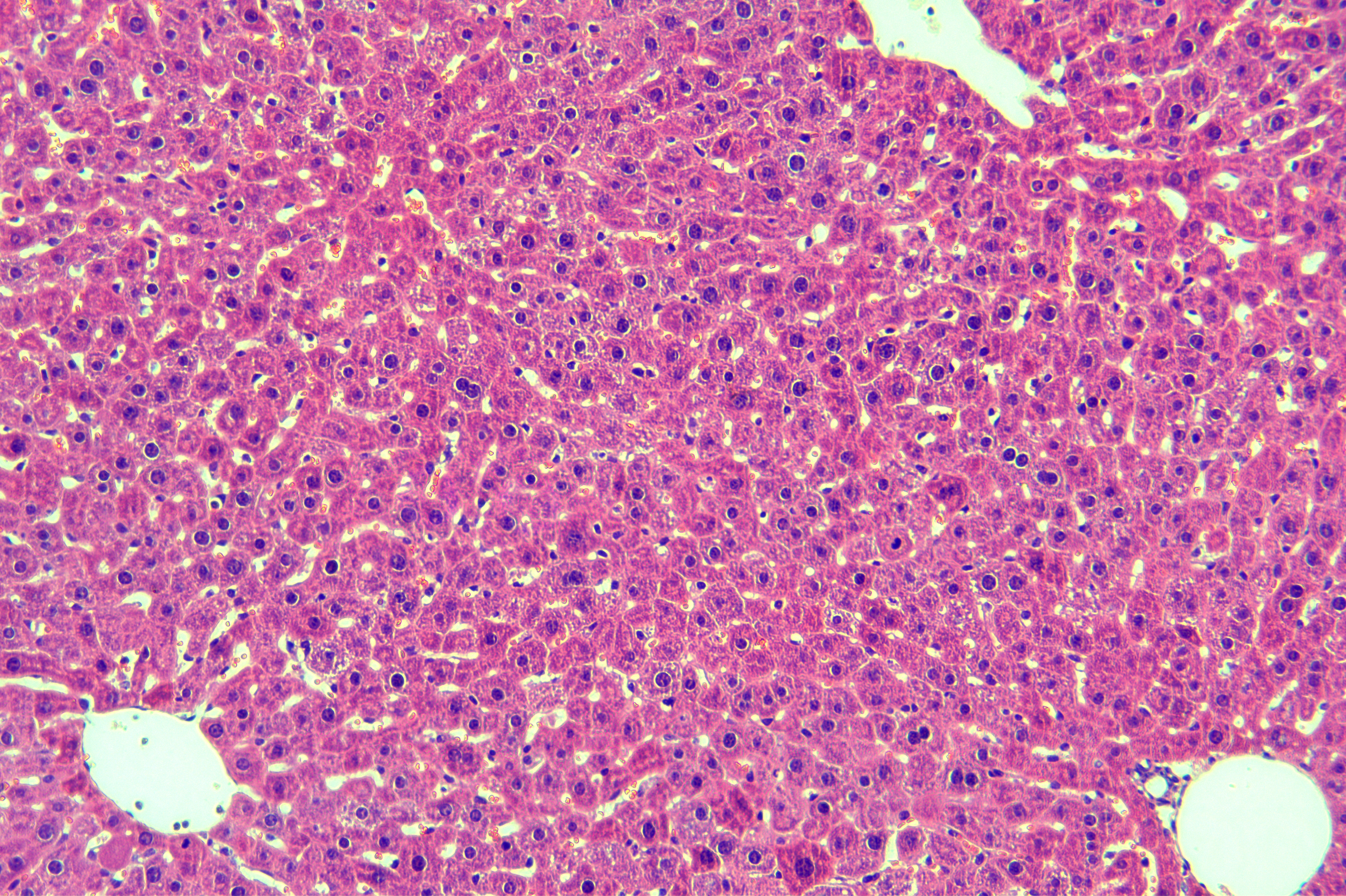

Supplement: Supplementary file 11 — Source data Fig. 9 [file 44318_2025_362_MOESM11_ESM.zip › Figure 9/9G/HE/SLC-1.jpg]

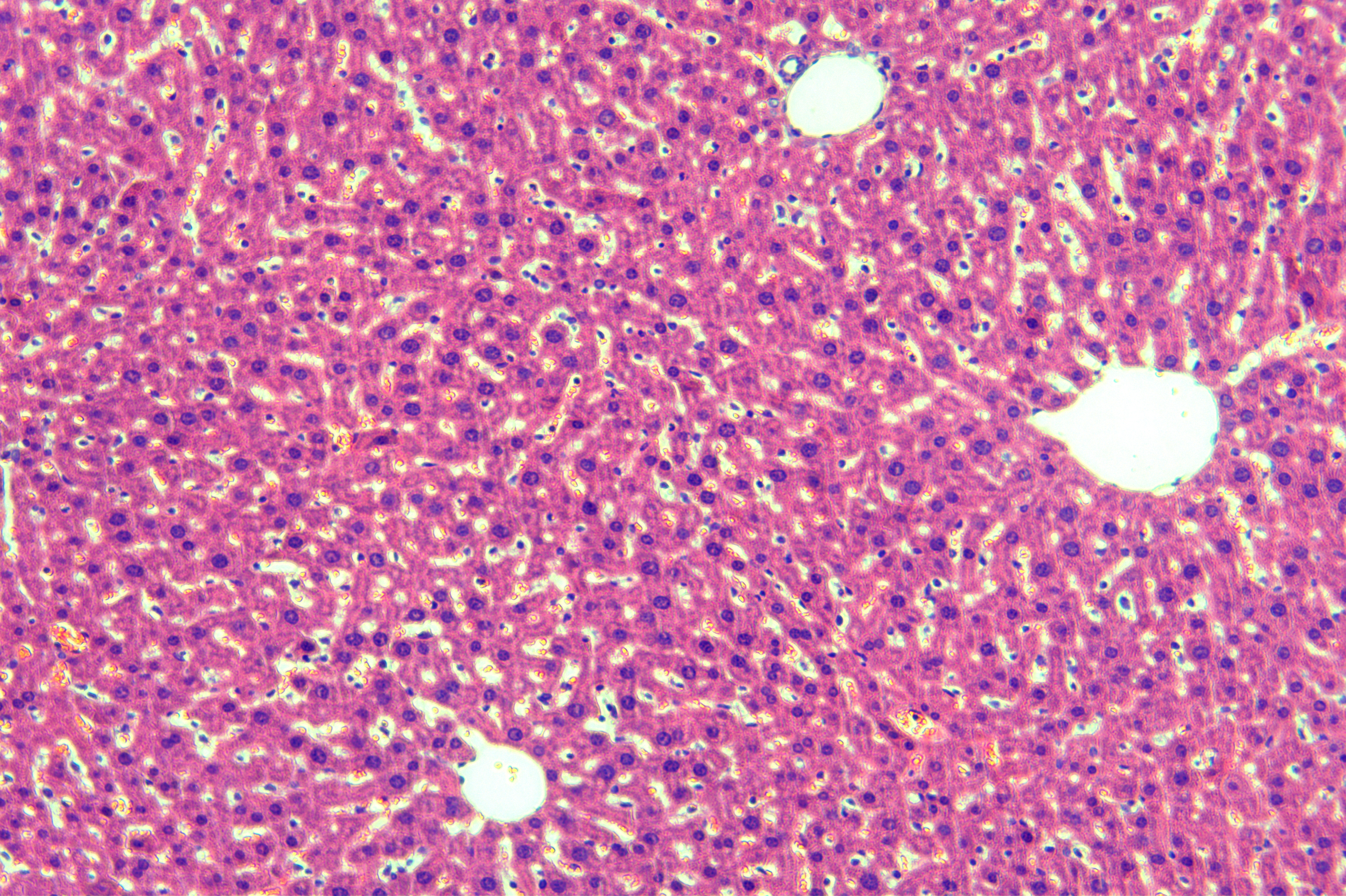

Supplement: Supplementary file 11 — Source data Fig. 9 [file 44318_2025_362_MOESM11_ESM.zip › Figure 9/9G/HE/GFP-ACLYi-1.jpg]

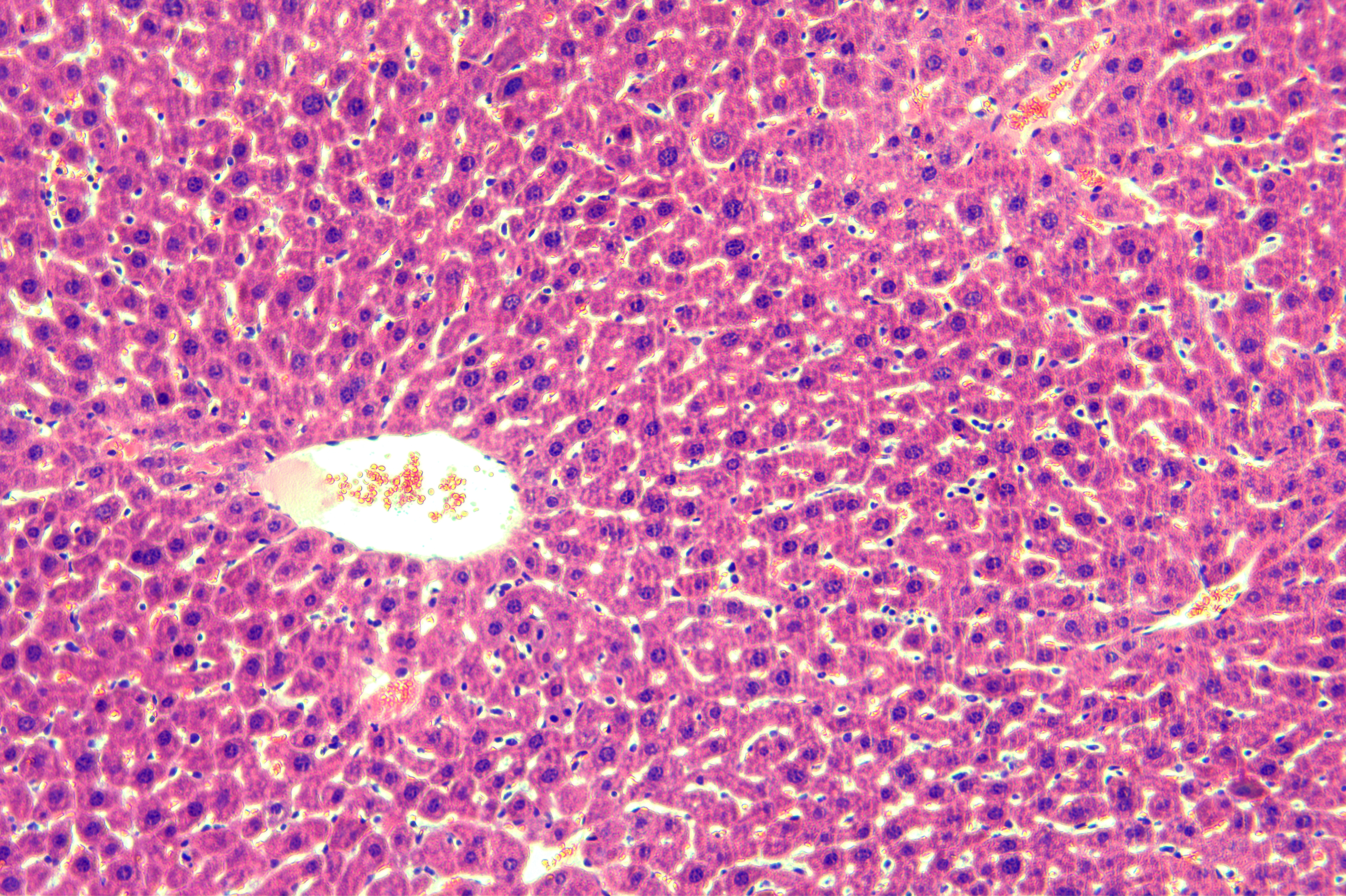

Supplement: Supplementary file 11 — Source data Fig. 9 [file 44318_2025_362_MOESM11_ESM.zip › Figure 9/9G/HE/GFP-1.jpg]

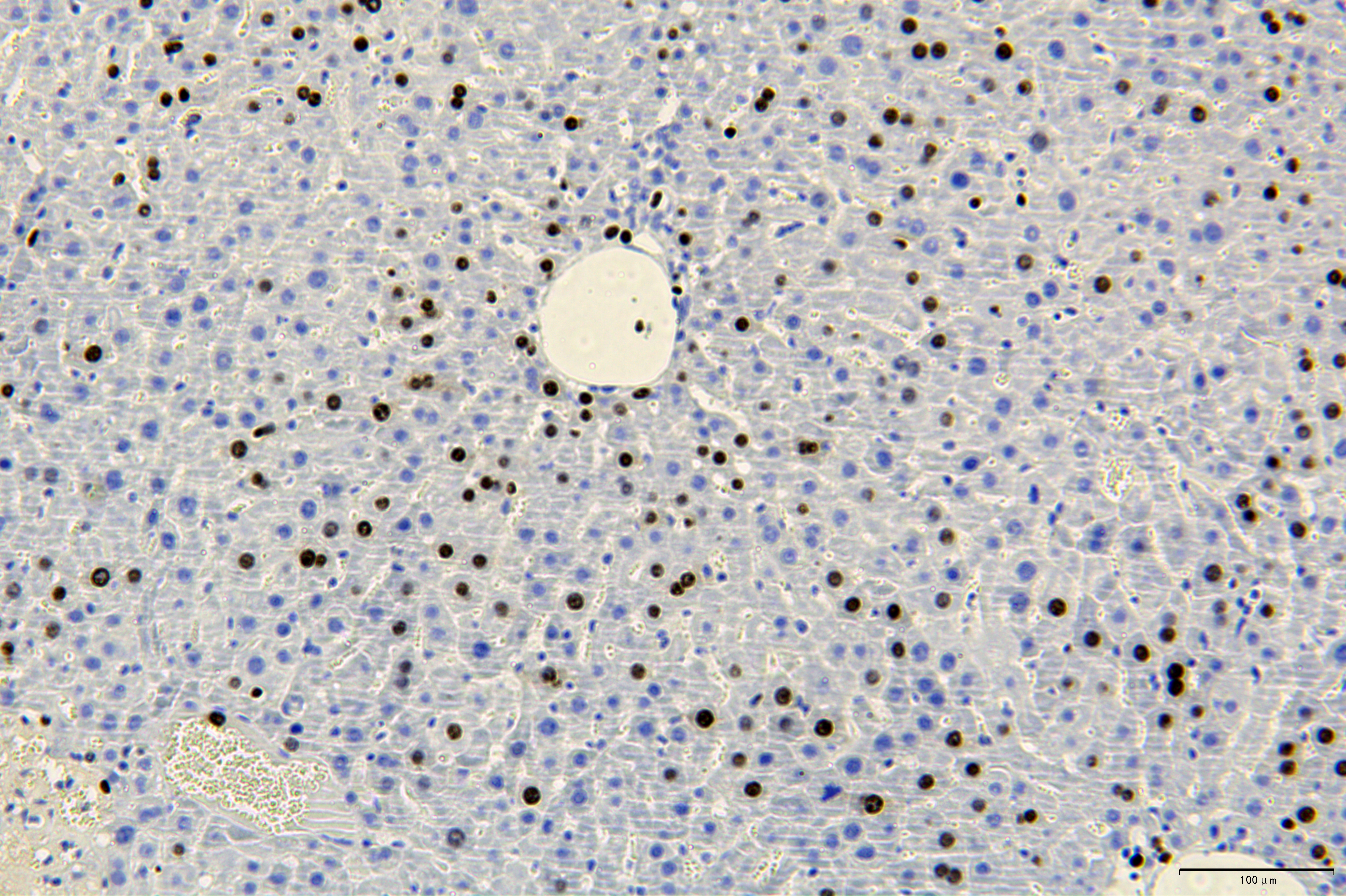

Supplement: Supplementary file 11 — Source data Fig. 9 [file 44318_2025_362_MOESM11_ESM.zip › Figure 9/9G/BrdU/SLC-1.jpg]

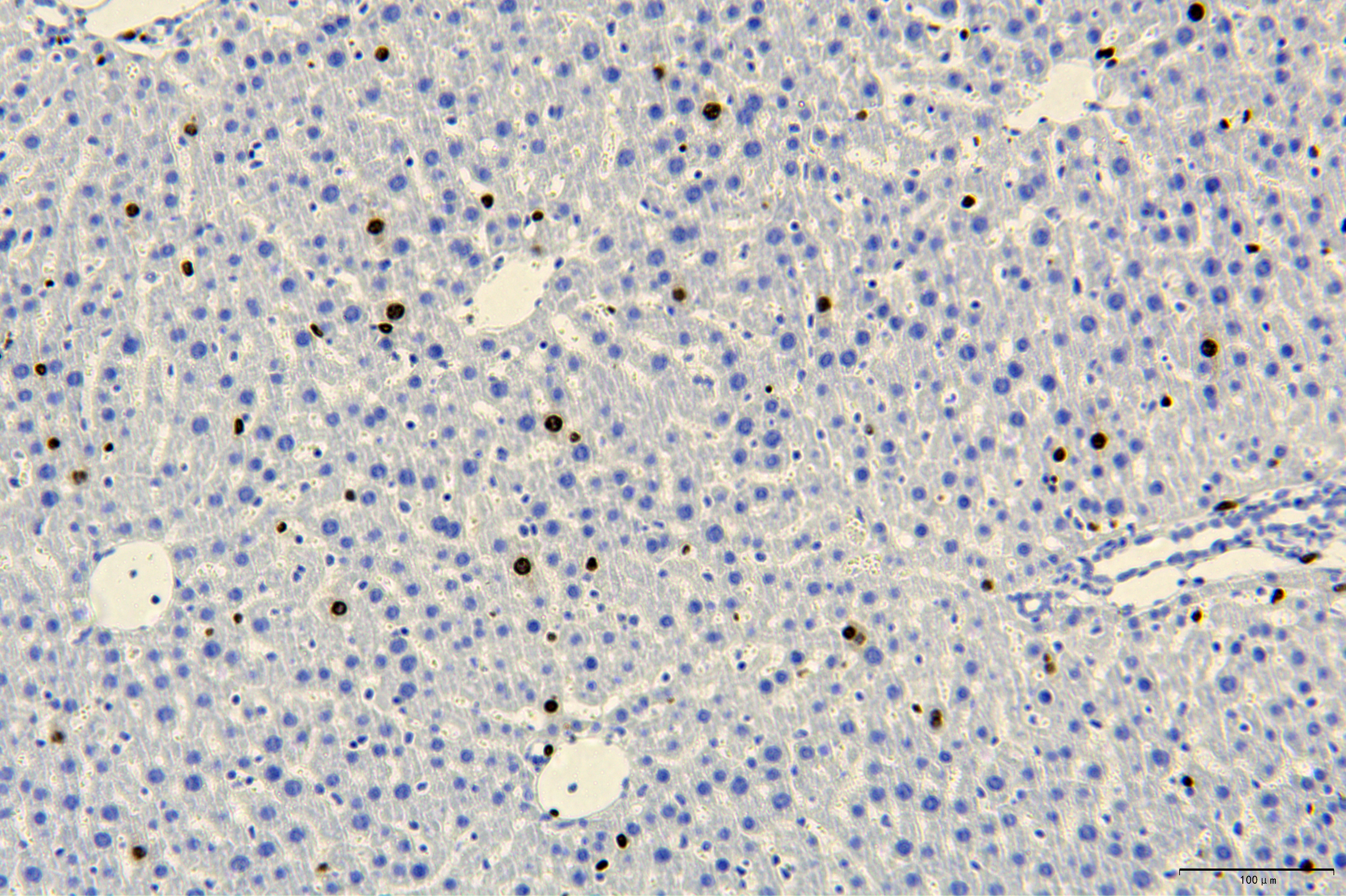

Supplement: Supplementary file 11 — Source data Fig. 9 [file 44318_2025_362_MOESM11_ESM.zip › Figure 9/9G/BrdU/GFP+ACLYi-1.jpg]

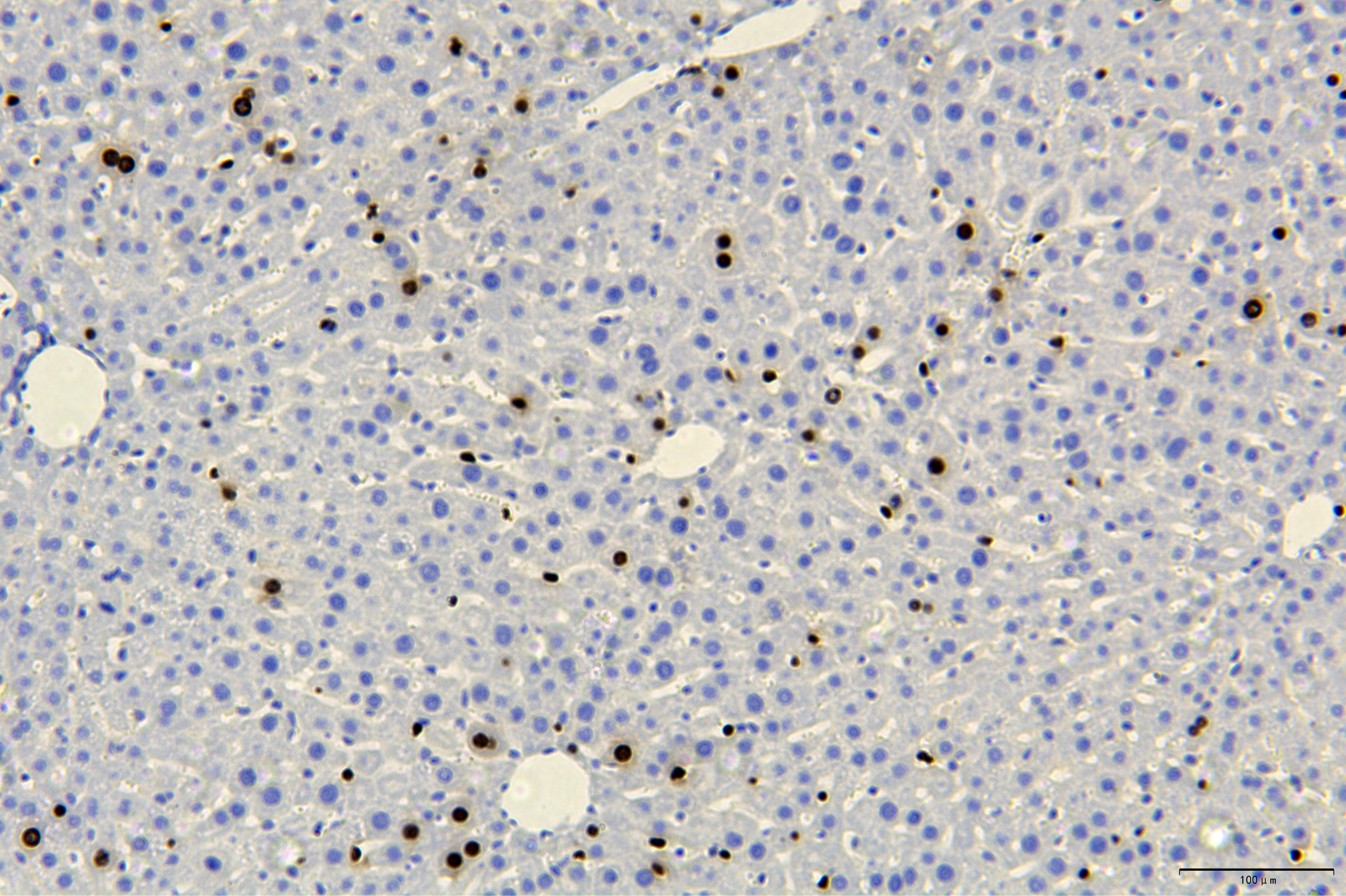

Supplement: Supplementary file 11 — Source data Fig. 9 [file 44318_2025_362_MOESM11_ESM.zip › Figure 9/9G/BrdU/SLC+ACLYi-1.jpg]

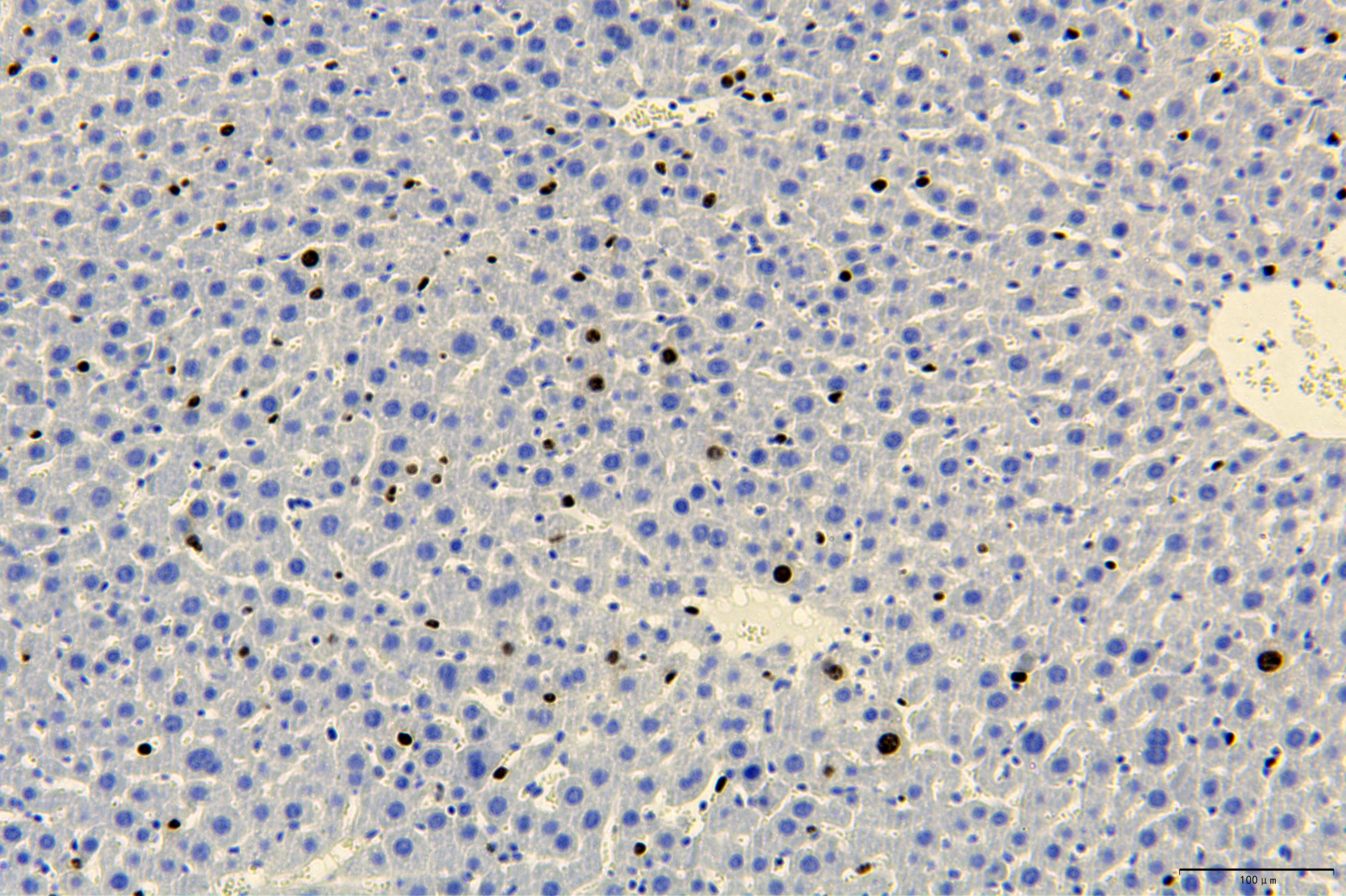

Supplement: Supplementary file 11 — Source data Fig. 9 [file 44318_2025_362_MOESM11_ESM.zip › Figure 9/9G/BrdU/GFP-1.jpg]
