# Supplementary material for: SLC13A2 promotes hepatocyte metabolic remodeling and liver regeneration by enhancing de novo cholesterol biosynthesis
Source: EMBO J. 2025 Jan 17;44(5):1442–63. doi: 10.1038/s44318-025-00362-y (PMC11876347; doi:10.1038/s44318-025-00362-y)
Supplement: Supplementary file 6 — Source data Fig. 4 [file 44318_2025_362_MOESM6_ESM.zip › Figure 4/4B.pptx]

## Slide 1
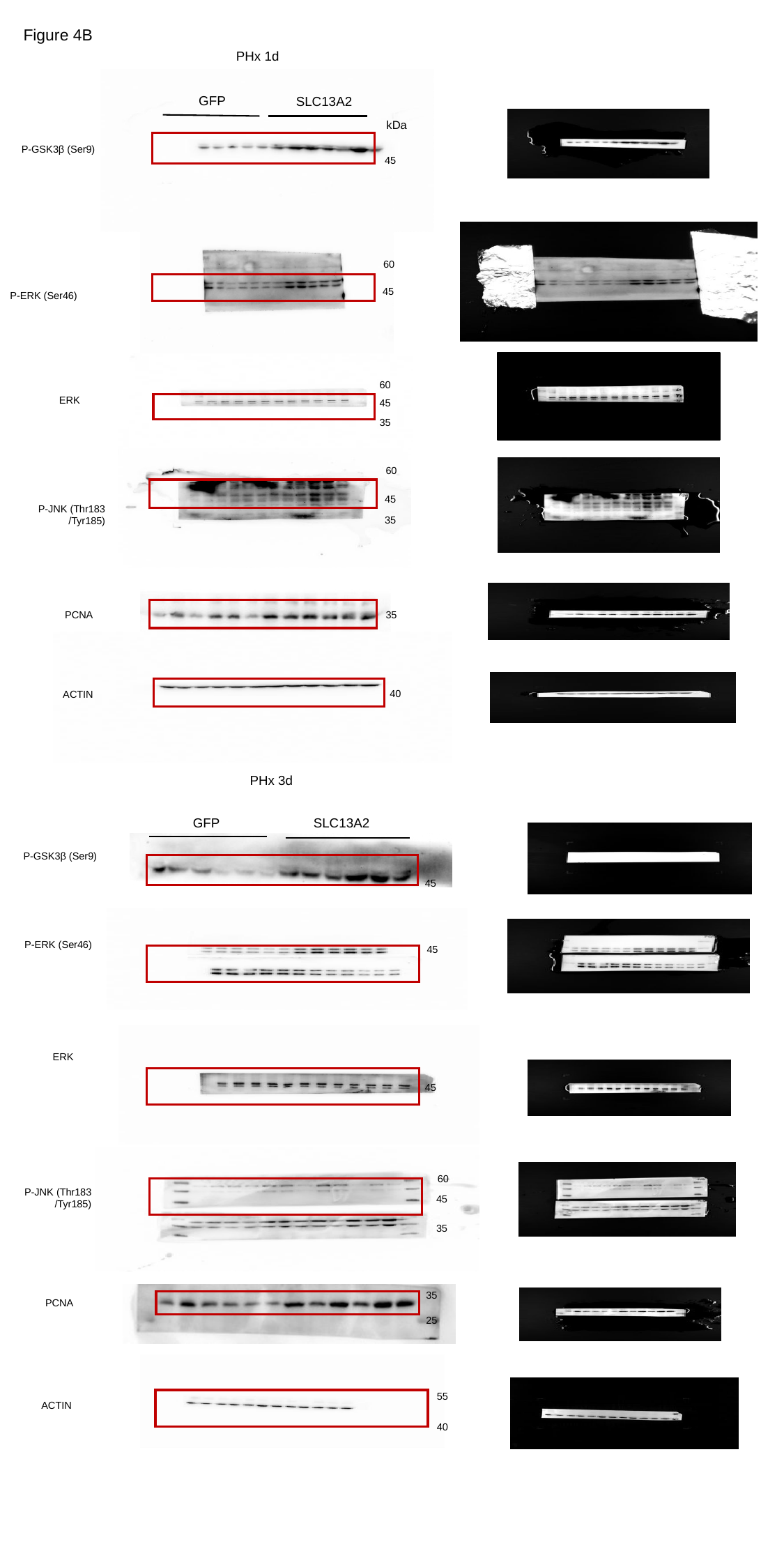

Figure 4B
PHx 1d
GFP
SLC13A2
kDa
P-GSK3β (Ser9)
45
60
45
P-ERK (Ser46)
60
ERK
45
35
60
45
 P-JNK (Thr183
 /Tyr185)
35
35
PCNA
40
ACTIN
PHx 3d
SLC13A2
GFP
P-GSK3β (Ser9)
45
P-ERK (Ser46)
45
ERK
45
60
 P-JNK (Thr183
 /Tyr185)
45
35
35
PCNA
25
55
ACTIN
40

## Slide 2
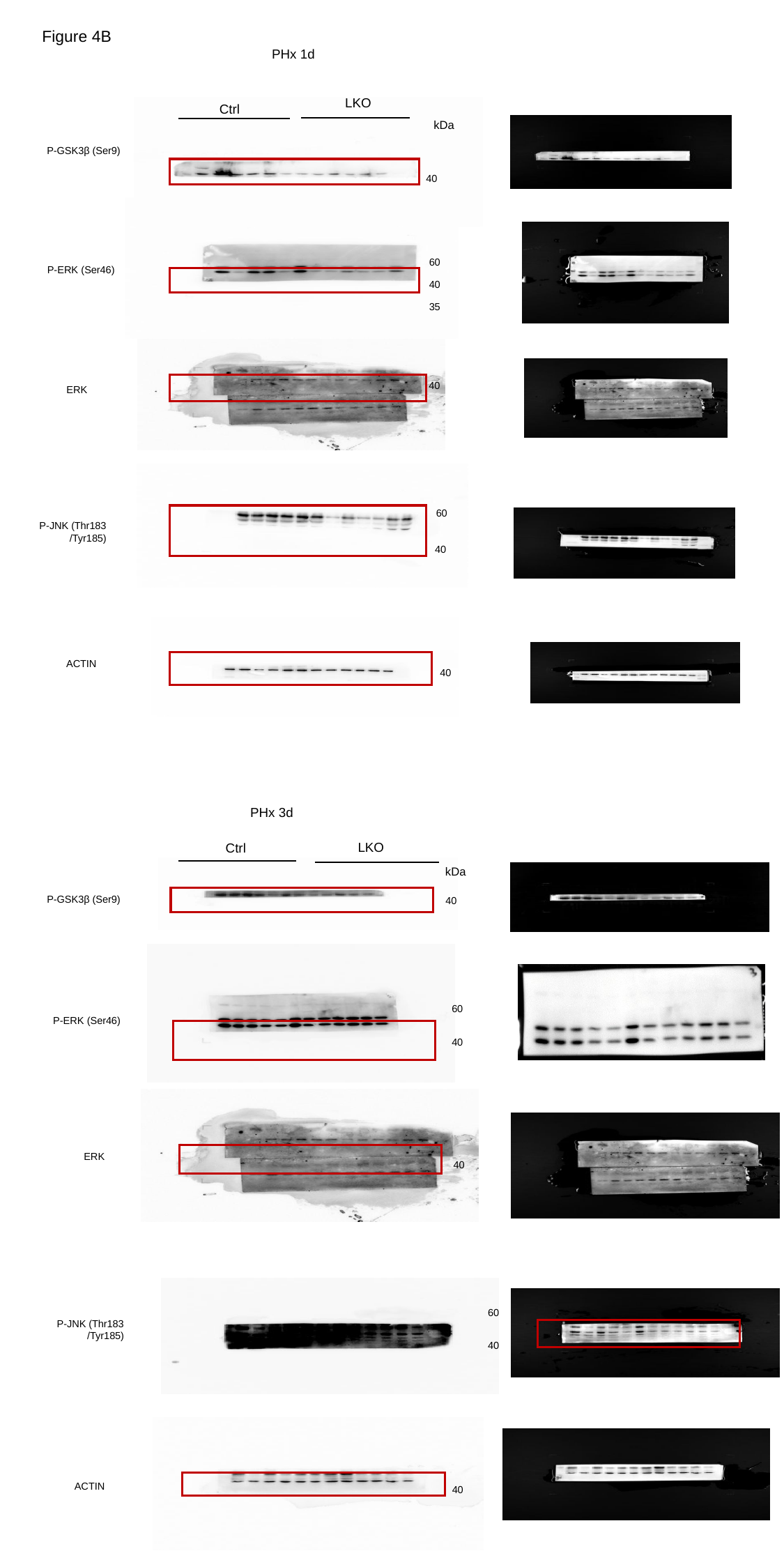

Figure 4B
PHx 1d
LKO
Ctrl
kDa
P-GSK3β (Ser9)
40
60
P-ERK (Ser46)
40
35
40
ERK
60
 P-JNK (Thr183
 /Tyr185)
40
ACTIN
40
PHx 3d
LKO
Ctrl
kDa
P-GSK3β (Ser9)
40
60
P-ERK (Ser46)
40
ERK
40
60
 P-JNK (Thr183
 /Tyr185)
40
ACTIN
40
