# Supplementary material for: SLC13A2 promotes hepatocyte metabolic remodeling and liver regeneration by enhancing de novo cholesterol biosynthesis
Source: EMBO J. 2025 Jan 17;44(5):1442–63. doi: 10.1038/s44318-025-00362-y (PMC11876347; doi:10.1038/s44318-025-00362-y)
Supplement: Supplementary file 7 — Source data Fig. 5 [file 44318_2025_362_MOESM7_ESM.zip › Figure 5/5D.pptx]

## Slide 1
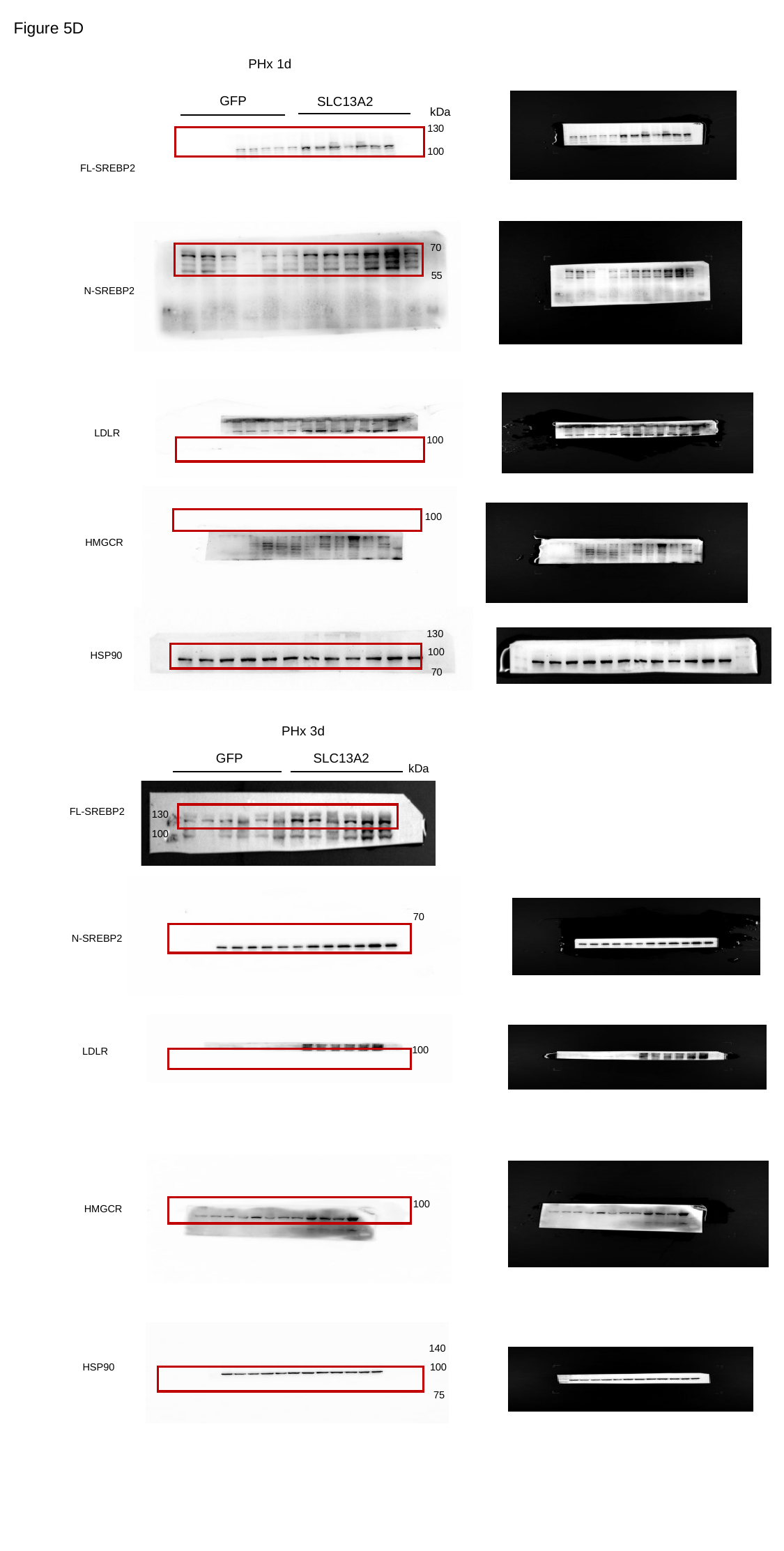

Figure 5D
PHx 1d
GFP
SLC13A2
kDa
130
100
FL-SREBP2
70
55
N-SREBP2
LDLR
100
100
HMGCR
130
100
HSP90
70
PHx 3d
GFP
SLC13A2
kDa
FL-SREBP2
130
100
70
N-SREBP2
100
LDLR
100
HMGCR
140
HSP90
100
75

## Slide 2
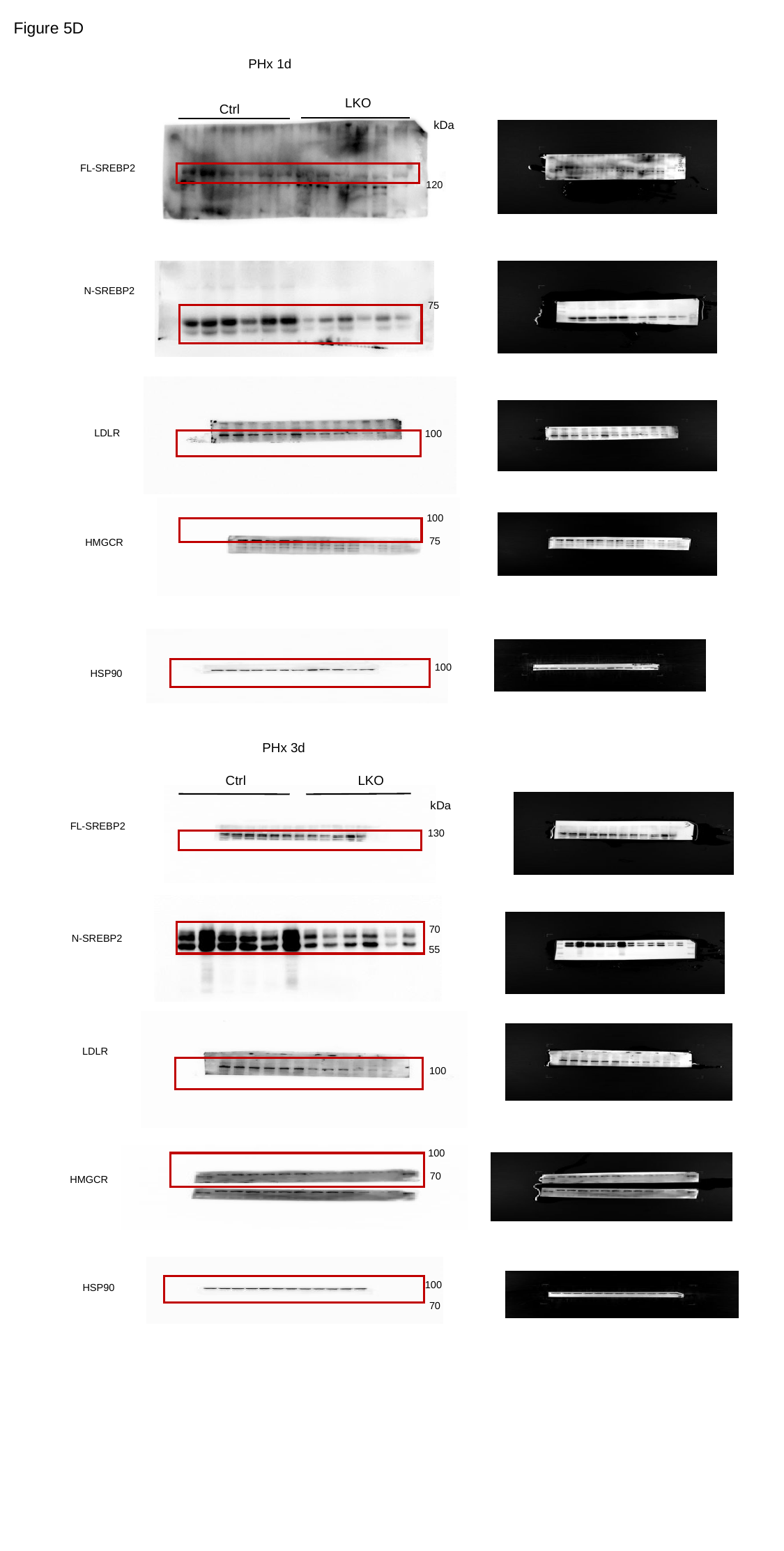

Figure 5D
PHx 1d
LKO
Ctrl
kDa
FL-SREBP2
120
N-SREBP2
75
LDLR
100
100
75
HMGCR
100
HSP90
PHx 3d
LKO
Ctrl
kDa
FL-SREBP2
130
70
N-SREBP2
55
LDLR
100
100
70
HMGCR
100
HSP90
70
