# Supplementary material for: SLC13A2 promotes hepatocyte metabolic remodeling and liver regeneration by enhancing de novo cholesterol biosynthesis
Source: EMBO J. 2025 Jan 17;44(5):1442–63. doi: 10.1038/s44318-025-00362-y (PMC11876347; doi:10.1038/s44318-025-00362-y)
Supplement: Supplementary file 9 — Source data Fig. 7 [file 44318_2025_362_MOESM9_ESM.zip › Figure 7/7H.pptx]

## Slide 1
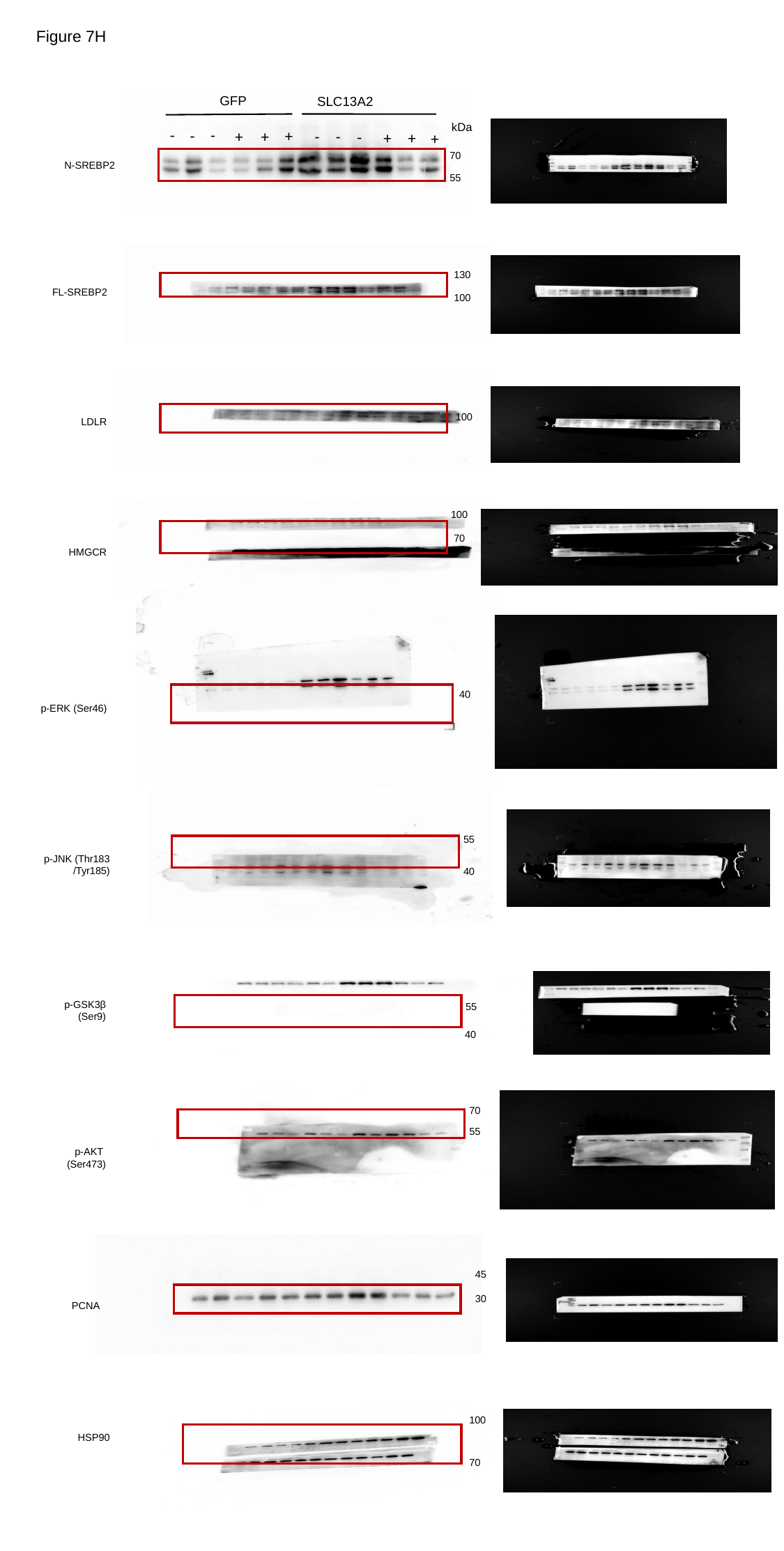

Figure 7H
GFP
SLC13A2
kDa
-
-
-
+
+
-
+
-
-
+
+
+
70
N-SREBP2
55
130
FL-SREBP2
100
100
LDLR
100
70
HMGCR
40
p-ERK (Ser46)
55
 p-JNK (Thr183
 /Tyr185)
40
p-GSK3β
(Ser9)
55
40
70
55
p-AKT
 (Ser473)
45
30
PCNA
100
HSP90
70
