# Supplementary material for: SLC13A2 promotes hepatocyte metabolic remodeling and liver regeneration by enhancing de novo cholesterol biosynthesis
Source: EMBO J. 2025 Jan 17;44(5):1442–63. doi: 10.1038/s44318-025-00362-y (PMC11876347; doi:10.1038/s44318-025-00362-y)
Supplement: Supplementary file 10 — Source data Fig. 8 [file 44318_2025_362_MOESM10_ESM.zip › Figure 8/8D.pptx]

## Slide 1
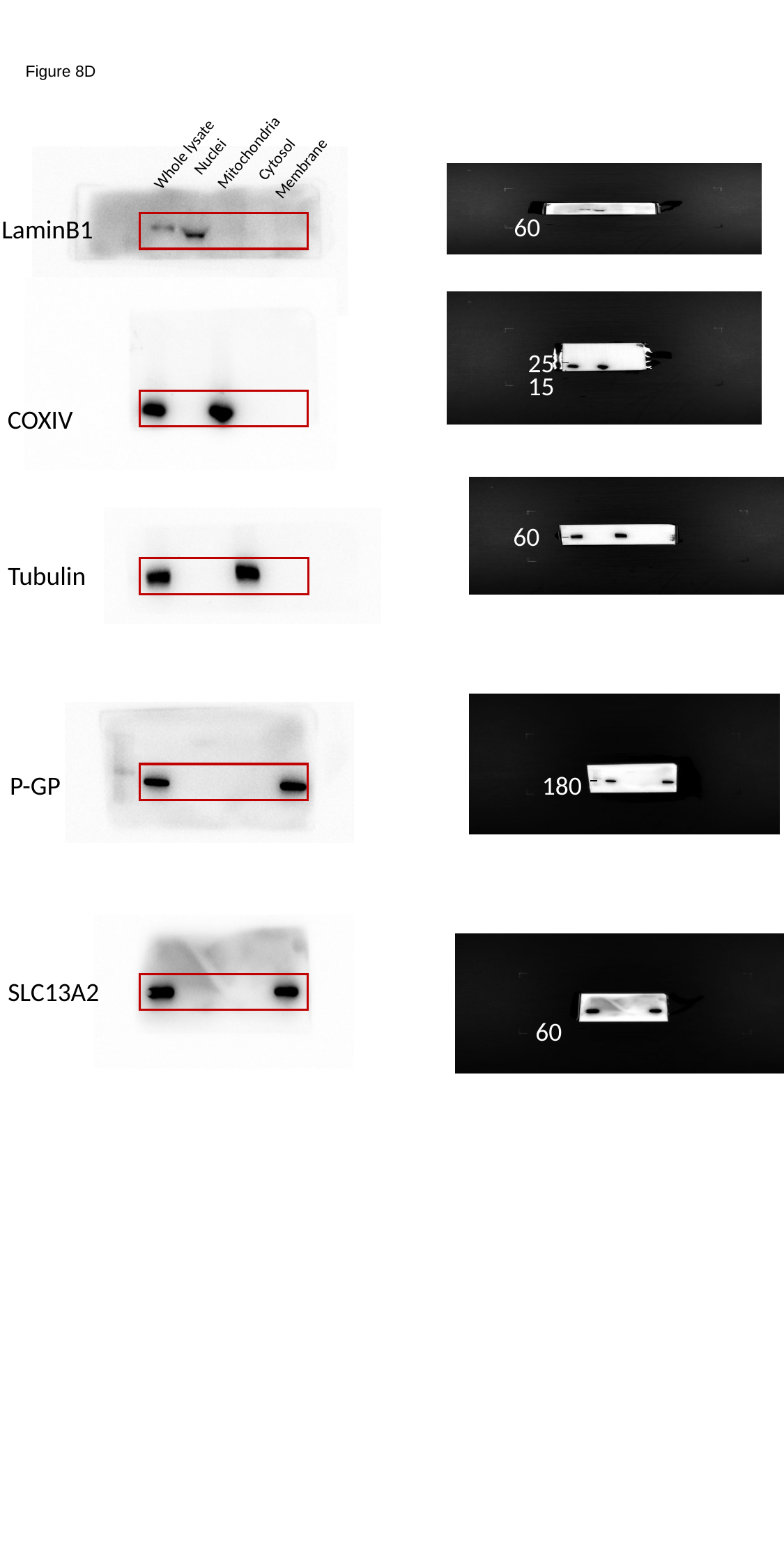

Figure 8D
Mitochondria
Whole lysate
Nuclei
Cytosol
Membrane
60
LaminB1
25
15
COXIV
60
Tubulin
180
P-GP
SLC13A2
60
