# Supplementary material for: SLC13A2 promotes hepatocyte metabolic remodeling and liver regeneration by enhancing de novo cholesterol biosynthesis
Source: EMBO J. 2025 Jan 17;44(5):1442–63. doi: 10.1038/s44318-025-00362-y (PMC11876347; doi:10.1038/s44318-025-00362-y)
Supplement: Supplementary file 10 — Source data Fig. 8 [file 44318_2025_362_MOESM10_ESM.zip › Figure 8/8I.pptx]

## Slide 1
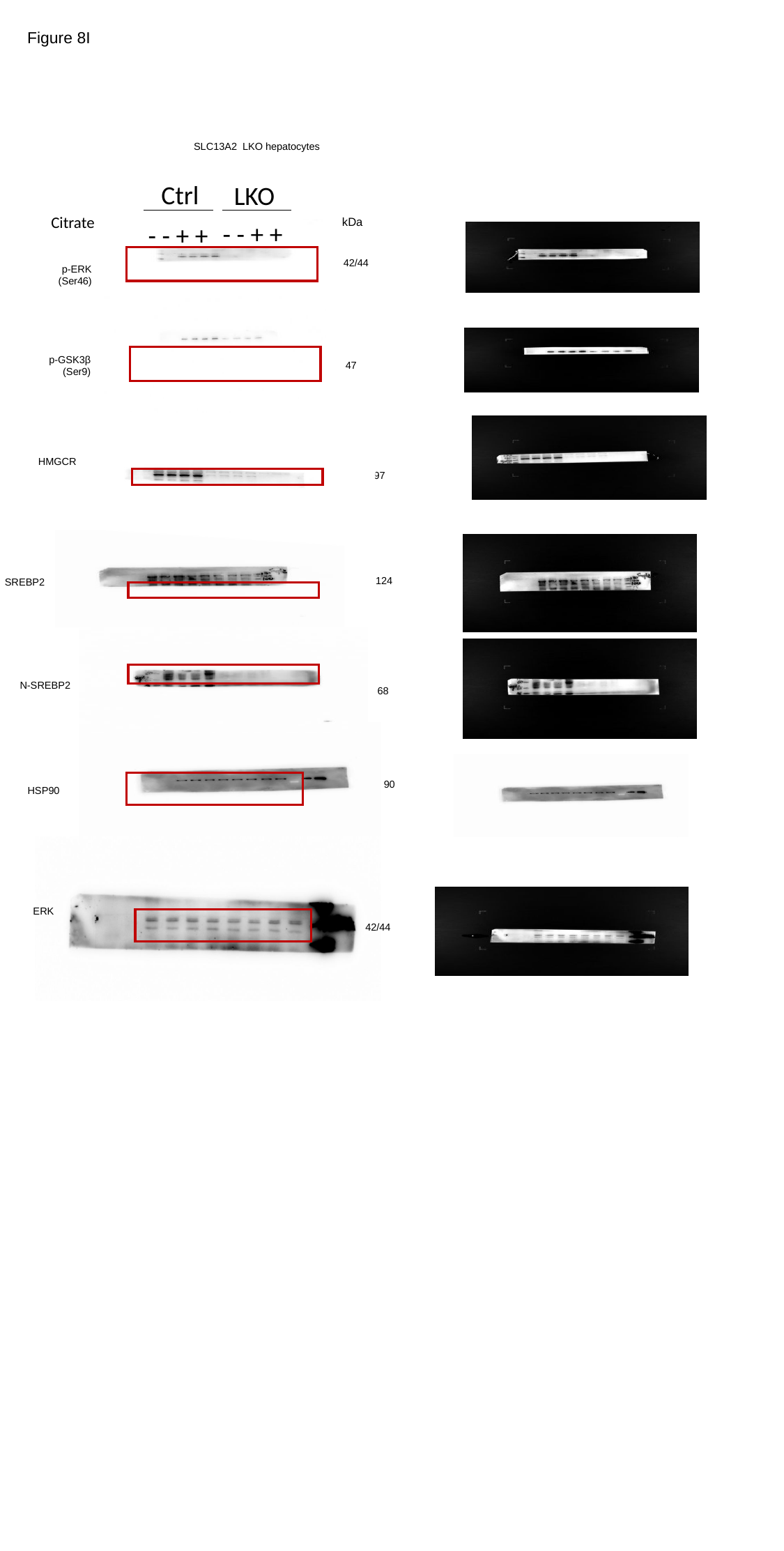

Figure 8I
SLC13A2 LKO hepatocytes
Ctrl
LKO
Citrate
kDa
 - - + +
 - - + +
42/44
p-ERK (Ser46)
p-GSK3β
(Ser9)
47
HMGCR
97
124
SREBP2
N-SREBP2
68
90
HSP90
ERK
42/44
