# Supplementary material for: SLC13A2 promotes hepatocyte metabolic remodeling and liver regeneration by enhancing de novo cholesterol biosynthesis
Source: EMBO J. 2025 Jan 17;44(5):1442–63. doi: 10.1038/s44318-025-00362-y (PMC11876347; doi:10.1038/s44318-025-00362-y)
Supplement: Supplementary file 10 — Source data Fig. 8 [file 44318_2025_362_MOESM10_ESM.zip › Figure 8/8C/LKO.pptx]

## Slide 1
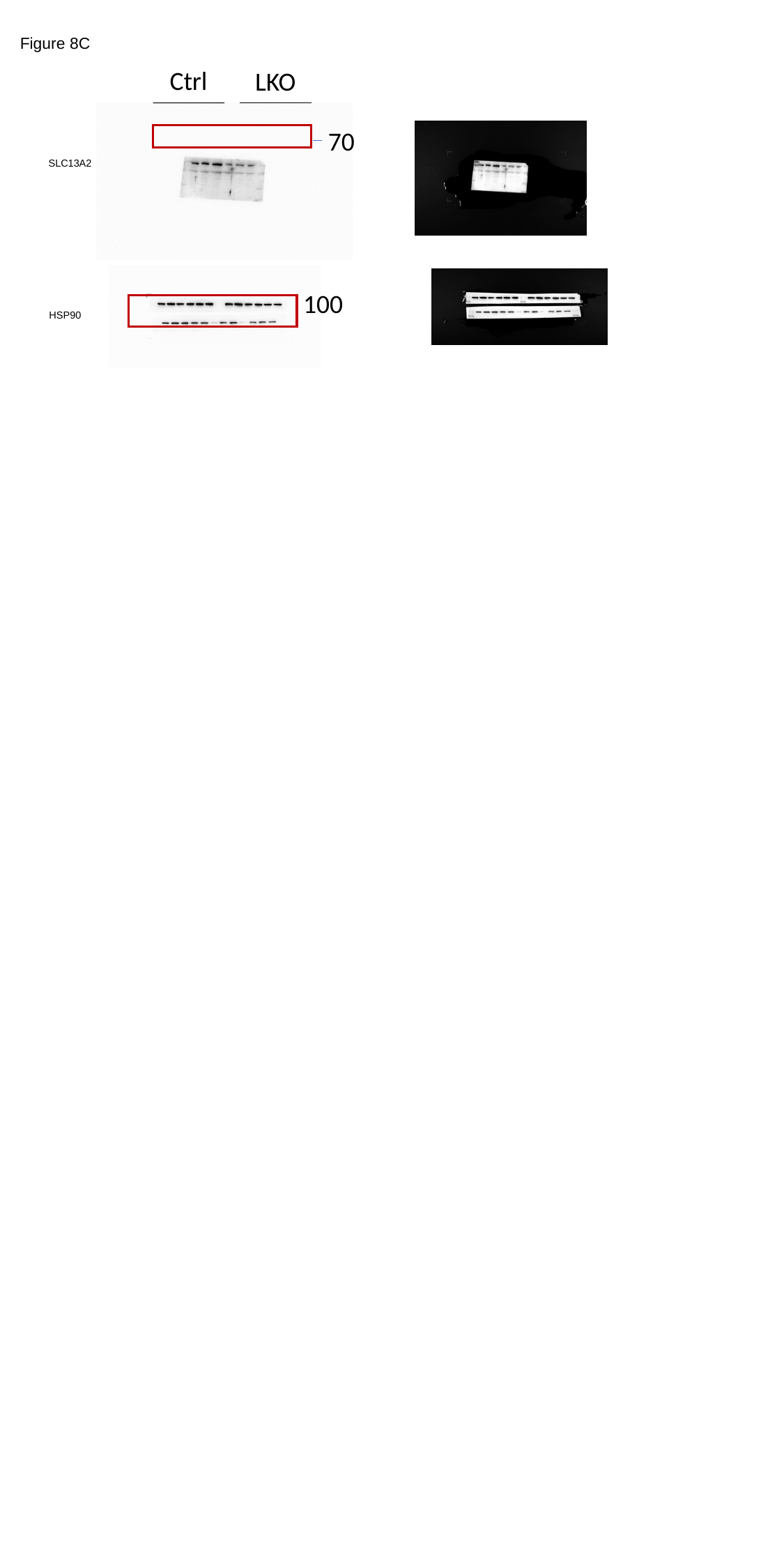

Figure 8C
Ctrl
LKO
70
SLC13A2
100
HSP90

## Slide 2
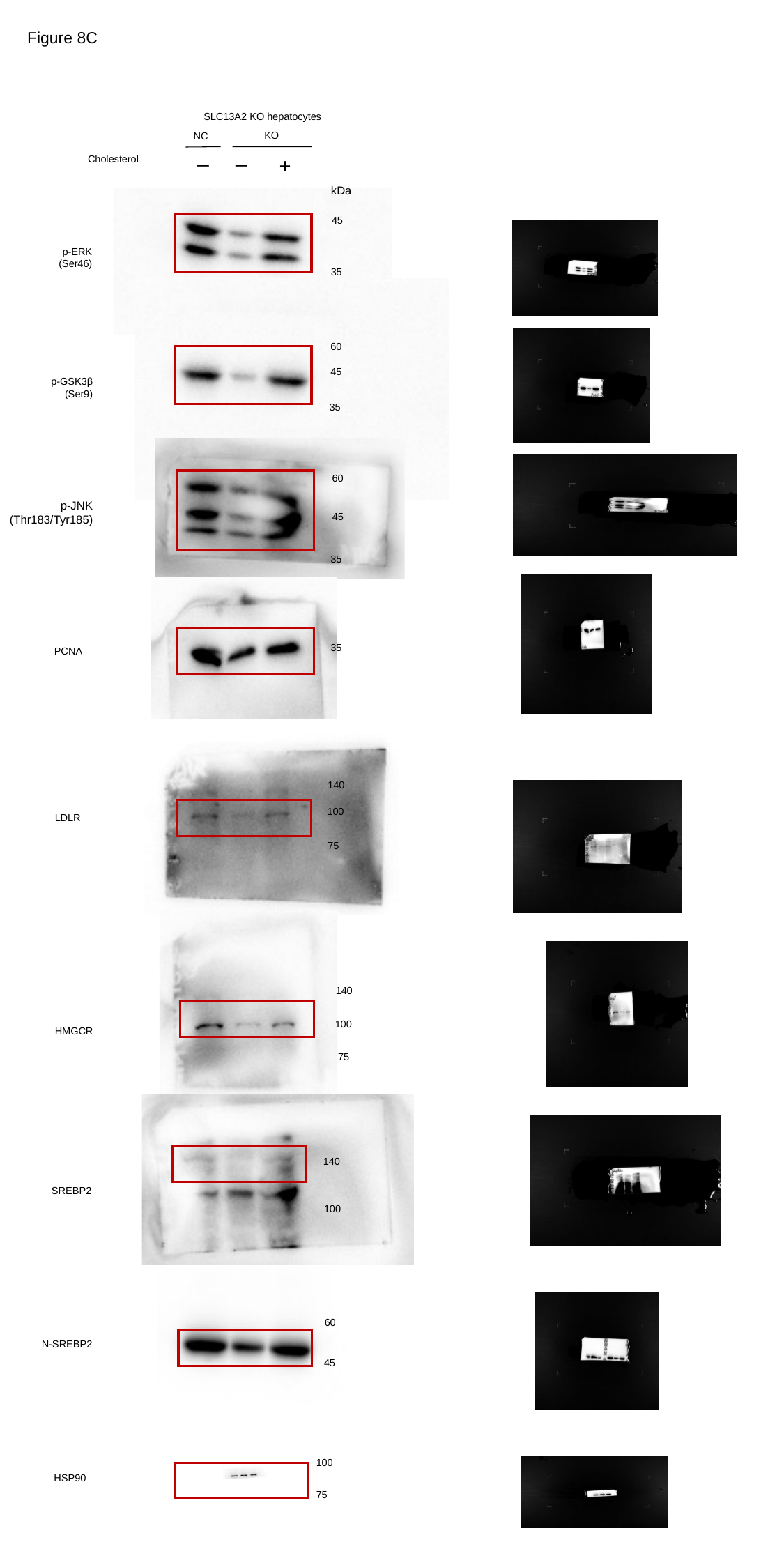

Figure 8C
SLC13A2 KO hepatocytes
KO
NC
_
_
Cholesterol
+
kDa
45
p-ERK (Ser46)
35
60
45
p-GSK3β
(Ser9)
35
60
p-JNK
(Thr183/Tyr185)
45
35
35
PCNA
140
100
LDLR
75
140
100
HMGCR
75
140
SREBP2
100
60
N-SREBP2
45
100
HSP90
75
