# Supplementary material for: SLC13A2 promotes hepatocyte metabolic remodeling and liver regeneration by enhancing de novo cholesterol biosynthesis
Source: EMBO J. 2025 Jan 17;44(5):1442–63. doi: 10.1038/s44318-025-00362-y (PMC11876347; doi:10.1038/s44318-025-00362-y)
Supplement: Supplementary file 11 — Source data Fig. 9 [file 44318_2025_362_MOESM11_ESM.zip › Figure 9/9H.pptx]

## Slide 1
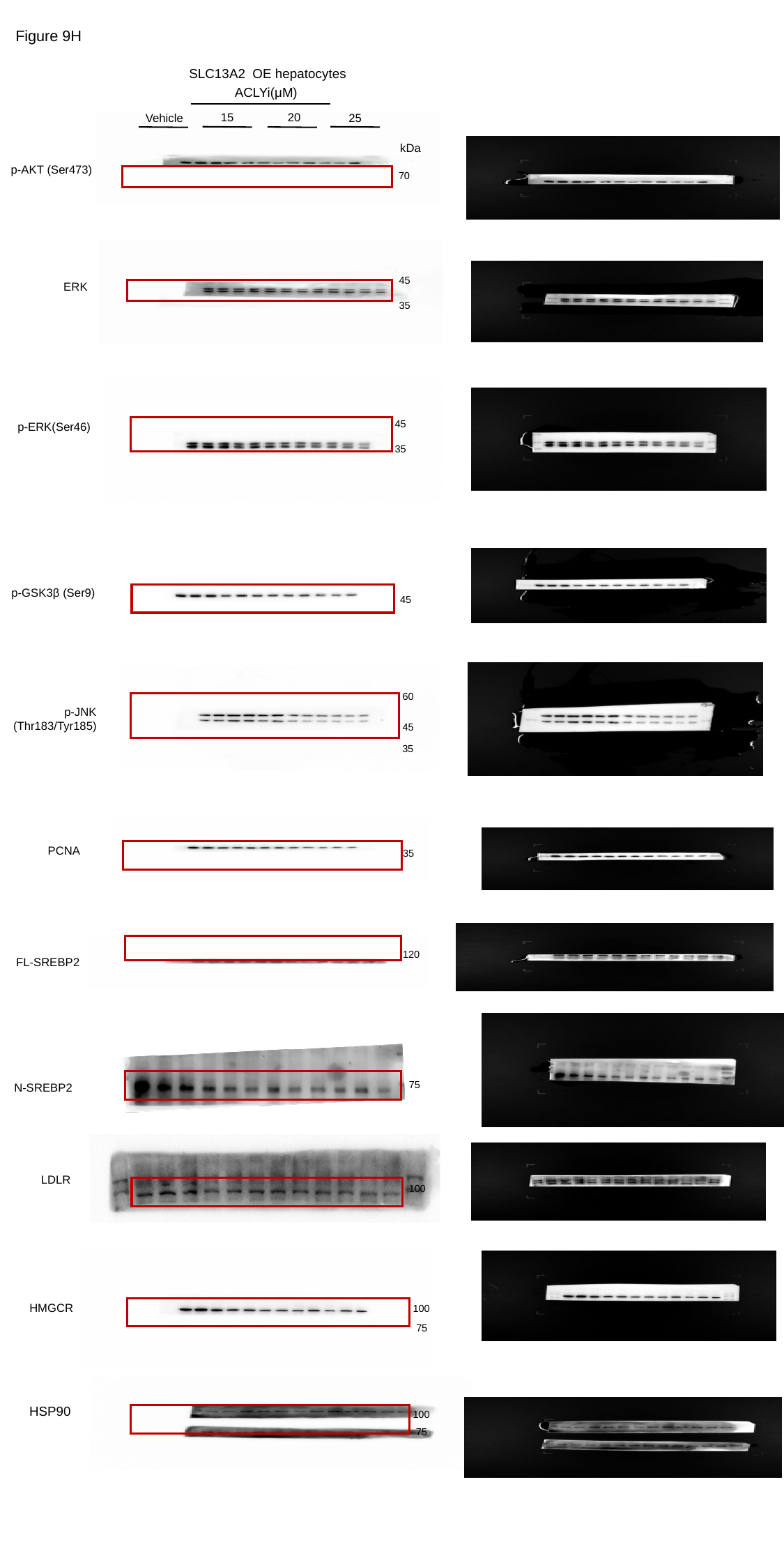

Figure 9H
SLC13A2 OE hepatocytes
ACLYi(μM)
15
20
25
Vehicle
kDa
p-AKT (Ser473)
70
45
ERK
35
45
p-ERK(Ser46)
35
p-GSK3β (Ser9)
45
60
p-JNK
(Thr183/Tyr185)
45
35
PCNA
35
120
FL-SREBP2
75
N-SREBP2
LDLR
100
HMGCR
100
75
HSP90
100
75
