# Supplementary material for: SLC13A2 promotes hepatocyte metabolic remodeling and liver regeneration by enhancing de novo cholesterol biosynthesis
Source: EMBO J. 2025 Jan 17;44(5):1442–63. doi: 10.1038/s44318-025-00362-y (PMC11876347; doi:10.1038/s44318-025-00362-y)
Supplement: Supplementary file 12 — Appendix figure legend [file 44318_2025_362_MOESM12_ESM.docx]

**Appendix Figure Legends**

**Appendix Figure S1. The expression of SLC transporters with similar functions of transporting TCA cycle metabolites (GSE95135).** The results demonstrate the decrease of SLC13A2 in the initiation and progression phase of liver regeneration after partial hepatectomy.

**Appendix Figure S2. Effects of liver-specific SLC13A2 knockout in quiescent liver.**

**A-C**. Body weight (A), liver weight (B) and blood glucose (C) of Ctrl and LKO mice.

**D.** qPCR analysis detected the expression of genes involving with glucose metabolism and lipid metabolism.

**E.** Representative H&E staining.

**F.** Immunoblots of total liver lysates.

Data are expressed as Mean ± SEM (N=7).

**Appendix Figure S3. Effects of liver-specific SLC13A2 overexpression without PHx surgery.**

**A-C**. Body weight (A), liver weight (B) and blood glucose (C) of Ctrl and SLC13A2-OE mice (N=6).

**D.** RNA expression of genes involving with glucose, lipid and glutamine metabolism was analyzed by qPCR assay (N=4).

**E.** Representative liver histological staining.

**F.** Immunoblots of total liver lysates.

Data are expressed as Mean ± SEM.

**Appendix Figure S4. Limited effects of SLC13A2 at 7d after the surgery of PHx.**

**A-F**. Liver regeneration was induced by PHx surgery with the overexpression of SLC13A2 and analysis was made after 7d. Body weight (A), blood glucose (B), iWAT weight (C), survival rate (D)，RNA expression of genes for cholesterol synthesis, glucose metabolism and cell proliferation (E) and H&E staining (F) of GFP and SLC13A2-OE mice (N=6).

**G-M**. Liver regeneration was induced by PHx surgery with the liver-specific deletion of SLC13A2 and analysis was made after 7d. Body weight (G), blood glucose (H), iWAT weight (I), survival rate (J)，Serum ALT/AST activity (K), RNA expression of genes for cholesterol synthesis, glucose metabolism and cell proliferation (L) and H&E staining (M) of Ctrl and SLC13A2-LKO mice (N=5).

Data are expressed as Mean ± SEM.

**Appendix Figure S5. SLC13A2 has limited influence on other metabolic pathways apart from cholesterol metabolism.**

**A-B.** RNA expression of genes involving glycolipid metabolism apart from cholesterol metabolism with the overexpression or deletion of SLC13A2 after PHX surgery (N=6).

**C.** Protein expression level of genes involving with lipid and cholesterol metabolism in indicated time points after PHX surgery.

**D.** Protein expression level of genes involving with lipid metabolism at 1d after PHX surgery in the mice hepatically deleted SLC13A2.

Data are expressed as Mean ± SEM.

**Appendix Figure S6. The effect of lovastatin on SLC13A2-promoted liver regeneration at 7d after PHx surgery.**

A. Diagrams of study design. Mice were injected with AAV-TBG-SLC13A2 virus (1 × 10^11^ VP/mouse) through tail vein to ensure the liver-specific overexpression of SLC13A2, followed by the intragastrical administration of Lovastain (4 mg/Kg) or vehicle once a day for consecutive 3 days. Then the surgery of PHx was operated, and the mice were sacrificed after 7 days.

B. Liver weight versus body weight ratio.

C. Blood glucose.

D. RNA expression of genes involving cholesterol synthesis and cell proliferation.

E. Liver contents and serum concentrations of TG/TC.

F. Representative images of H&E staining and BrdU incorporation.

Data are expressed as Mean ± SEM (Vehicle, N=5; Lovastain, N=6). *P < 0.05 Lovastatin vs Vehicle group, two-tailed unpaired Student’s *t* test.
